# Disease-driven post-transcriptional alterations and alternative splicing in podocytes in focal segmental glomerulosclerosis

**DOI:** 10.1038/s44321-026-00495-5

**Published:** 2026-08-13

**Authors:** Francescapaola Mattias, Olga Tsoy, Anna Iervolino, Stefan Simm, Elke Hammer, Alexander Gress, Florian Siegerist, Maximillian Schindler, Tim Lange, Sabine Ameling, Tim Kacprowski, Markus List, Olga Kalinina, Sören Franzenburg, Giovambattista Capasso, Karlhans Endlich, Jan Baumbach, Uwe Völker, Nicole Endlich, Felix Kliewe

**Affiliations:** 1 Department of Anatomy and Cell Biology, University Medicine Greifswald, Greifswald, Germany; 2 Institute for Computational Systems Biology, University of Hamburg, Hamburg, Germany; 3 Institute of Molecular Biology and Genetics (Biogem), Ariano Irpino, Italy; 4 Coburg University of Applied Sciences, Institute of Bioanalysis, Coburg, Germany; 5 Interfaculty Institute for Genetics and Functional Genomics, University Medicine Greifswald, Greifswald, Germany; 6 Department of Drug Bioinformatics, Helmholtz Institute for Pharmaceutical Research Saarland (HIPS), Helmholtz Centre for Infection Research (HZI), Saarbrücken, Germany; 7 Neonatology and Pediatric Intensive Care Medicine, Department of Pediatrics, University Medicine Greifswald, Greifswald, Germany; 8 German Center for Child and Adolescent Health (DZKJ), Partner Site Greifswald, Rostock, Germany; 9 Institute of Data Science in Biomedicine, Technische Universität Braunschweig, Braunschweig, Germany; 10 Braunschweig Integrated Centre of Systems Biology (BRICS), TU Braunschweig, Braunschweig, Germany; 11 Data Science in Systems Biology, TUM School of Life Sciences, Technical University of Munich, Freising, Germany; 12 Munich Data Science Institute, Technical University of Munich, Garching, Germany; 13 Institute of Clinical Molecular Biology (IKMB), Kiel University, Kiel, Germany; 14 Department of Translational Medical Science, University of Campania Luigi Vanvitelli, Naples, Italy; 15 NIPOKA GmbH, Center of High-End Imaging, Greifswald, Germany

**Keywords:** RNA Biology, Urogenital System

## Abstract

Focal segmental glomerulosclerosis (FSGS) is a major cause of nephrotic syndrome and progression to end-stage renal disease, yet its molecular pathogenesis remains still incompletely defined. While transcriptional alterations in podocytes have been extensively characterized, the contribution of post-transcriptional regulatory mechanisms is poorly understood. Here, we combined a zebrafish podocyte-specific injury model with glomerulus-resolved transcriptomic profiling to dissect RNA regulatory alterations during FSGS progression. Integrated analyses of bulk RNA sequencing, small RNA profiling, and alternative splicing revealed pronounced, time-dependent remodeling of the glomerular transcriptome. We demonstrate that podocyte injury is associated with loss of key podocyte-specific proteins, activation of inflammatory pathways, remodeling of the extracellular matrix, and altered microRNA expression, such as miR-21 and miR-193. Moreover, we found that alternative splicing influences key podocyte gene expression, affecting genes critical for slit diaphragm integrity, actin cytoskeleton organization, and glomerular basement membrane stability. Isoform analyses identified FSGS-associated isoform switches in SRSF3 and EPB41L5. Importantly, these changes were also evident in glomeruli from FSGS patients, demonstrating that the zebrafish model recapitulates key molecular features of human disease and highlighting alternative splicing as a central regulatory mechanism in FSGS.

## Synopsis

Post-transcriptional regulations, such as alternative splicing and microRNA dysregulation, are identified as central and underappreciated processes in injured podocytes in focal segmental glomerulosclerosis (FSGS), with disease-associated isoform switches in SRSF3 and EPB41L5.

- A zebrafish larval podocyte injury model reveals time-dependent, progressive remodeling of the glomerular transcriptome encompassing regulated miRNAs and hundreds of alternative splicing events at 5 and 6 days post fertilization.
- Exon skipping is identified as the predominant splicing event, preferentially affecting podocyte-enriched genes critical for slit diaphragm integrity, cytoskeletal organization, and glomerular basement membrane stability.
- A disease-associated isoform switch in the splicing factor SRSF3 toward an isoform lacking 12 C-terminal phosphorylation sites is detected in zebrafish FSGS glomeruli.
- A coordinated isoform switch in EPB41L5, accompanied by downregulation of polarity complex partners CRB2 and MPP5, is identified across zebrafish, mouse, and human FSGS datasets.
- Strong cross-species concordance between zebrafish, mouse, and human FSGS glomerular transcriptomes validates the zebrafish model as a platform for mechanistic and therapeutic investigation of post-transcriptional regulation in glomerular disease.

## Abstract (web summary)

Post-transcriptional regulations, such as alternative splicing and microRNA dysregulation, are identified as central and underappreciated processes in injured podocytes in focal segmental glomerulosclerosis (FSGS), with disease-associated isoform switches in SRSF3 and EPB41L5.

## Graphical abstract

**Figure:**
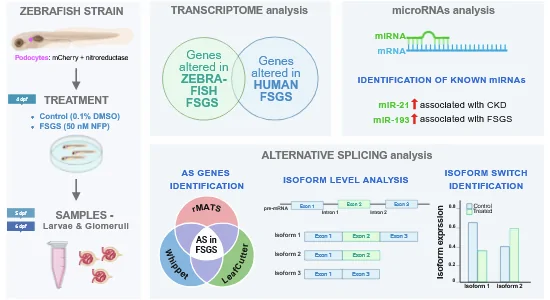

**Figure:**
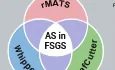

The paper explained **Problem**

Focal segmental glomerulosclerosis (FSGS) is a severe kidney disease that often leads to the loss of kidney function. It is driven by the dysfunction and irreversible loss of podocytes, specialized kidney filter cells that prevent protein from leaking into the urine. While transcriptional changes in podocytes have been studied extensively, post-transcriptional regulatory mechanisms such as microRNA regulation and alternative splicing remain poorly understood in FSGS.

**Results**

Using a zebrafish podocyte injury model combined with glomerulus-resolved RNA sequencing, we identify widespread and time-dependent post-transcriptional remodeling during FSGS progression. Beyond dysregulation of disease-relevant microRNAs (miR-21, miR-193), we demonstrate extensive alternative splicing affecting core structural podocyte genes. Under disease conditions, EPB41L5, a regulator of epithelial polarity and cell-cell contacts, is downregulated in podocytes and switches toward a truncated isoform, coinciding with downregulation of its interacting partners CRB2 and MPP5, key proteins required for podocyte polarity and integrity. The disease-induced isoform switch of SRSF3 resulted in a variant lacking the serine-rich C-terminal domain, which contains 12 phosphorylation sites required for SRSF3 autoregulation. Key findings of the study were validated across human FSGS and mouse glomerular datasets, supporting cross-species conservation of the identified regulatory changes. Consistently, human kidney biopsies showed increased nuclear SRSF3 and reduced EPB41L5 protein levels in FSGS compared with healthy controls.

**Impact**

These findings establish alternative splicing as a central regulatory mechanism in FSGS and identify SRSF3 and EPB41L5 isoform switches as promising candidates for disease biomarkers and therapeutic targeting.

## Introduction

Focal segmental glomerulosclerosis (FSGS) is a common histopathological pattern underlying nephrotic syndrome and a major cause of progression to end-stage renal disease in both children and adults (Ellison et al, 2024; McGrogan et al, 2011; Rosenberg and Kopp, 2017). Despite advances in genetic diagnostics, molecular profiling, and immunosuppressive treatment strategies, disease progression remains poorly controlled in a substantial proportion of patients. This highlights a critical gap in our understanding of the molecular mechanisms that drive podocyte dysfunction and irreversible glomerular damage.

Histologically, FSGS is characterized by segmental scarring of a subset of glomeruli, resulting in proteinuria and progressive loss of renal function (Schwartz and Korbet, 1993; Shabaka et al, 2020). At the cellular level, podocytes represent the primary site of injury. These highly specialized postmitotic epithelial cells are essential for maintaining the integrity of the glomerular filtration barrier. As these cells are unable to regenerate, prolonged dysfunction or loss of podocytes is a hallmark of FSGS progression (Greka and Mundel, 2012).

In this context, numerous studies have focused on genetic mutations and transcriptional alterations in podocytes, whereas post-transcriptional regulatory mechanisms have been less studied in FSGS. Non-coding RNAs, particularly microRNAs, have emerged as important regulators of podocyte biology and glomerular disease, yet they represent only one layer of RNA regulation (Trionfini and Benigni, 2017; Lange et al, 2019; Gebeshuber et al, 2013). Alternative splicing is a fundamental mechanism that expands proteomic diversity and enables context- and cell-type-specific gene regulation. Tight control of splicing decisions is particularly important in highly specialized cells such as podocytes, where subtle changes in protein isoform composition can have profound functional consequences (Das et al, 2025; Mattias et al, 2025).

Alternative splicing is coordinated by the spliceosome and auxiliary splicing factors, including serine/arginine-rich (SR) proteins and heterogeneous nuclear ribonucleoproteins (hnRNPs) (Busch and Hertel, 2012). Among these, SRSF3 has emerged as a key regulator of splice-site selection, RNA stability, and transcript export. SRSF3 activity is tightly regulated by phosphorylation within a conserved serine-rich C-terminal domain, which modulates its interaction with RNA and other components of the splicing machinery (Long et al, 2019; Zheng et al, 2020). Dysregulation of SRSF3 expression or function has been implicated in cancer, metabolic disease, and inflammatory signaling (More and Kumar, 2020); however, its role in podocyte biology and glomerular disease remains largely unexplored.

Perturbations of splicing fidelity, through altered expression of splicing factors, disease-induced isoform switching, or imbalanced phosphorylation states, can result in the production of dysfunctional or pathogenic protein isoforms. Whether such disease-driven splicing alterations actively contribute to podocyte injury and FSGS progression in vivo has not been systematically investigated.

High-throughput RNA sequencing provides a powerful approach to comprehensively assess gene expression, microRNA regulation, and alternative splicing within complex tissues. Zebrafish (*Danio rerio*) larvae represent an established and ethically advantageous model for kidney research, as their pronephros exhibits high structural and functional similarity, including conserved gene expression profiles to the human glomerulus (Schindler and Endlich, 2024). We have previously established a zebrafish model of FSGS that recapitulates key pathological features of human disease, including podocyte depletion, activation of parietal epithelial cells, and glomerular basement membrane remodeling (Hansen et al, 2020; Schindler et al, 2023; Klawitter et al, 2024).

In the present study, we used a podocyte-specific injury model induced by Nifurpirinol (NFP) in genetically modified zebrafish larvae to investigate post-transcriptional regulatory mechanisms during FSGS progression. By integrating bulk RNA sequencing, small RNA profiling, and comprehensive alternative splicing analyses in whole larvae and isolated glomeruli, we identify coordinated dysregulation of microRNAs and alternative splicing as central features of podocyte injury.

Importantly, our data revealed a disease-associated isoform switch of the splicing factor SRSF3, positioning it as a potential regulatory hub linking podocyte injury to widespread alterations of splicing. These findings provide new insight into posttranscriptional RNA regulation in FSGS and propose splicing factor isoforms as promising candidates for biomarker development and therapeutic targeting.

## Results

### Transcriptome analysis of whole zebrafish larvae and isolated glomeruli after FSGS induction

**Figure 1:**
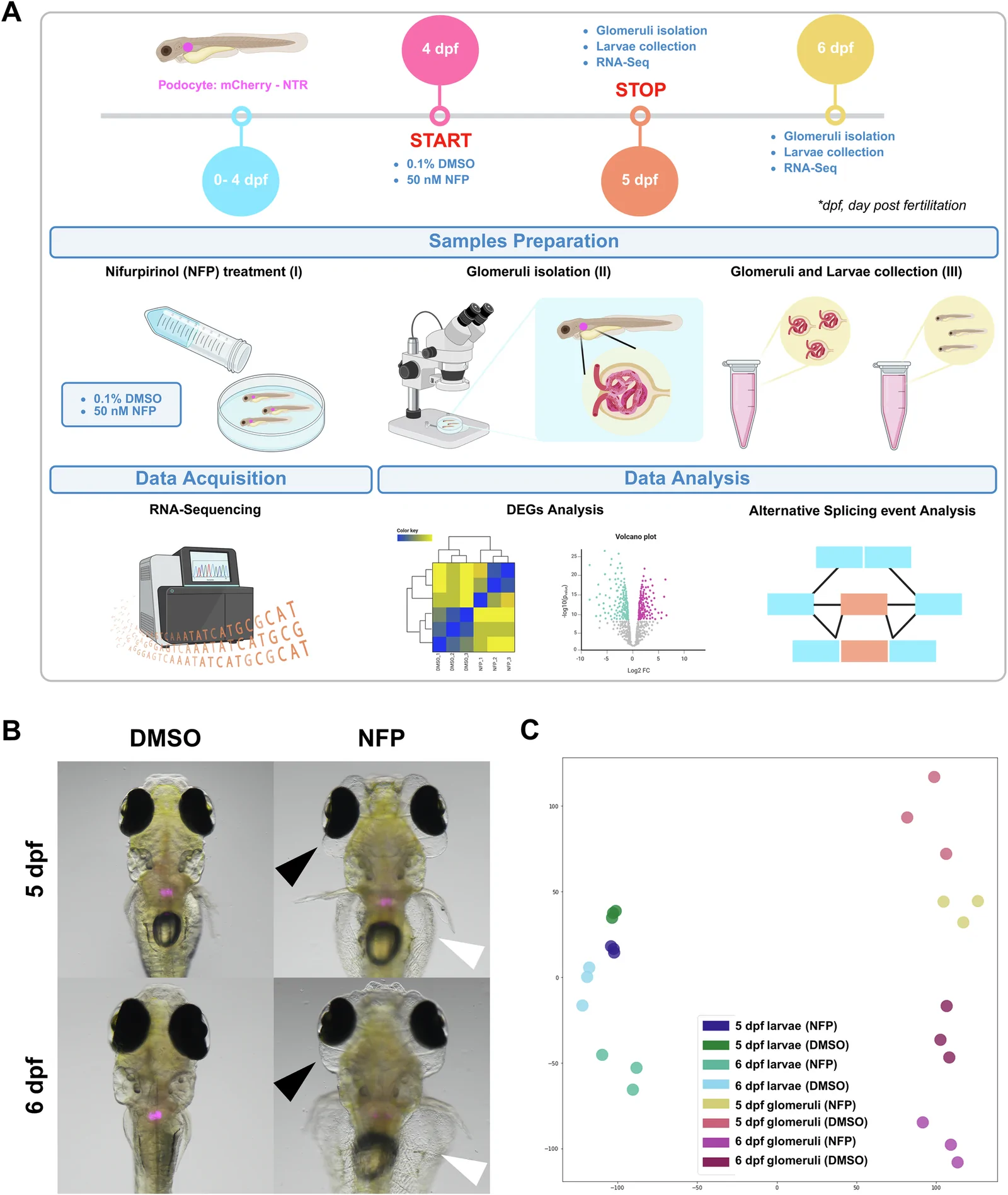
Overview of the experimental design of the FSGS model. (**A**) Representation of the experimental design with: timeline, sample preparation and data acquisition. (**B**) Control-treated and NFP-treated zebrafish mCherry larvae at 5 and 6 dpf. The black arrowhead indicates periocular edema, the white arrowhead indicates abdominal edema. (**C**) Scatter plot shows the result of Principal Component Analysis (PCA) of gene expression levels of NFP- and DMSO-treated larvae and glomeruli at 5 and 6 dpf. Sample classes (as set with the interesting groups argument) are highlighted in different colors. Source data are available online for this figure.

To investigate post-transcriptional regulatory mechanisms during FSGS progression, we used a podocyte-specific injury model based on Nifurpirinol (NFP) treatment in transgenic zebrafish larvae expressing nitroreductase (NTR) and mCherry exclusively in podocytes [Tg(nphs2:GAL4-VP16); Tg(UAS:Eco.nfsB-mCherry)]. NFP treatment at 50 nM from 4 days post fertilization (dpf) onwards induced selective podocyte ablation, resulting in progressive periocular and abdominal edema and loss of mCherry fluorescence, consistent with previously published characterizations of this model (Klawitter et al, 2024; Schindler et al, 2023). Whole larvae and manually isolated glomeruli were collected at 5 and 6 dpf, corresponding to 24 h and 48 h post-treatment, respectively, for parallel RNA-seq, small RNA profiling, and alternative splicing analysis (Fig. 1A–C).

**Figure 2:**
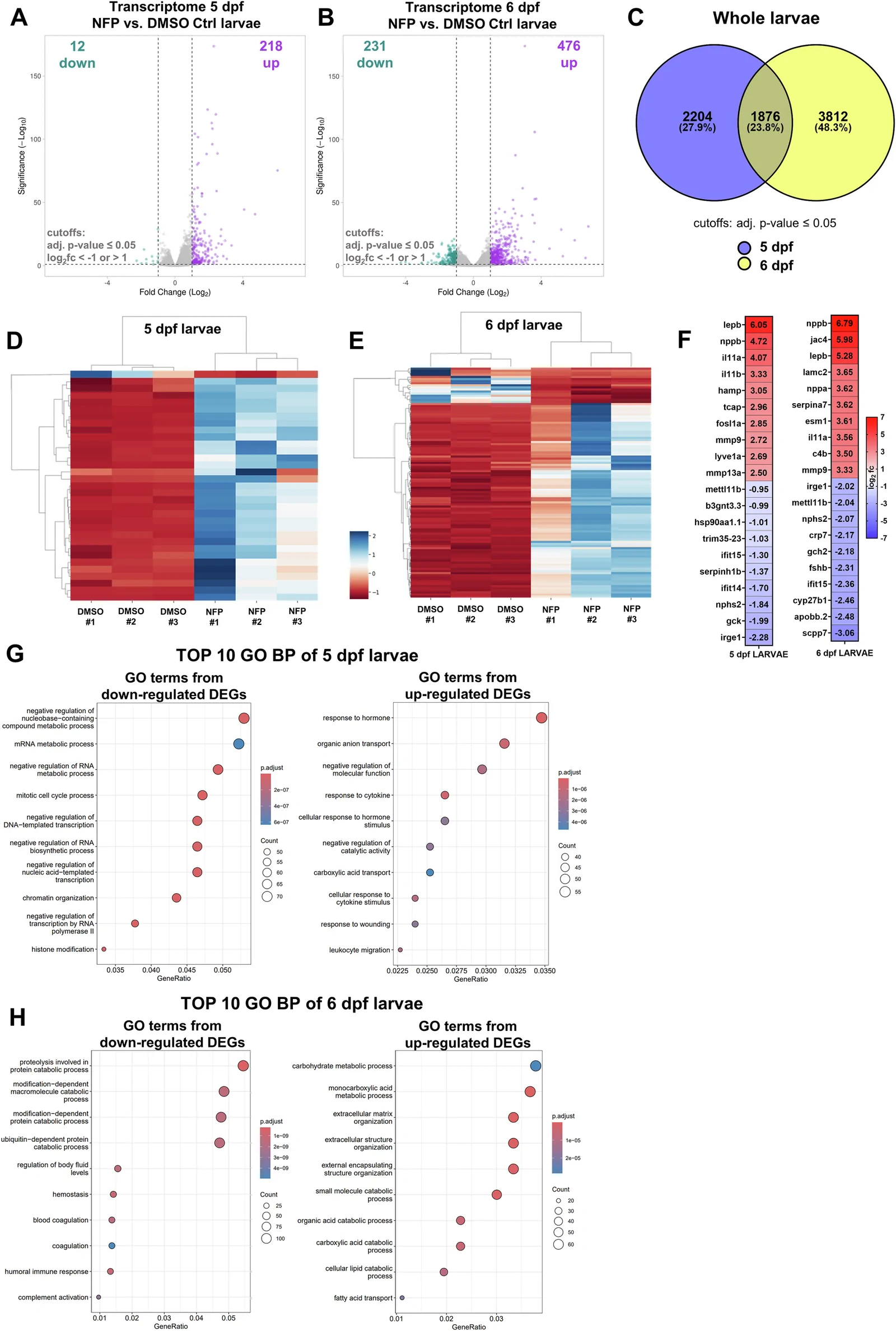
Transcriptomic analysis of zebrafish larvae. (**A**, **B**) Volcano plot of differentially expressed genes (DEGs) in NFP-treated zebrafish larvae at 5 dpf (**A**) and 6 dpf (**B**) compared to the control group (*n* = 3 independent biological replicates per condition). The purple dots and the cyan dots represent significant upregulated and downregulated DEGs (log2 fold-change ≥ 1; adjusted *P* value ≤ 0.05). The grey dots represent genes with no significant differences. (**C**) Venn diagram shows the significantly DEGs at 5 and 6 dpf after NFP treatment and their overlap (adj. *P* value ≤ 0.05). Note that gene counts in the Venn diagram exceed those in the volcano plots, as the Venn diagram applies a threshold of adjusted *P* value ≤ 0.05 without an additional fold-change filter, whereas the volcano plots require both adjusted *P* value ≤ 0.05 and log2FC ≥ 1. (**D**, **E**) Heatmaps of gene expression between NFP- and DMSO- treated larvae at 5 dpf (**D**) and 6 dpf (**E**) based on hierarchical clustering analysis. Heatmaps colors represent relative gene expression as indicated in the color key. (**F**) Heatmaps show the top 10 up- and downregulated genes present in zebrafish larvae at 5 and 6 dpf that show significant changes in gene level due to NFP treatment. Data are expressed as log2 fold change, a *P* value < 0.05 was considered as significant. Red: upregulated; blue: downregulated compared to controls. (**G**, **H**) The top 10 biological processes (BP) enriched in DEGs of NFP-treated larvae at 5 (**G**) and 6 (**H**) dpf compared to the respective control. The size of the dots represents the number of genes and the color of the dots represent the adjusted *P* values. Differential expression analysis was performed using DESeq2. Statistical significance was assessed using the Wald test, and *P* values were adjusted for multiple testing using the Benjamini–Hochberg method. Source data are available online for this figure.

Bulk RNA sequencing (three independent biological replicates per condition) of isolated glomeruli revealed a progressive transcriptional response to podocyte ablation at 5 and 6 dpf. For the RNA sequencing of whole larvae at 5 dpf, 230 (adjusted *P* ≤ 0.05; log2FC ≥ 1) differentially expressed genes (DEGs) were detected, with the majority being upregulated (Fig. 2A). At 6 dpf, the number of DEGs increased substantially to 707, indicating an amplification of the molecular injury response over time (Fig. 2B). Overlap analysis showed that a subset of DEGs was shared between both time points, whereas a large fraction was stage-specific, consistent with time-dependent regulation (Fig. 2C). Hierarchical clustering confirmed robust separation of FSGS-induced and control samples at both stages (Fig. 2D,E). The most strongly regulated transcripts at each time point are summarized in Fig. 2F.

Gene ontology (GO) enrichment analysis indicated distinct functional signatures at early versus later disease stages. At 5 dpf, downregulated genes were particularly enriched for processes involved in the regulation of gene expression and chromatin dynamics, whereas upregulated genes were predominantly associated with cytokine- and hormone-related signaling pathways (Fig. 2G; Appendix Fig. S1). At 6 dpf, downregulated DEGs were frequently associated with immune homeostasis-related processes, whereas upregulated DEGs were linked to metabolic pathways (Fig. 2H; Appendix Fig. S1).

**Figure 3:**
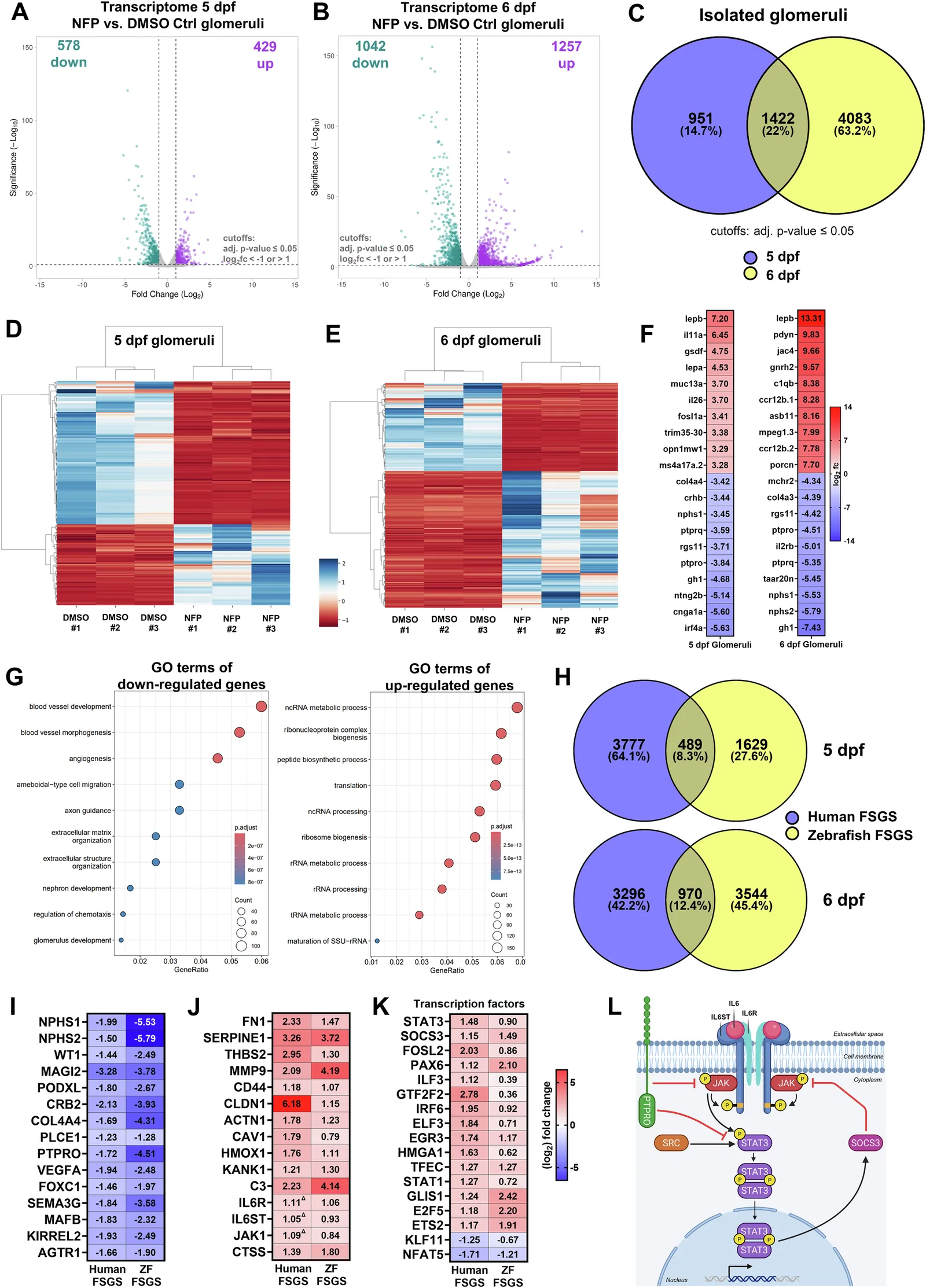
Transcriptomic analysis of isolated zebrafish glomeruli. (**A**, **B**) Volcano plot showing differentially expressed genes (DEGs) in isolated glomeruli of zebrafish larvae at 5 dpf (**A**) and 6 dpf (**B**) (NFP vs. DMSO; *n* = 3 independent biological replicates per condition). The purple dots and the cyan dots represent significantly up- and downregulated DEGs (log2 fold-change ≥ 1; adjusted *P* value ≤ 0.05). The grey dots represent genes with no significant differences. (**C**) Venn diagram depicts the number of the common and distinct DEGs at 5 and 6 dpf in isolated glomeruli (NFP vs Ctrl) (adj. *P* value ≤ 0.05). The total gene count in the Venn diagram represents the union of DEGs across both time points; individual time point counts are shown in (**A**, **B**). (**D**, **E**) Heatmaps of hierarchical clustering for gene expression in isolated glomeruli at 5 dpf (**D**) and 6 dpf. Heatmaps colors represent relative gene expression as indicated in the color key. (**F**) Heatmaps of the top 10 most significantly up- and downregulated DEGs at 5 and 6 dpf. Data are expressed as log2 fold change, a *P* value < 0.05 was considered as significant. Red: upregulated; blue: downregulated compared to controls. (**G**) Top 10 enriched biological processes for DEGs at 6 dpf based on Gene Ontology analysis. Dot size represents gene count; color indicates *P*-adjusted values. (**H**) Venn diagrams showing shared DEGs between human FSGS glomeruli (defined as genes with adj. *P* value ≤ 0.05 in the Hodgin FSGS Glom Dataset, accessed from Nephroseq; Hodgin et al, 2010) and zebrafish glomeruli at 5 dpf and 6 dpf. (**I**–**K**) Heatmaps depicts significantly downregulated (**I**) and upregulated (**J**) genes, as well as putative FSGS-associated transcription factors (**K**) identified in both FSGS patients and in our FSGS model at 6 dpf. Data shows glomerular expression as log2 fold change (ZF FSGS) or unlogged fold change (human FSGS) (Δ: non-significant). (**L**) Schematic representation of the IL6/STAT3 signaling pathway under FSGS conditions. Upregulation of IL6R, IL6ST, and JAK1 leads to enhanced STAT3 activation via phosphorylation. The phosphorylated form (pSTAT3) translocated to the nucleus and induced the expression of downstream targets such as SOCS3. The downregulation of PTPRO impairs dephosphorylation of STAT3. Differential expression analysis was performed using DESeq2. Statistical significance was assessed using the Wald test, and *P* values were adjusted for multiple testing using the Benjamini–Hochberg method. Source data are available online for this figure.

To investigate glomerulus-specific responses induced by FSGS, we characterized isolated glomeruli from diseased and control larvae at 5 and 6 dpf. In contrast to whole larvae, isolated glomeruli showed a substantially higher number of DEGs, with 1007 DEGs at 5 dpf and 2299 DEGs at 6 dpf (Fig. 3A–C). Hierarchical clustering resulted in a clear group separation, as shown in Fig. 3D,E. Among the most highly regulated genes were canonical podocyte markers, such as *nphs1*, *nphs2*, and *ptpro* (Fig. 3F). GO enrichment analysis of the downregulated glomerular DEGs revealed that these were primarily associated with developmental processes in the nephron and glomerulus, whereas the upregulated genes showed an enrichment in RNA processing and ribosome biogenesis (Fig. 3G; Appendix Figs. S2 and S3).

### FSGS-associated genes identified in zebrafish are also regulated in humans

To cross-validate our zebrafish findings with human disease data, we accessed publicly available glomerular expression datasets from the Nephroseq platform, specifically the Hodgin FSGS Glom Dataset (Data ref: Hodgin et al, 2010) for human FSGS comparisons, and the Lindenmeyer Normal Tissue Panel (Data ref: Lindenmeyer et al, 2010) for glomerular enrichment analyses in healthy human tissue.

**Figure EV1:**
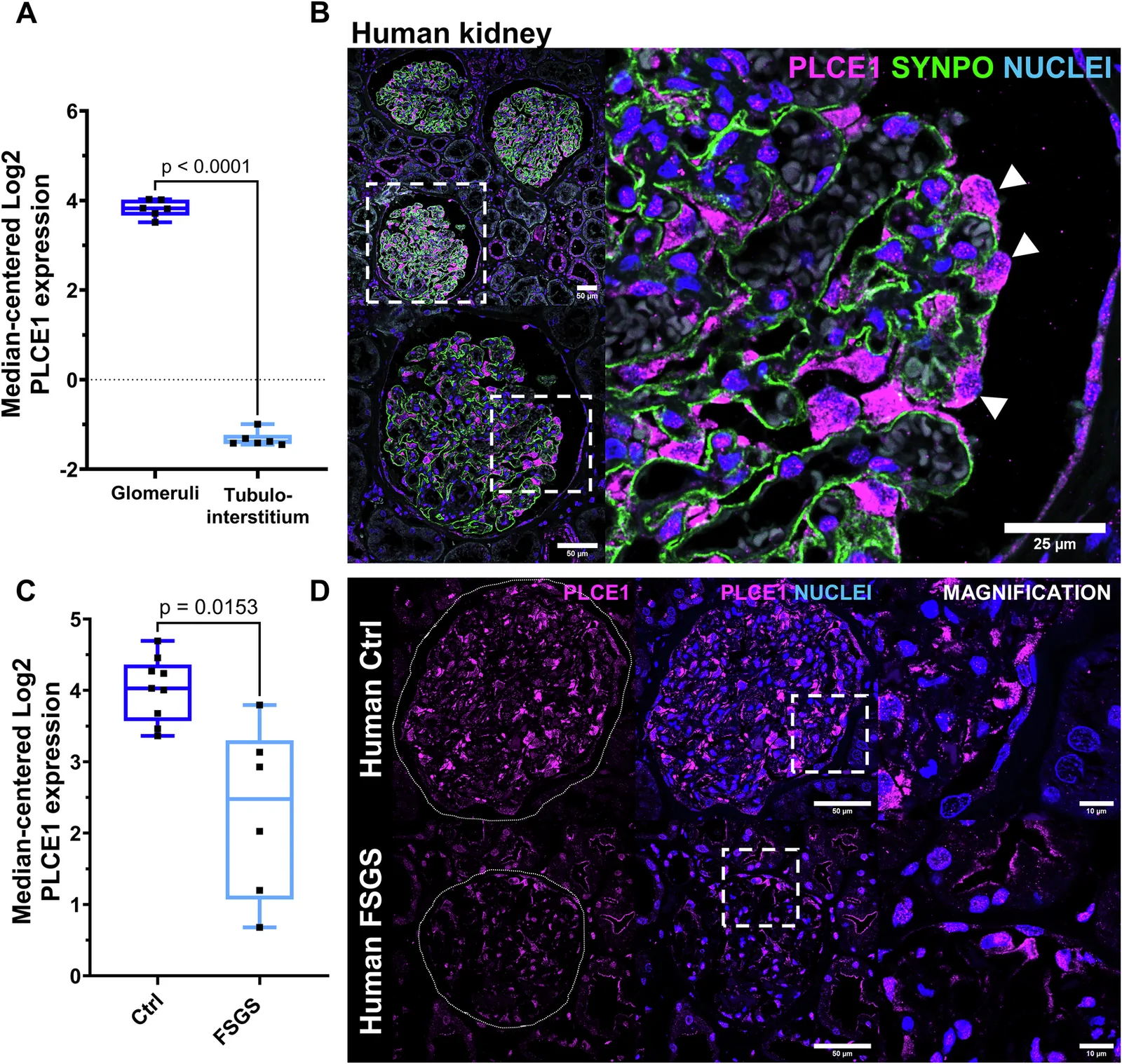
*PLCE1* expression in healthy human kidney and FSGS patients. This figure validates *PLCE1* as a podocyte-enriched gene that is downregulated in human FSGS, supporting the cross-species concordance shown in Fig. 3I of the main text. Human data were retrieved from the Nephroseq platform (Lindenmeyer Normal Tissue Panel for (**A**) (Data ref: Lindenmeyer et al, 2010) and Hodgin FSGS Glom Dataset for panel C (Data ref: Hodgin et al, 2010)). (**A**) Microarray data showing significantly higher *PLCE1* expression in glomeruli (*n* = 7) vs. tubulointerstitium (*n* = 7) in healthy human kidney tissue. (**C**) Glomerular *PLCE1* mRNA expression in healthy control (Ctrl) (*n* = 9) vs. FSGS (*n* = 6) patients. (**B**, **D**) Immunofluorescence for PLCE1 (magenta), synaptopodin (green), and DAPI (blue) in healthy human kidney (**B**) and FSGS biopsy (**D**). Podocytes are indicated by arrowheads. Scale bars: 50 µm, 25 µm, 10 µm. Statistical significance was assessed using Welch’s *t* test (**A**, **C**); *P* values is indicated. Box plots show the median (center line), 25th and 75th percentiles (box boundaries), and minimum and maximum values (whiskers) (**A**, **C**).

As shown in Fig. 3H, we found a substantial overlap in genes differentially expressed in human FSGS glomeruli (adj. *P* value ≤ 0.05): 489 DEGs at 5 dpf and 970 DEGs at 6 dpf. Among these DEGs, key podocyte differentiation markers such as *nphs1*, *nphs2*, *wt1*, *magi2*, *podxl*, *crb2*, *ptpro* and *plce1* were downregulated (Fig. 3I). Microarray data from healthy humans showed that phospholipase C epsilon 1 (*PLCE1*), a regulator of phosphoinositide signaling pathway, is highly expressed in glomeruli in contrast to the tubulointerstitium (Fig. EV1A). Immunofluorescence staining showed that PLCE1 is strongly localized in the cell body of podocytes (Fig. EV1B). In biopsies of FSGS patients, we observed a downregulation of PLCE1 which is consistence with the findings in glomeruli of larvae after FSGS induction (Fig. EV1C,D).

*Foxc1* and *mafb*, both transcription factors expressed in podocytes, were downregulated in injured larval glomeruli as well as glomeruli of FSGS patients (Fig. 3I). On the other hand, *fn1*, *serpine1*, *thbs2*, *actn1*, and *kank1*, all genes associated with podocyte injury, were consistently upregulated in both zebrafish and human FSGS glomeruli (Fig. 3J). This highlights the strong conservation of injury-associated pathways across species.

### STAT3 signaling is activated during FSGS but its inhibition fails to improve disease outcome

Since inflammation plays a central role during FSGS, we examined the IL6/JAK/STAT3 axis in our FSGS model. We found a significant upregulation of *stat3* and its downstream target *socs3* in injured zebrafish glomeruli and glomeruli of FSGS patients (Fig. 3K). Since we found a significant upregulation of the pro-inflammatory mediators *il6r*, *il6st*, and *jak1* in zebrafish FSGS glomeruli, we hypothesized an increased STAT3 phosphorylation in the cytosol, followed by an enhanced nuclear import, as illustrated in Fig. 3L. In parallel, we found *ptpro*, a negative regulator of STAT3, was downregulated, which could prolong STAT3 activation (Fig. 3I,L).

**Figure EV2:**
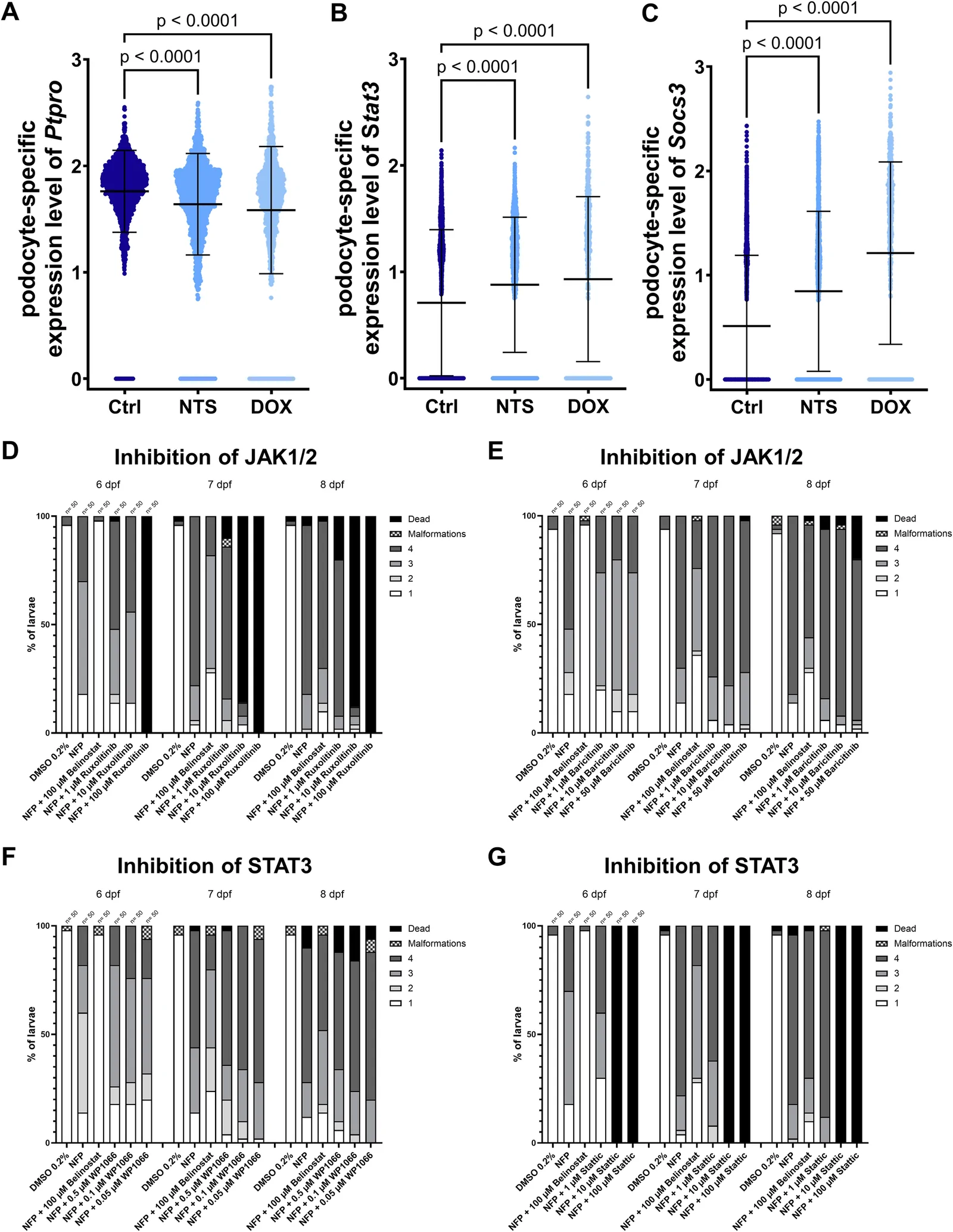
Stat3 expression increases in different kidney injury models, but inhibition of the JAK1/2-STAT3 pathway does not confer protective effects after FSGS induction in zebrafish larvae. This figure provides cross-species validation of STAT3 pathway activation (**A**–**C**) and documents the results of pharmacological inhibition experiments (**D**–**G**) referenced in the main text. (**A**–**C**) Podocyte-specific expression of *Ptpro* (**A**), *Stat3* (**B**), and *Socs3* (**C**) from published mouse glomerular scRNA-seq data (Chung et al, 2020) in nephrotoxic serum nephritis (NTS) and doxorubicin (DOX) models vs. control (*Ptpro*: Ctrl: *n* = 2018; NTS: *n* = 1639; DOX: *n* = 1434; *Stat3*: Ctrl: *n* = 2018; NTS: *n* = 1434; DOX: *n* = 504; *Socs3*: Ctrl: *n* = 2066; NTS: *n* = 1434; DOX: *n* = 504). (**D**–**G**) Bar graphs showing edema severity scores in zebrafish larvae (*n* = 50 per group) at 6, 7, and 8 dpf after NFP treatment (50 nM) co-administered with JAK1/2 inhibitors (Ruxolitinib, **D**; Baricitinib, **E**) or STAT3 inhibitors (WP1066, **F**; Stattic, **G**). Edema scores: 1  = no edema, 2 = mild edema, 3 = medium edema, 4 = severe edema with bent body axis and malformations or dead. Belinostat (100 µM) served as positive control. All inhibitors were administered concomitantly with NFP from 4 dpf. None of the inhibitors showed protective effect. Instead, treatment accelerated disease progression in the FSGS injury model, as evidenced by enhanced edema formation and increased mortality rates. Statistical significance was assessed using one-way ANOVA followed by Benjamini–Hochberg correction (**A**– **C**); *P* values are indicated; mean ± SD.

Furthermore, we analyzed published single-cell RNA-sequencing (scRNA-seq) data which were generated from mouse glomeruli (Chung et al, 2020), in which FSGS was induced by treatment with nephrotoxic serum (NTS) or doxorubicin. Here, we found similar increases in *Stat3* and *Socs3* expression in podocytes (Fig. EV2B,C).

To test whether pharmacological inhibition of the activated Stat3 pathway attenuates podocyte injury, we treated the larvae with different inhibitors targeting JAK/STAT3 signaling (Ruxolitinib, Baricitinib, WP1066, Stattic). However, none of these inhibitors improved the injury. Instead, we observed the opposite effect: an accelerated progression of the disease, which led to an increased edema and a higher mortality rate (Fig. EV2D–G). These results demonstrated that pathway inhibition at the tested time window is insufficient to rescue the phenotype.

### Differential expression of miRNAs in the FSGS injury model

**Figure 4:**
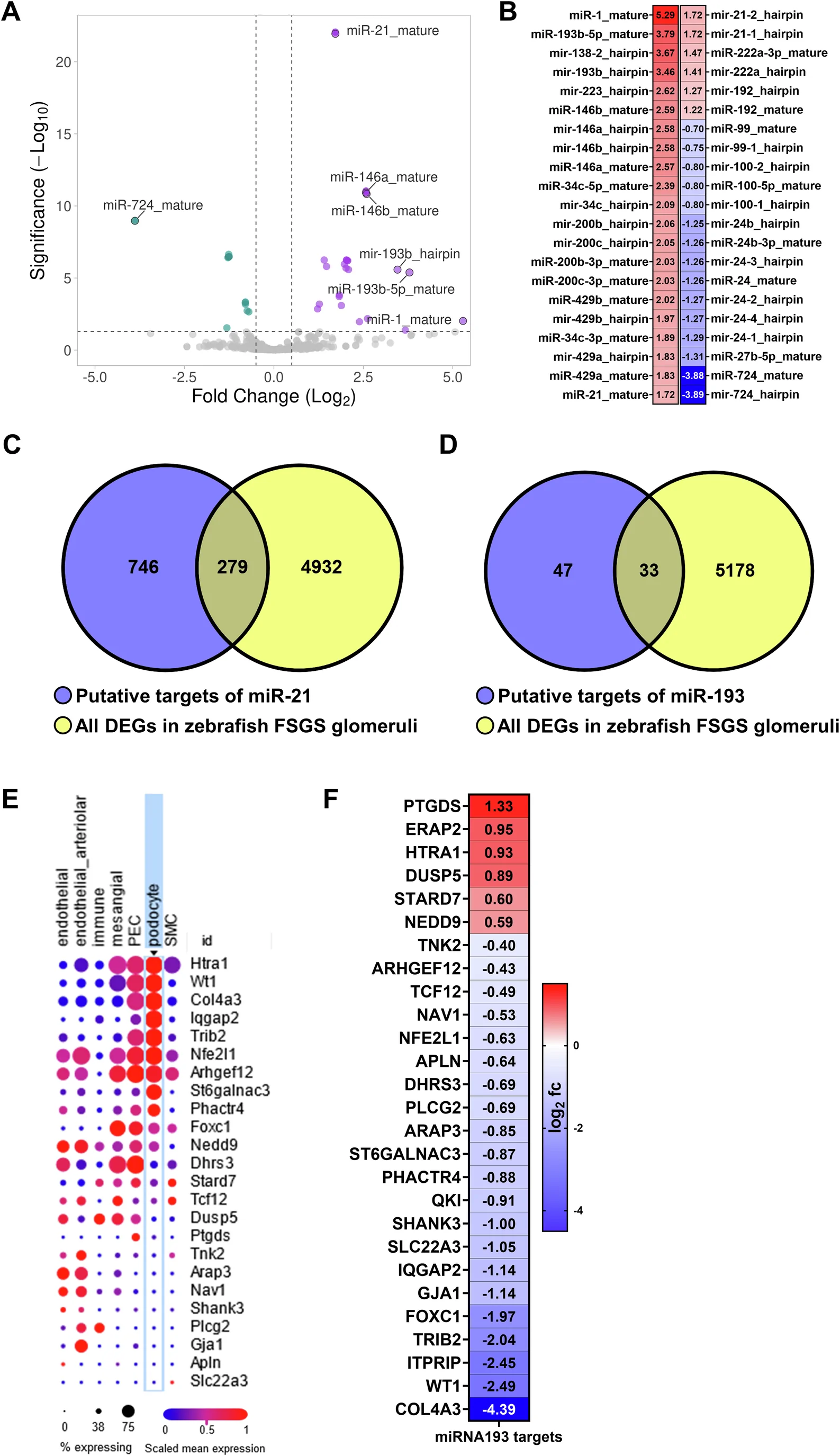
Differential expression of miRNAs in glomeruli of FSGS-induced zebrafish larvae. (**A**) Volcano plot of differential expression of miRNAs in isolated glomeruli (6 dpf, *n* = 3 independent biological replicates per condition) after podocyte-specific glomerular injury compared to control groups. Data represent miRNA expression profiles identified by RNA sequencing. Significantly upregulated (purple) and downregulated (cyan) miRNAs are highlighted (adjusted *P* value < 0.05; log2 fold change ≥0.5). (**B**) Heatmap of all significantly up- and downregulated miRNAs after FSGS induction (isolated glomeruli, 6 dpf; *n* = 3 biological replicates per condition). (**C**) Overlap of putative targets of miR-21 (taken from miRDB (Data ref: Chen and Wang, 2020) with all DEGs in zebrafish glomeruli after FSGS induction. (**D**) Overlap of experimentally validated targets of the miR-193 taken from Gebeshuber et al, 2013 with all DEGs in FSGS glomeruli. (**E**) Single-cell RNA-seq data of mouse glomerulus (Data ref: Chung et al, 2020) from all putative miR-193 targets found in the zebrafish injury model. (**F**) Heatmap of the glomerular expression patterns of miR-193 targets in NFP-treated zebrafish larvae. Data are expressed as log2 fold change, a *P* value < 0.05 was considered as significant. Red: upregulated; blue: downregulated compared to controls. Differential expression analysis was performed using DESeq2. Statistical significance was assessed using the Wald test, and *P* values were adjusted for multiple testing using the Benjamini–Hochberg method. Source data are available online for this figure.

To determine whether specific miRNAs are expressed in FSGS that regulate distinct signaling pathways, we analyzed the miRNAs expression pattern by small RNA sequencing (*n* = 3 independent biological replicates per condition, isolated zebrafish glomeruli at 6 dpf). This analysis identified 42 significantly regulated miRNAs (Fig. 4A,B). In addition to miR-21 and miR-193, two miRNAs strongly linked to FSGS and CKD, we identified miR-146, miR-34, miR-200, and miR-429 (Fig. 4A,B). These miRNAs are known to be involved in inflammation, apoptosis and fibrosis.

Furthermore, we focused our attention on miR-21 and miR-193. Target gene identification was performed bioinformatically: putative targets of miR-21 were retrieved from miRDB (Data ref: Chen and Wang, 2020), and targets of miR-193 were taken from the experimentally validated list of Gebeshuber et al, 2013. We analyzed miR-21 target genes that overlapped with glomerular mRNA DEGs and found 279 potential targets (Fig. 4C), including *epb41l5, npr3* and *ptpro*. For miR-193, 33 predicted targets (Fig. 4D) were differentially expressed in the injured zebrafish glomeruli. Comparative analysis with mouse glomerular scRNA-seq data showed several target genes that are enriched in podocytes (e.g., *Htra1, Wt1, Col4a3, Trib2, Nfe2l1, Arhgef12*) (Fig. 4E). Furthermore, we found that the key miR-193-associated podocyte genes such as *wt1*, *col4a3*, and *foxc1*, were significantly downregulated in injured zebrafish glomeruli (Fig. 4F), showing the homology between fish, mice and humans.

### Alternative splicing events in zebrafish larvae and isolated glomeruli in the FSGS model

**Figure 5:**
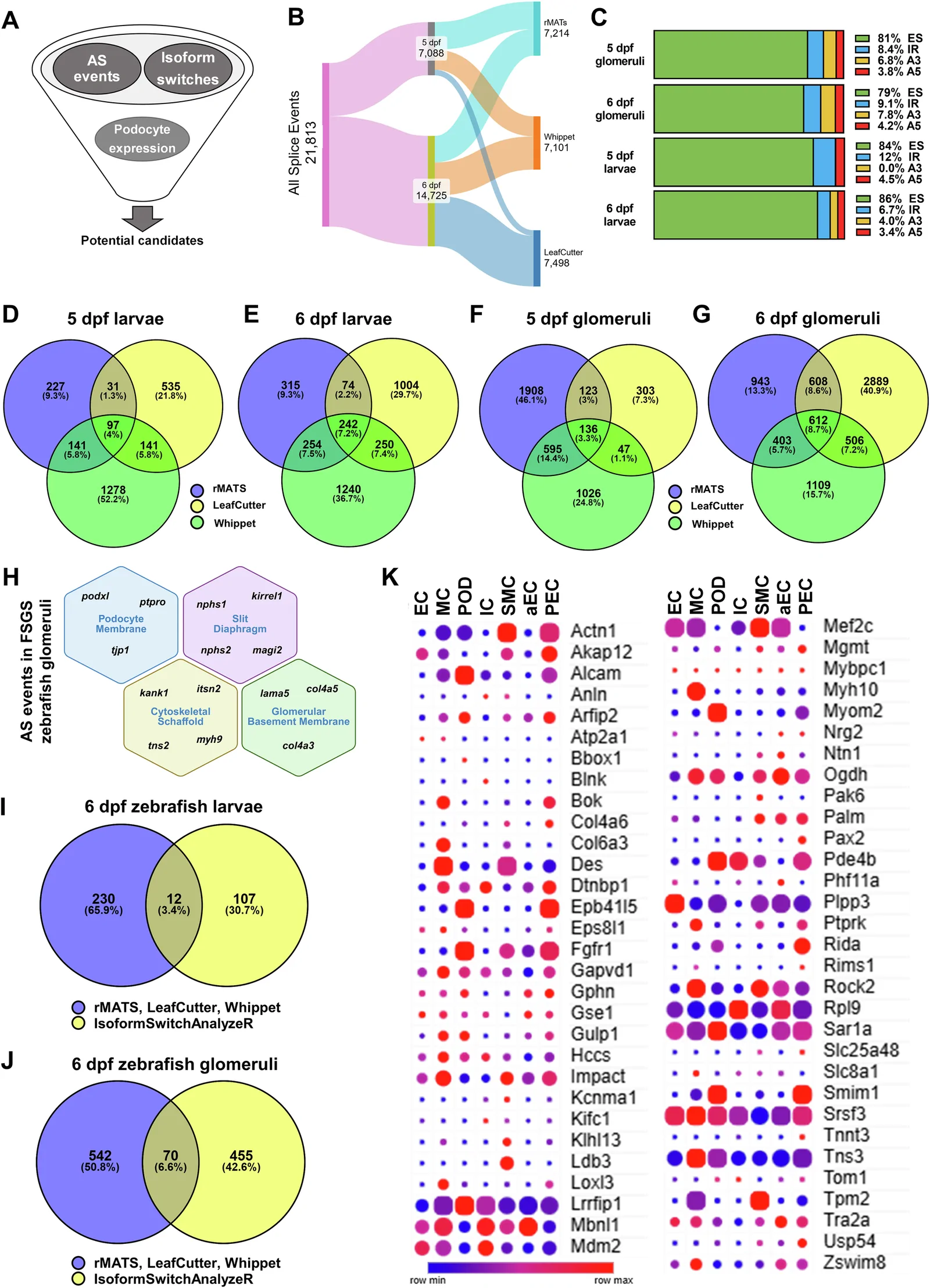
Differential alternative splicing events in glomeruli of zebrafish larvae. (**A**) Methodical workflow for the identification of potential candidate genes. Alternative splicing (AS) events were identified using rMATS, LeafCutter, and Whippet. Additionally, isoform switches were analyzed with IsoformSwitchAnalyzeR. Finally, all identified genes were screened for podocyte-specific expression. (**B**) Sankey’s diagram illustrates the flow of alternative splicing events through three tools (rMATS, LeafCutter, Whippet) for isolated 5 dpf and 6 dpf glomeruli. (**C**) Percentage distribution of the types of AS events found in rMATS: ES (exon skipping); IR (intron retention); A5SS (Alternative 5′ splice site); A3SS (Alternative 3′ splice site). (**D**–**G**) Venn diagram depicts the overlap of genes that showed a significant splice event at 5 dpf (24 h after treatment) and 6 dpf (after 48 h treatment) in rMATS, leafcutter and Whippet in larvae (**D**, **E**) and isolated glomeruli (**F**, **G**). (**H**) Shown are primarily podocyte-specific genes with AS events detected in glomeruli upon FSGS induction, which are also known to play a role in glomerular disease. (**I**) The overlap shown in (**E**) was integrated with isoform switch analysis of whole larvae at 6 dpf. (**J**) The intersecting genes from (**G**) were cross-referenced with isoform switching data from isolated glomeruli at 6 dpf. (**K**) Single-cell RNA-Seq expression data of mouse glomerulus (Data ref: Chung et al, 2020) of all alternatively spliced genes found parallel in rMATS, LeafCutter, Whippet and IsoformSwitchAnalyzeR. EC glomerular endothelial cells, MC mesangial cells, POD podocytes, IC immune cells, SMC smooth muscle cells, aEC arteriolar endothelial cells, PEC parietal epithelial cells. Source data are available online for this figure.

To determine whether podocyte injury is accompanied by an alteration of the alternative splicing (AS), we performed splicing analysis using three complementary tools (rMATS, LeafCutter, Whippet) and assessed isoform switching with IsoformSwitchAnalyzeR (Fig. 5A,B). Splicing analysis showed that exon skipping was the predominant splicing event (~80%), followed by intron retention and alternative splice-site usage (Fig. 5C).

Comparison of AS events across all used tools indicated a time-dependent increase in genes with AS. We identified 97 AS genes in whole larvae (5 dpf) and 136 in isolated glomeruli (Fig. 5D,F). AS events increased to 242 genes in larvae and 612 in glomeruli at 6 dpf (Fig. 5E,G). While most AS events changed during the two developmental time points, a subset of genes exhibited the same AS at both time points (Appendix Figs. S4 and S5).

Notably, many alternatively spliced genes were podocyte-enriched genes and are linked to the glomerular filtration barrier, such as slit diaphragm proteins (*nphs1, nphs2*, *kirrel1, magi2*), cytoskeletal regulators (*tns2, myh9, itsn2, kank1*), membrane/polarity genes (*podxl, ptpro, tjp1*), and glomerular basement membrane proteins (*lama5, col4a3, col4a5*) (Fig. 5H).

### Isoform switch and nuclear expression of SRSF3 in zebrafish and human glomeruli after FSGS onset

By using IsoformSwitchAnalyzeR, we identified 12 genes undergoing significant isoform switches in whole larvae and 70 genes in glomeruli after FSGS induction (Fig. 5I,J). Screening for podocyte-enriched expression identified three candidates that met the selection criteria detection across all tools, a significant isoform switch and podocyte enrichment: *epb41l5*, *fgfr1a*, and the splicing factor *srsf3a* (Fig. 5K; Appendix Fig. S6).

**Figure 6:**
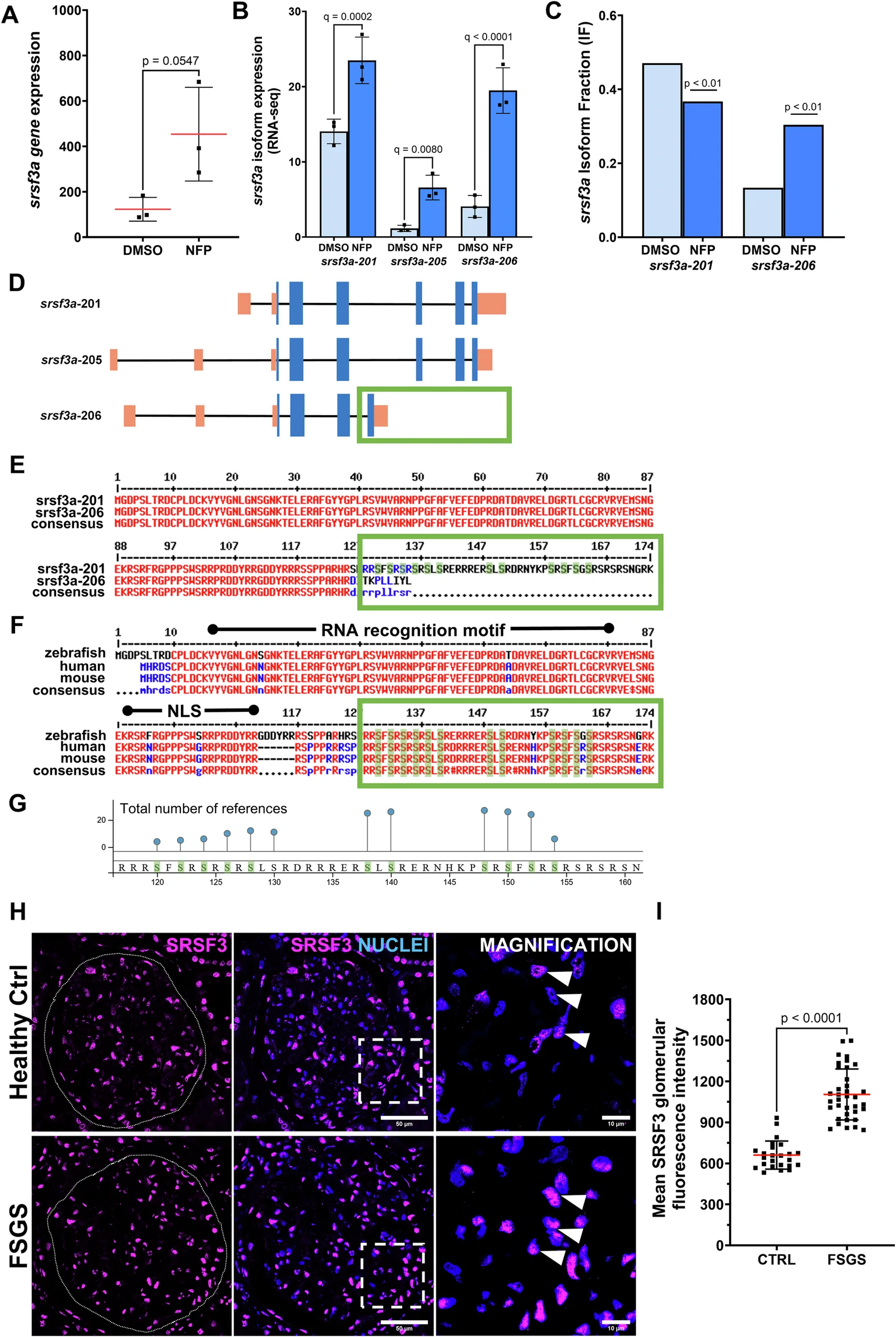
Transcriptomic characterization of *srsf3a* isoforms, phosphorylation sites, and glomerular SRSF3 expression in human FSGS. (**A**–**C**) Gene expression (**A**), isoform expression (**B**), and isoform fraction (**C**) of *srsf3a* in isolated zebrafish glomeruli in DMSO and NFP-treated samples at 6 dpf (*n* = 3 independent biological replicates per condition). “Isoform expression” refers to DESeq2-normalized absolute read counts per isoform; “isoform fraction” (IF) denotes the relative proportion of each isoform as a fraction of total gene expression, capturing isoform usage shifts independently of overall expression level changes. Gene annotations were based on Ensembl release 113 (October 2024). (**D**) Overview of the *srsf3a* isoforms found in zebrafish larvae and glomeruli. Blue: coding exons; orange: untranslated regions (UTRs). (**E**) Alignment of the protein sequences of srsf3a-201 and srsf3a-206 isoform. (**F**) Alignment of the SRSF3 protein sequences between zebrafish, human and mouse. Known phosphorylation sites are marked in green. (**G**) Published and described phosphorylation sites of Srsf3 from PhosphoSitePlus (Hornbeck et al, 2015). (**H**) Immunofluorescence staining for SRSF3 (magenta) and nuclei (blue) in control kidney tissue (Healthy Ctrl) and in patients with focal segmental glomerulosclerosis (FSGS). Low-magnification overview (scale bar 50 µm) and high-magnification insets (scale bar 10 µm) are shown; synaptopodin co-staining (see Fig. EV3D) confirms podocyte identity of SRSF3-positive nuclei. The glomeruli are indicated by a white dashed line. Podocytes are indicated by arrowheads. (**I**) Mean SRSF3 fluorescence intensity in DAPI-positive glomerular nuclei of human control and FSGS samples. Each dot represents the value of one individual glomerulus (Ctrl: *n* = 24; FSGS: *n* = 36). Statistical significance was assessed using unpaired *t* test (**A**), one-way ANOVA followed by Benjamini–Hochberg correction (**B**) and Welch’s *t* test (**I**); *q* values and *P* values are indicated; mean ± SD. Source data are available online for this figure.

**Figure EV3:**
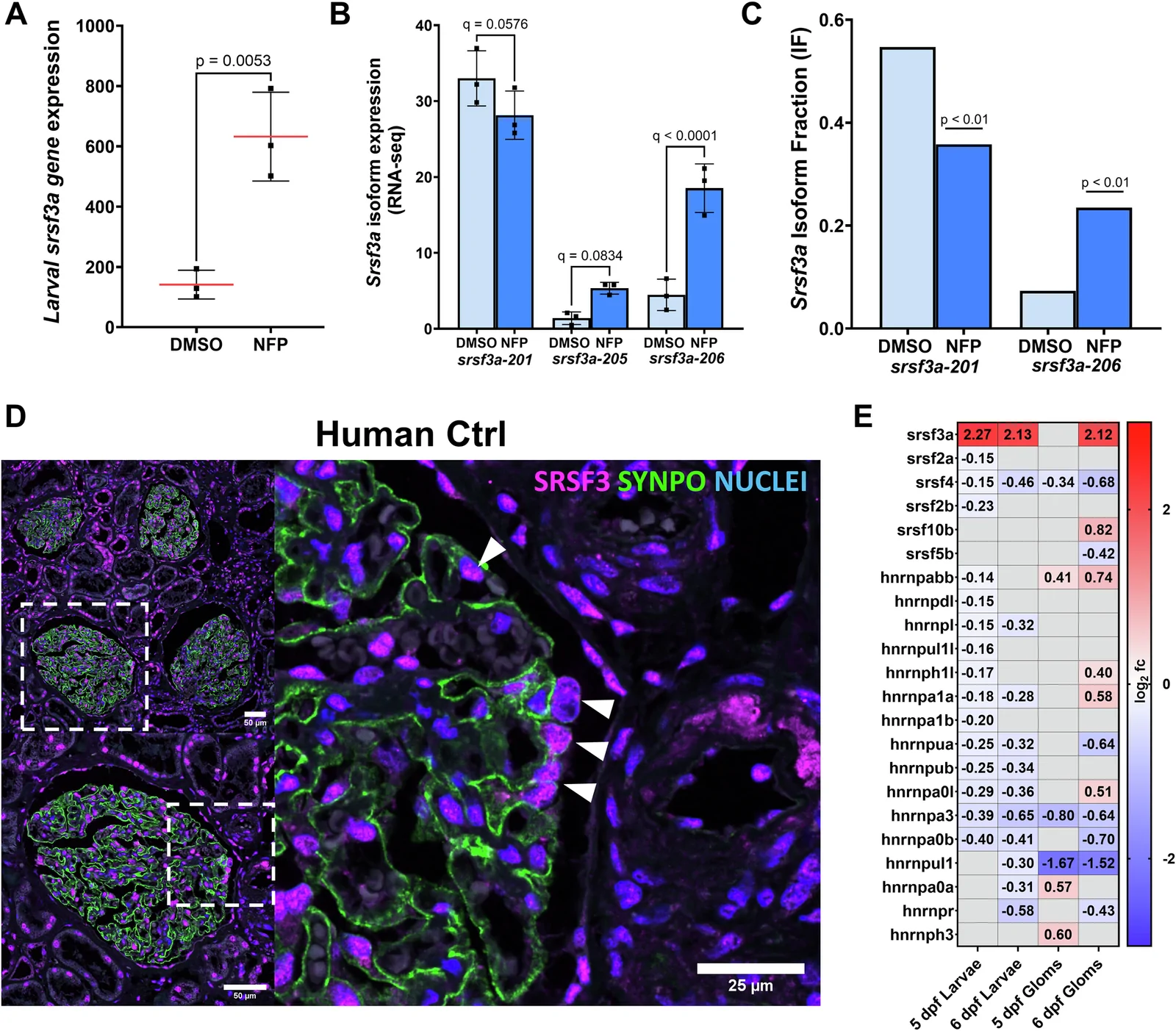
SRSF3 isoform regulation in zebrafish larvae and nuclear localization in human kidney. This figure supplements Fig. 6 of the main text by providing srsf3a isoform data from whole larvae (**A**–**C**) alongside the glomerular data shown in Fig. 6A–C, and additional human validation data. (**A**–**C**) Gene expression (**A**), isoform expression (**B**; DESeq2-normalized counts), and isoform fraction (**C**; proportion of each isoform relative to total *srsf3a* expression) of *srsf3a* in zebrafish whole larvae at 6 dpf (NFP vs. DMSO; *n* = 3 biological replicates per condition; mean ± SD; unpaired *t* test). All gene annotations: Ensembl release 113. (**D**) Confocal images of human control kidney sections showing nuclear SRSF3 immunoreactivity (magenta) within glomerular cells; synaptopodin (green) labels podocytes; nuclei stained with DAPI (blue). Arrowheads indicate podocytes. Scale bars: 50 µm and 25 µm. SRSF3 signal is detected in multiple glomerular cell types; podocyte co-localization is confirmed by synaptopodin overlap (arrowheads in magnification inset). (**E**) Heatmaps of gene expression levels of all SRSF-family and hnRNP-family members in zebrafish larvae and glomeruli (log2 fold change, NFP vs. DMSO; *P* < 0.05, DESeq2). Red: upregulated; blue: downregulated. Statistical significance was assessed using unpaired *t* test (**A**), and one-way ANOVA followed by Benjamini–Hochberg correction (**B**); *q* values and *P* values are indicated; mean ± SD.

*Srsf3a* (Serine and arginine-rich splicing factor 3a) is a protein that primarily binds mRNA and plays a key role in splicing processes, but it is also involved in RNA stability, miRNA biogenesis and many different cells signaling and differentiation pathways. The ortholog in humans is *SRSF3* which is localized on chromosome 6. Our study showed a strong increase in gene and transcript expression of *srsf3a* in larva glomeruli and whole larvae after disease onset (Figs. 6A,B and EV3A,B). However, isoform 206 increased more strongly than isoform 201, resulting in a shift in relative isoform expression toward isoform 206 (Figs. 6C and  EV3C).

Both Srsf3 isoforms still have the N-terminal RNA recognition motif and a nuclear localization signal, showing a preserved nuclear RNA binding motif (Fig. 6D–F). In contrast to isoform 201, isoform 206 lacks a serine-rich C-terminal region with 38 amino acids (Fig. 6D,E), which is also conserved across zebrafish, mouse, and human and contains twelve phosphorylation sites (Fig. 6F,G). It is described that due to an upregulation of *srsf3*, the isoform 206 could be activated in an autoregulatory way to counteract the full-length isoform function. In human glomeruli, we detected a strong expression of SRSF3 in the nuclei, particularly in synaptopodin-positive podocytes, which was upregulated in biopsies of FSGS patients (Figs. 6H,I and EV3D). Beside Srsf3, additional serine/arginine-rich splicing factors (SRs) showed altered expression (e.g. downregulation of *srsf2a/b* and *srsf4*) in zebrafish larvae, and also heterogeneous nuclear ribonucleoproteins (hnRNPs) were consistently downregulated (Fig. EV3E), indicating a shift in the splicing regulator protein family.

### Alternative splicing of *epb41l5* and *fgfr1a* in injured glomeruli

**Figure 7:**
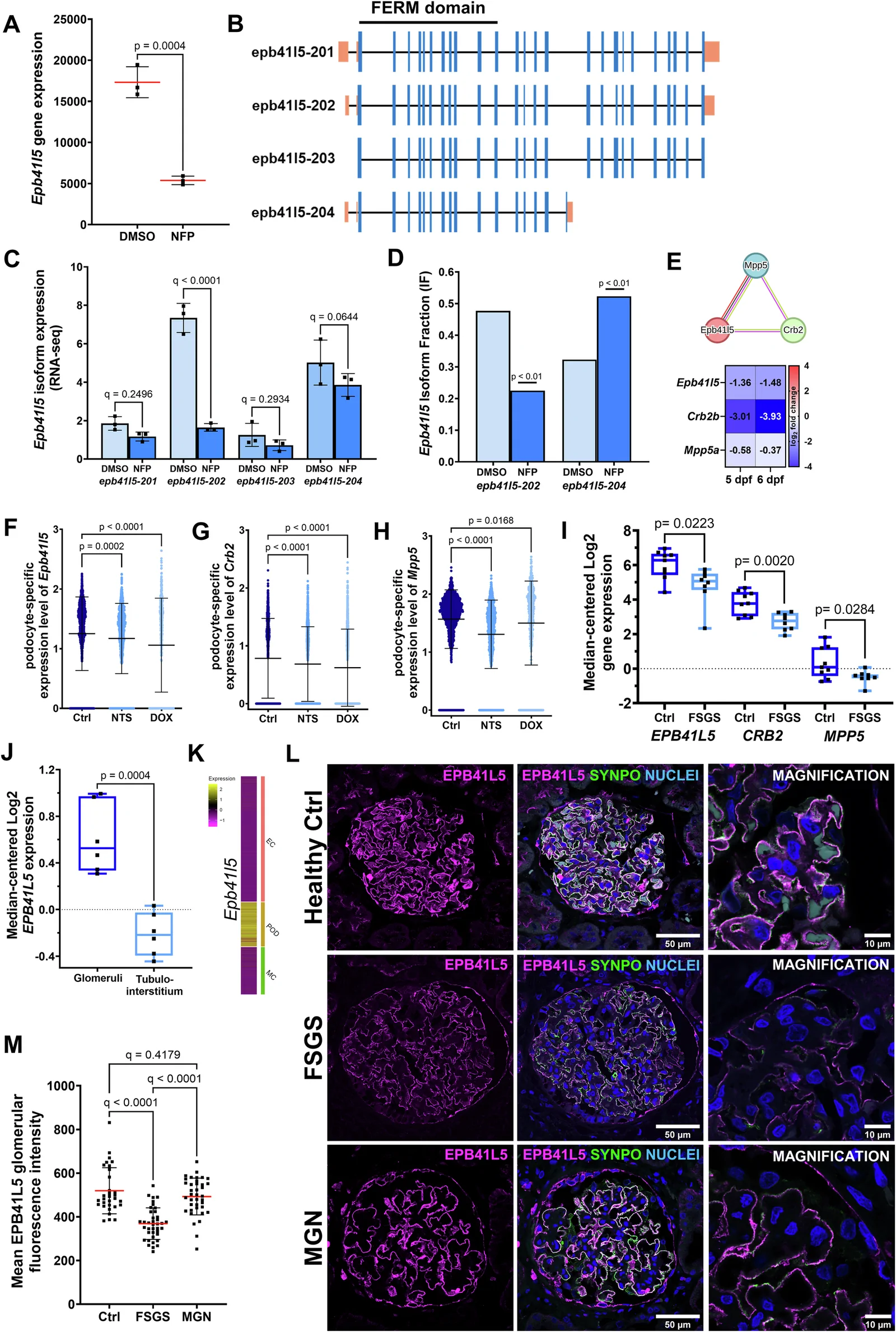
Alternative splicing and regulation of *epb41l5* in injured glomeruli. (**A**) Gene expression of *epb41l5* in glomeruli treated with DMSO and NFP at 6 dpf; (*n* = 3 biological replicates) (**B**) Overview of all *epb41l5* isoforms found in zebrafish glomeruli. FERM domain is highlighted. Blue: coding exons; orange: untranslated regions (UTRs). (**C**) Expression of *epb41l5* isoforms based on DESeq2 normalized gene counts from RNA-Seq at 6 dpf. (**D**) *Epb41l5* isoform fraction based on IsoformSwitchAnalyzer data at 6 dpf. (**E**) STRING protein-protein interaction network showing strong correlated interactions of Epb41l5 with Crb2 and Mpp5. Heatmaps of gene expression levels of *epb41l5* and his interaction partner CRUMBS2 (*crb2a*) and PALS1 (*mpp5a*) in isolated glomeruli of zebrafish larvae after FSGS induction at 5- and 6 dpf. Data are represented as log2 fold change, a *P* value < 0.05 was considered as significant. Red: upregulated; blue: downregulated compared to controls. (**F**–**H**) Single-cell RNA-Seq data shown podocyte-specific gene expression of *Epb41l5* (**F**), *Crb2* (**G**) and *Mpp5* (**H**) in mouse with nephrotoxic serum nephritis (NTS) at day 5 and doxorubicin (DOX) compared to the control (Ctrl) (Ctrl *n* = 2018; NTS *n* = 1434; DOX *n* = 504) (Chung et al, 2020)). (**I**) The mRNA expression level of *EPB41L5*, *CRB2* and *MPP5* in renal glomeruli from human with focal segmental glomerulosclerosis (FSGS) compared to human control (Ctrl). Data were taken from Nephroseq Research Edition (www.nephroseq.org) “Hodgin FSGS Glom Dataset” (Data ref: Hodgin et al, 2010 and are represented as log2 fold change, a *q* value < 0.05 was considered as significant (Ctrl: *n* = 9; FSGS: *n* = 8). Box plots show the median (center line), 25th and 75th percentiles (box boundaries), and minimum and maximum values (whiskers). (**J**) Microarray data showed an enrichment of *EPB41L5* gene expression in glomeruli (*n* = 6) compared to tubulointerstitium (*n* = 6). Data from Nephroseq database “Lindenmeyer Normal Tissue Panel” (Data ref: Lindenmeyer et al, 2010). Box plots show the median (center line), 25th and 75th percentiles (box boundaries), and minimum and maximum values (whiskers). (**K**) Heatmap of single-cell RNA expression data of *Epb41l5* in mice glomeruli showed significant enrichment in the podocyte cluster (POD). Color code: Yellow means a high average expression; magenta: low expression. EC endothelial cells, MC mesangial cells. (**L**) Immunofluorescence staining of EPB41l5 (shown in magenta) in human biopsies (healthy control, FSGS and membranous glomerulopathy (MGN)). Synaptopodin is shown in green. Nuclei were stained with DAPI (blue). Scale bars represent 50 µm and 10 µm (magnification) respectively. (**M**) Mean EPB41L5 fluorescence intensity per glomerulus (Ctrl: *n* = 31; FSGS: *n* = 39; MGN: *n* = 38; individual glomeruli shown as dots). All gene annotations were based on Ensembl release 113 (October 2024). Statistical significance was assessed using unpaired *t* test (**A**), one-way ANOVA followed by Benjamini–Hochberg correction (**C**, **F**, **G**, **H**, **M**), and Welch’s *t* test (**I**, **J**); *q* values and *P* values are indicated; mean ± SD. Source data are available online for this figure.

EPB41L5 (erythrocyte membrane protein band 4.1 like 5), a regulator of epithelial polarity and cell adhesion, is highly expressed in zebrafish, mouse and human glomeruli (Fig. 7A,J–L). The results of the microarray analysis of microdissected human glomeruli showed also a significant glomerular enrichment of EPB41L5 (Fig. 7J,K). After glomerular injury in zebrafish larvae, *epb41l5* gene expression was significantly reduced (Fig. 7A; Appendix Fig. S7A). Moreover, isoform analysis identified four differentially abundant splice variants as well as a disease-associated switch to the isoform *epb41l5*-204 (Fig. 7B–D; Appendix Fig. S7B–D). Isoform *epb41l5*-204 is characterized by a truncation of the C-terminal part of the protein due to the loss of the last nine exons (Fig. 7B–D; Appendix Fig. S7B–D). Further, the binding partners of *epb41l5*, *crb2* and *mpp5*, were also downregulated in larval glomeruli after FSGS induction (Fig. 7E). Analysis of human FSGS data and mouse scRNA-seq data showed downregulation of *EPB41L5*, *CRB2* (crumbs cell polarity complex component 2), and *MPP5* (also known as *PALS1*; protein associated with LIN7 1) in podocytes (Fig. 7F–I). This indicates a coordinated dysregulation of polarity-associated protein networks due to a podocyte injury (Fig. 7E).

To validate the above findings, we performed quantitative immunofluorescence of human biopsies. Here we found a significant reduction of the EPB41L5 expression exclusively in glomeruli from FSGS biopsies in comparison with healthy tissue and membranous glomerulopathy (MGN) biopsies (Fig. 7L,M).

**Figure EV4:**
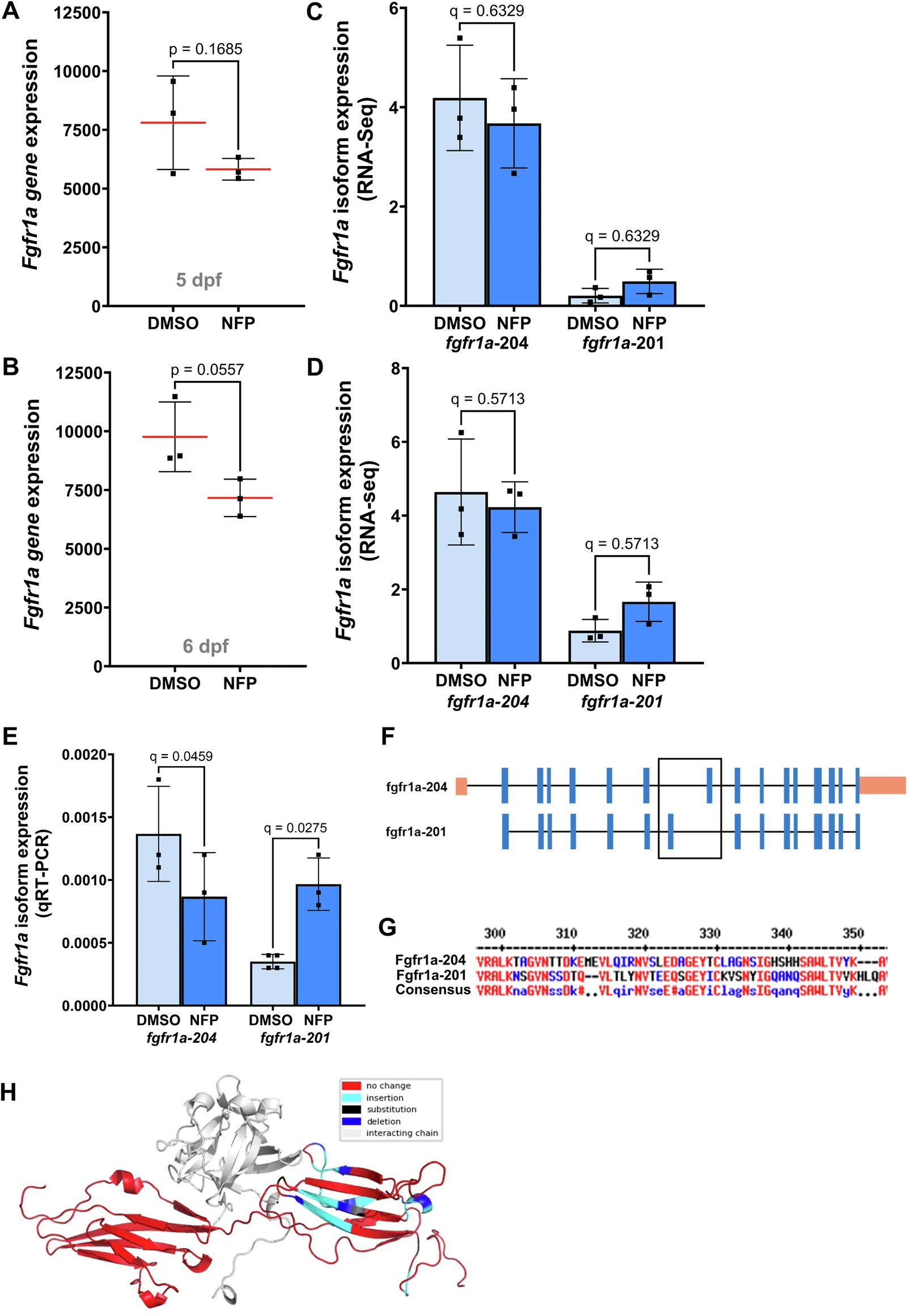
Regulation of *fgfr1a* isoforms in FSGS-injured zebrafish glomeruli. This figure documents the isoform switch in *fgfr1a*, a receptor tyrosine kinase, in FSGS-injured glomeruli, demonstrating that alternative splicing can alter signaling capacity independently of overall gene expression changes. Dataset: isolated zebrafish glomeruli, NFP vs. DMSO. (**A**, **B**) *Fgfr1a* gene expression at 5 dpf (**A**) and 6 dpf (**B**) (*n* = 3 biological replicates). (**C**, **D**) Glomerular *fgfr1a* isoform expression at 5 dpf (**C**) and 6 dpf (**D**) (*n* = 3 biological replicates) (DESeq2-normalized counts). (**E**) Validation of *fgfr1a* isoform expression by qRT-PCR (normalized to DMSO glomeruli and Gapdh; *n* = 3 or 4 biological replicates). (**F**) Schematic of *fgfr1a* isoforms (blue: coding exons; orange: UTRs). (**G**) Protein sequence alignment of the two Fgfr1a isoforms highlighting isoform-specific amino acid differences. (**H**) Structural analysis comparing Fgfr1a isoforms 204 (ENSDART00000147742) and 201 (ENSDART00000024882): red = no change; cyan = insertion; black = substitution; blue = deletion; gray = FGF2 interaction chain. The isoform switch is predicted to reduce FGF2 binding affinity. All gene annotations: Ensembl release 113. Statistical significance was assessed using unpaired *t* test (**A**, **B**), one-way ANOVA followed by Benjamini–Hochberg correction (**C**–**E**); *q* values and *P* values are indicated; mean ± SD.

In contrast to these findings, *fgfr1a* (fibroblast growth factor receptor 1a) mRNA expression, a tyrosine kinase receptor, remained unchanged after FSGS induction in zebrafish larvae (Fig. EV4A,B). However, analysis of *fgfr1a* transcripts showed that two isoforms changed their expression intensity in larval glomeruli after FSGS induction: *Fgfr1a*-204 was downregulated (−37%), whereas *fgfr1a*-201 was upregulated (+176%) (Fig. EV4C,D). After comparison of these two *Fgfr1* isoforms by the analysis tools FoldX (Delgado et al, 2019) and StructMAn (Gress et al, 2022), we found that alternative splicing alters the position of the coding exon 7 at the pre-mRNA level (Fig. EV4F). This results in isoform-specific nucleotide sequences and corresponding changes in the encoded amino acids (Fig. EV4G). These alterations affect the FGF-binding domain and are predicted to reduce the interaction with fibroblast growth factor 2 (FGF2) (Fig. EV4H).

These results clearly demonstrate that isoform switches modulate key pathways in podocytes during FSGS, and that alternative splicing can modulate signaling independently of changes in gene expression.

## Discussion

The progression of focal segmental glomerulosclerosis (FSGS) reflects profound alterations at multiple levels of RNA regulation. Using RNA-seq and cross-species analysis, our zebrafish glomerular injury model (Schindler and Endlich, 2024; Hansen et al, 2020; Schindler et al, 2023; Klawitter et al, 2024) reveals post-transcriptional changes and transcriptional dysregulation, providing a comprehensive landscape of post-transcriptional regulatory alterations during disease progression.

Our data identified specific microRNA induction and widespread alternative splicing as central features of podocyte injury and disease progression.

Consistent with previous studies in human FSGS, we observed robust downregulation of podocyte-specific genes in our zebrafish injury model, including the slit diaphragm components (such as *nphs1*, *nphs2*) (Kim et al, 2002; Mollet et al, 2009; Huber et al, 2003), cytoskeletal regulators (*actn1*, *podxl*) and podocyte-enriched transcription factors (*wt1*, *foxc1*, *mafb*) (Benetti et al, 2010; Sato et al, 2018; Usui et al, 2020; Motojima et al, 2017). These changes were associated with the activation of inflammation-associated pathways, reflecting a transition from a differentiated podocyte state toward an injury-associated phenotype.

Interestingly, pharmacological inhibition of the IL6/JAK/STAT3 pathway failed to ameliorate disease progression in the zebrafish model and instead exacerbated injury. While STAT3 activation is clearly induced in mice and human FSGS (Tao et al, 2018; Henique et al, 2017), our findings suggest that downstream post-transcriptional pathways may represent a point of limited reversibility.

The absence of rescue may reflect the inherent stringency of the NFP model, in which rapid and irreversible podocyte depletion may exceed the capacity of single-pathway inhibition. Additionally, compensatory activation of parallel inflammatory pathways (e.g., NF-κB, mTOR, TGF-β) may sustain disease progression even under pharmacological JAK/STAT3 blockade, consistent with the complexity of signaling networks reported in human and murine FSGS (Tao et al, 2018; Henique et al, 2017).

However, we show a strong overlap between zebrafish and human glomerular transcriptomes, which underscores the translational relevance of this FSGS model and supports its suitability for dissecting conserved molecular mechanisms underlying FSGS. Integration with mouse glomerular single-cell RNA-seq data from nephrotoxic serum nephritis and doxorubicin models (Chung et al, 2020) further corroborated these findings, demonstrating cross-species conservation of key injury-associated transcriptional and splicing changes across zebrafish, mouse, and human.

Beyond transcriptional changes, our analyses revealed significant changes in the expression of miRNAs previously implicated in glomerular disease, most notably miR-21 and miR-193 (Lange et al, 2019; Gebeshuber et al, 2013; Bharati et al, 2023; Wang et al, 2021). Both miRNAs have been shown to promote podocyte injury and fibrotic remodeling, and our data extend these findings by demonstrating coordinated repression of podocyte-enriched target genes in vivo. However, miRNA-mediated repression contributes to the loss of podocyte identity, but does not fully account for the extensive molecular remodeling observed during later stages of disease progression.

A central and novel finding of our study is the widespread and characteristic change of alternative splicing in injured glomeruli. Alternative splicing events increased markedly over time and preferentially affected podocyte-enriched genes involved in slit diaphragm integrity, in cytoskeletal organization, and in glomerular basement membrane composition. Our analysis revealed differential exon usage in key podocyte-specific genes, including *nphs1*, *nphs2*, *podxl*, *ptpro*, and *magi2*. Previous studies have provided evidence that these genes are subject to alternative splicing (Aguiar et al, 1999; Holthöfer et al, 1999; Beltran et al, 2003; Balbas et al, 2014; Fu et al, 2022; Luimula et al, 2000; Relle et al, 2011). This underlines that change in alternative splicing directly targets the structural and signaling architecture of the filtration barrier, thereby amplifying podocyte dysfunction beyond transcriptional control alone.

Here, we identified a disease-associated isoform switch of the splicing factor SRSF3 as a potential regulatory hub underlying altered splicing in podocytes. Isoform analysis showed a pronounced shift toward an isoform lacking a conserved serine-rich C-terminal domain, the isoform 206. This domain contains multiple serine phosphorylation sites that regulate nucleocytoplasmic shuttling and mRNA export of SR proteins (Brooks et al, 2026; Cazalla et al, 2002; Botti et al, 2017). However, the loss of this regulatory region is likely the effect of the *Poison*-Exon 4 which contains a stop codon and therefore reduces Srsf3 by autoregulation as it was described previously (Jia and Zheng, 2023; Guo et al, 2018). This means that Srsf3 is a protein that regulates its own expression by alternative splicing of its pre-mRNA. Splicing-corrective strategies targeting this isoform switch, for example, via splice-switching antisense oligonucleotides or through modulation of the kinase/phosphatase networks controlling splicing factor phosphorylation, represent a promising and largely unexplored therapeutic direction in podocyte disease.

In addition to srsf3a, we observed the regulation of additional serine/arginine-rich splicing factor 4 (*srsf4*) and the heterogeneous nuclear ribonucleoproteins (*hnrnpa3, hnrnpul1*), indicating a broader change of splicing homeostasis after disease onset.

The functional relevance of alternative splicing is further supported by disease-specific isoform switches of key regulators of podocytes, as it was found for EPB41L5. EPB41L5 is a critical mediator of epithelial polarity and adhesion as we and others have described also for podocytes (Schell et al, 2017; Jensen and Westerfield, 2004; Kramer-Zucker et al, 2005; Maier et al, 2021). Here we found a reduced expression of the protein together with an isoform switch. Moreover, we found also a downregulation of its interaction partners CRB2 and MPP5. CRB2 is well known to play a central role for podocyte foot process morphology and the size selectivity of the filtration barrier (Möller-Kerutt et al, 2021; Tanoue et al, 2021) and MPP5 is a central component of this Crumbs polarity complex (Gosens et al, 2007; Pieczynski and Margolis, 2011). A disruption of this complex leads to proteinuria and podocyte foot process effacement as it is the hallmark for FSGS.

In summary, our work provides a comprehensive, glomerulus-resolved map of post-transcriptional regulation in podocyte injury and a conceptual framework linking podocyte injury to RNA regulation, particularly alternative splicing. Importantly, our findings identified alternative splicing as a key regulatory mechanism which is associated with the progression of glomerular disease in zebrafish, mouse and human models.

## Methods

**Table:** Reagents and tools table

Reagent or resource	Source	Identifier
**Antibodies**		
Rabbit anti-EPB41L5	Sigma-Aldrich	Cat# HPA037564
Rabbit anti-PLCE1	Proteintech	Cat# 55463-1-AP
Rabbit anti-SRSF3	Proteintech	Cat# 10916-1-AP
Guinea pig anti-Nephrin	Progen	Cat# GP-N2
Anti-Synaptopodin	Progen	Cat# 61094
Cy2-conjugated secondary antibody	Jackson ImmunoResearch	Code: 715-545-150 // Code:111-225-144
Cy3-conjugated secondary antibody	Jackson ImmunoResearch	Code: 115-165-003 // Code: 111-165-144
**Chemicals, peptides, and drugs**		
Nifurpirinol (NFP)	Sigma-Aldrich	32439
Dimethyl sulfoxide (DMSO)	Sigma-Aldrich	D8418
Belinostat (positive control)	MedChemExpress	Cat# HY-10225
Stattic (STAT3 inhibitor)	MedChemExpress	Cat# HY-13818
Ruxolitinib (JAK1/2 inhibitor)	MedChemExpress	Cat# HY-50856
Baricitinib (JAK1/2 inhibitor)	MedChemExpress	Cat# HY-15315
WP1066 (STAT3 inhibitor)	MedChemExpress	Cat# HY-15312
Tri-Reagent	Sigma-Aldrich	T9424
DAPI	Sigma-Aldrich	CAS No. 28718-90-3
Mowiol mounting medium	Carl Roth	Art. No. 0713.1
iTaq Universal SYBR Green Supermix	Bio-Rad	#L001752B
**Critical commercial assays**		
miRNeasy Micro Kit	Qiagen	N/A
RNA 6000 Pico Kit	Agilent Technologies	N/A
QuantiTect Reverse Transcription Kit	Qiagen	N/A
RNase-Free DNase I Kit	Norgen Biotek Corp.	N/A
RNA Clean-Up and Concentration Kit	Norgen Biotek Corp.	N/A
TruSeq Stranded mRNA Library Prep Kit	Illumina	N/A
**Deposited data**		
RNA-seq raw and preprocessed data (this study)	NCBI GEO	GSE301334
Mouse glomerular scRNA-seq (Chung et al, 2020)	BroadSingleCell Portal	GSE146912
Human FSGS glomerular expression (Hodgin et al, 2010)	Nephroseq	Hodgin FSGS Glom Dataset
Human normal tissue expression (Lindenmeyer et al, 2010)	Nephroseq	Lindenmeyer Normal Tissue Panel
Mouse kidney scRNA-seq (Ransick et al, 2019)	Kidney Cell Explorer	GSE129798
**Experimental models: organisms/strains**		
Zebrafish: Tg(nphs2:GAL4-VP16); Tg(UAS:Eco.nfsB-mCherry)	This study/Klawitter et al, 2024	https://doi.org/10.1152/ajprenal.00116.2024
**Software and algorithms**		
STAR v2.7.5c (RNA-seq alignment)	Dobin et al, 2013	https://github.com/alexdobin/STAR
featureCounts/Subread v2.0.0	Liao et al, 2014	http://subread.sourceforge.net
DESeq2 v1.38.3	Love et al, 2014	Bioconductor
Salmon v1.9.0	Patro et al, 2017	https://combine-lab.github.io/salmon
IsoformSwitchAnalyzeR v1.20.0	Vitting-Seerup and Sandelin, 2019	https://www.bioconductor.org/packages/release/bioc/html/IsoformSwitchAnalyzeR.html
rMATS v4.1.2	Shen et al, 2014	http://rnaseq-mats.sourceforge.net
LeafCutter v0.2.9	Li et al, 2018	https://github.com/davidaknowles/leafcutter
Whippet v0.11.1 (Julia v1.6.6)	Sterne-Weiler et al, 2018	https://github.com/timbitz/Whippet.jl
EnhancedVolcano (R package)	Kevin Blighe, 2018	Bioconductor
clusterProfiler v4.0	Wu et al, 2021	Bioconductor
Seurat v5.0.1	Hao et al,	https://satijalab.org/seurat
FoldX v5.0	Delgado et al, 2019	http://foldxsuite.crg.eu
StructMAn	Gress et al, 2022	https://github.com/kalininalab/d-StructMAn
Fiji/ImageJ	Schindelin et al, 2012	https://fiji.sc
StarDist plugin (Fiji)	N/A	https://github.com/stardist/stardist
GraphPad Prism v10	GraphPad Software	https://www.graphpad.com
R v4.2.0	R Core Team	https://www.r-project.org
**Equipment**		
Fluorescence stereomicroscope SMZ 18	Nikon	N/A
Bio-Rad iQ5 Real-Time PCR System	Bio-Rad	N/A
Agilent Bioanalyzer 2100	Agilent Technologies	N/A
Tape Station 4200	Agilent Technologies	N/A
NovaSeq 6000, S1 FlowCell (2×100 bp PE)	Illumina	N/A
Olympus FV3000 Confocal Microscope	Olympus	N/A
Extrafine precision tweezers (DUMONT)	DUMONT	Ref: 0208-5SPSF-PS
**Databases**		
miRDB (miRNA target prediction)	Chen and Wang, 2020	https://mirdb.org
PhosphoSitePlus	Hornbeck et al, 2015	https://www.phosphosite.org
Ensembl Genome Browser (release 104 / 113)	EMBL-EBI	https://www.ensembl.org
Zebrafish reference genome GRCz11	Genome Reference Consortium	Ensembl release 104
STRING protein interaction database	Szklarczyk et al	https://string-db.org

*N/A* not applicable/not available from manufacturer catalogue.

### Zebrafish strains and husbandry

Zebrafish were handled and maintained at 28°C under standard conditions as previously described (Schindler et al, 2020), with a 14 h light/10 h dark cycle. The stages are indicated in days post-fertilization (dpf). Embryos and larvae were raised in E3 medium and staged according to days post fertilization (dpf). The experiments were carried out from 4 to 6 dpf. For this work, the zebrafish Tg(nphs2:GAL4-VP16); Tg(UAS:Eco.nfsB-mCherry) strain was used. Larvae express the bacterial enzyme nitroreductase and the fluorescent protein mCherry exclusively in podocytes. All experiments were conducted in accordance with the German animal protection law, were overseen and approved by the “Landesamt für Landwirtschaft, Lebensmittelsicherheit und Fischerei, Rostock” (LALLF M-V) of the federal state of Mecklenburg-Western Pomerania.

### Experimental design of the FSGS zebrafish injury model

Larvae at 4 dpf were first selected for a strong homogeneous mCherry using a fluorescence stereomicroscope (SMZ 18 Nikon, Düsseldorf, Germany). FSGS was induced by the treatment of the zebrafish larvae with 50 nM Nifurpirinol (Sigma-Aldrich, St. Louis, MO, USA) dissolved in 0.1% dimethyl sulfoxide (DMSO, Sigma-Aldrich) in E3 medium. 0.1% DMSO was used for control vehicles as described previously (Klawitter et al, 2024). A treatment period of 24 h was maintained for all experiments. Larvae and isolated glomeruli were collected at 24- and 48-h post-treatment, corresponding to 5 and 6 dpf, respectively. Treatment with 50 nM Nifurpirinol resulted in the development of periocular and abdominal edema in the transgenic larvae. Furthermore, treatment induced podocyte damage, as indicated by a reduction in mCherry fluorescence and its subsequent accumulation in the proximal tubule (Appendix Fig. S1). At the developmental stages used (4–6 dpf), zebrafish larvae are sexually undifferentiated, and sex therefore does not influence the results.

For edema assessment, the larvae were divided into six groups of 50 larvae each: (a) 0.2% DMSO as control; (b) 50 nM NFP; (c) 50 nM NFP co-treated with 100 µM Belinostat (positive control); (d) 50 nM NFP co-treated with 1 µM of the inhibitor; (e) 50 nM NFP co-treated with 10 µM of the inhibitor; and (f) 50 nM NFP co-treated with 100 µM of the inhibitor. The following inhibitors were used: Stattic (HY-13818), Ruxolitinib (HY-50856), Baricitinib (HY-15315), and WP1066 (HY-15312). Only in the case of Baricitinib, the highest dose was lowered to 50 µM. Treatments were stopped after 48 h. Edema formation was evaluated at 6, 7, and 8 dpf. No samples, animals, or data points were excluded from the analyses. All collected data were included in the statistical analyses, and no data were omitted due to attrition or intentional exclusion.

### Larvae collection and glomeruli isolation

NFP- and DMSO-treated larvae at 5 and 6 dpf were dissolved in 1 ml Tri-Reagent (Sigma-Aldrich) according to manufacturer’s instructions and homogenized with ceramic beads (Carl Roth, Karlsruhe, Germany) at 5 m/s for 20 s. Glomeruli of NFP-treated and untreated larvae were isolated manually at 5 and 6 dpf using two extra-fine precision tweezers (DUMONT, Montignez, Switzerland) and a fluorescence stereomicroscope (SMZ 18 Nikon, Tokyo, Japan). Pools of 10 glomeruli were collected and centrifuged at 1000×*g* for 5 min at 4 °C. Subsequently, the supernatant was discarded and 50 µl of Tri-Reagent (Sigma-Aldrich) were added. Whole larvae and isolated glomeruli were used for the total RNA-Seq and AS analyses.

### RNA extraction, cDNA synthesis and qRT-PCR

For total RNA, samples were processed in Tri-Reagent (Sigma-Aldrich) according to manufacturer’s instructions. For cDNA synthesis, 1 µg of the isolated total RNA was transcribed using the QuantiTect Reverse Transcription Kit (Qiagen, Hilden, Germany). The quantitative real-time PCR (qRT-PCR) analysis was performed on a QuantStudio™ 5 PCR System (Thermo Fisher Scientific™) using the iTaq Universal SYBR Green Supermix (Bio-Rad) with *ef1a* as reference gene. Relative quantification of the mRNA levels was done by the efficiency corrected calculation model by Pfaffl (Pfaffl, 2001) and values are shown with standard deviations (SD) or standard error of the mean (SEM) from at least three biological replicates. Used primers are summarized in Supplemental Table 1.

### miRNA isolation and sequencing

MicroRNAs were isolated using the miRNeasy Micro Kit (Qiagen), which is specifically optimized for low-input samples. RNA concentration and integrity were evaluated using the Agilent Bioanalyzer system with the RNA 6000 Pico Kit (Agilent Technologies, Waldbronn, Germany). Sequencing of miRNAs was performed by GenXPro (Frankfurt am Main, Germany) and combined with the mRNA technique MACE (Massive Analysis of cDNA Ends) methodology, which enables digital expression profiling with high sensitivity and accuracy. Putative target genes of miR-21 were retrieved from miRDB (Chen and Wang, 2020; www.mirdb.org). Target genes of miR-193 were taken from the experimentally validated list published by Gebeshuber et al, 2013. In both cases, target identification was exclusively bioinformatic; no direct experimental validation of individual miRNA-target interactions was performed in this study.

### RNA-Seq and transcriptome and alternative splicing analysis

Samples were processed in Tri-Reagent (Sigma-Aldrich) according to the manufacturer’s instructions, followed by DNA-digestion steps using the RNase-Free DNase I Kit and RNA Clean-Up and Concentration kit according to manufacturer’s instructions (both Norgen Biotek Corp., Thorold, ON, Canada). Briefly, after quality assessment using a Tape Station 4200 (Agilent, Santa Clara, CA, USA), a PolyA library was prepared based on 500 ng total RNA per sample using the TruSeq stranded mRNA kit (Illumina, San Diego, CA, USA). Subsequently, for sequencing 200 pM of the final library were loaded on a NovaSeq 6000 System, S1 FlowCell (2 ×100 bp pair-end reads). A sequencing depth of ~200 Mio clusters per sample was obtained and recorded at the Competence Centre for Genomic Analysis in Kiel. RNA-Seq raw reads for NFP-treated zebrafish larvae and isolated glomeruli after both 5 and 6 dpf and controls were mapped to the zebrafish reference genome GRCz11 (Genome Reference Consortium Zebrafish Build 11) with the genome annotation from Ensembl version 104 using STAR version 2.7.5c (Dobin et al, 2013). We used the default parameters with a 2-pass mode recommended for AS analysis. Gene read counts were obtained by the featureCounts tool included in Subread 2.0.0 (Liao et al, 2014).

For the transcriptome analysis, all samples that passed RNA quality control and sequencing quality assessment were included in the study. No samples or data points were excluded from the downstream analyses. Therefore, no pre-established exclusion criteria were applied. Samples were assigned to experimental groups according to the study design (NFP-treated versus control). Larvae were randomly assigned to the experimental groups. Analyses were not conducted in a blinded manner. Normalization and differential expression analysis was performed using DESeq2 version 1.38.3 (Love et al, 2014) using the Wald test, and *P* values were adjusted for multiple testing using the Benjamini–Hochberg method. Adjusted *P* values < 0.05 were considered statistically significant. We visualized the differentially expressed genes using the EnhancedVolcano R package (Kevin Blighe, 2018). The heatmaps of differentially expressed genes were visualized with seaborn.clustermap function. They include on the Y-axis a dendrogram of the hierarchical clustering with a Euclidean distance of normalized and z-score standardized gene expression values in the different samples. The gene enrichment analysis was performed using Gene Set Enrichment Analysis (GSEA) with Gene Ontology terms (Subramanian et al, 2005). The dotplots of the overrepresented categories from the GO biological processes were created using the library clusterProfiler (Wu et al, 2021).

For isoform switch analysis, we prepared transcript counts using salmon version 1.9.0 (Patro et al, 2017) with the transcript annotation based on Ensembl version 104 and ran the R package IsoformSwitchAnalyzeR (Vitting-Seerup and Sandelin, 2019) version 1.20.0 with the DESeq2 algorithm. For alternative splicing analysis, we used leafcutter version 0.2.9 (Li et al, 2018) rMATS version 4.1.2 (Shen et al, 2014), and Whippet version 0.11.1 (with Julia 1.6.6) (Sterne-Weiler et al, 2018) with the default parameters. We defined significantly differentially used events as events with a *P* value < 0.05 (for leafcutter and rMATS) or a confidence >0.95 (for Whippet).

### Immunofluorescence staining

Human standard kidney sections were deparaffinized in xylene and rehydration in a descending ethanol series followed by an unmasking step in Tris EDTA (pH 9.0) or in citrate buffer (0.1 M, pH 6.0) by heating for 5 min in a pressure cooker. We quenched autofluorescence by incubating the sections in 100 mmol/L glycine diluted in purified water and washed them three times in PBS. We then incubated the slides for 1 h in a blocking solution containing 1% fetal bovine serum, 1% normal goat serum, 1% cold fish gelatin, and 1% bovine serum albumin. The primary antibodies (rabbit anti-Epb41l5, (1:800), HPA037564, Sigma; rabbit anti- PLCE1, (1:100), 55463-1-AP, Proteintech; rabbit anti-SRSF3, (1:300) 10916-1-AP, Proteintech; guinea pig anti-nephrin, (1:300), GP-N2, Progen; anti-synaptopodin (1:50), 61094, Progen) were incubated overnight at 4 °C. Samples were washed with 1× PBS for 3 × 5 min and incubated with Cy2- and Cy3-conjugated secondary antibodies (1:800) (Jackson ImmunoResearch Laboratories) at room temperature for 1 h. After additional washing, the sections were stained with 1 µg/ml DAPI (Sigma-Aldrich) for 5 min. The samples were mounted in Mowiol (Carl Roth) for fluorescence microscopy.

### Histology on human kidney biopsies

Kidney biopsies were received from the Department of Nephropathology, Institute of Pathology, University Hospital Erlangen, Germany. The use of remnant kidney biopsy material was approved by the Ethics Committee of the Friedrich-Alexander-University of Erlangen-Nürnberg, waiving the need for retrospective consent for the use of archived rest material. Sample fixation and preparation of the control, FSGS and MGN group were identical. Diagnoses were established by routine nephropathological assessment. For selected stainings (Figs. EV1B and EV3D), anonymized excess normal kidney tissue obtained from partial nephrectomies at the Department of Urology, University Medicine Greifswald, was used as non-diseased reference tissue. These samples were not included in quantitative comparisons between control and FSGS groups. The use of excess kidney tissue from tumor nephrectomies has been approved by the Ethics Committee of the University Medicine Greifswald (Ref. No. BB 075/14). All patients stated written informed consent. All experiments were performed in accordance with local guidelines overseen by the University Medicine Greifswald and University Greifswald, Greifswald, Mecklenburg-Western Pomerania. All procedures involving human material were conducted in accordance with the ethical standards of the responsible institutional committees, the WMA Declaration of Helsinki, and the Department of Health and Human Services Belmont Report.

### Laser scanning microscopy

Images were captured with an Olympus FV3000 confocal microscope (Olympus, Tokyo, Japan) with 20×/40×/60× oil immersion objectives and Olympus FV3000 CellSense software.

### Image analysis

Fluorescence intensity for EPB41L5 of individual glomeruli was quantified using Fiji (Schindelin et al, 2012). For each image, glomeruli were manually outlined as regions of interest (ROIs) and the mean value was measured. For SRSF3, fluorescence intensity was quantified only in DAPI-positive nuclei using the StarDist plugin within Fiji (ImageJ). All images were acquired with identical microscope settings and were analyzed using the same intensity and threshold parameters.

### Single-cell RNA-sequencing data processing and analysis

Gene expression matrices derived from glomerular single-cell RNA-sequencing experiments (control, nephrotoxic serum nephritis (NTS) day 1 and day 5, and doxorubicin nephropathy) were downloaded from the BroadSingleCell Portal (Chung et al, 2020) and imported into R (v4.2.0) and processed using the Seurat package (v5.0.1). Each sample was converted into a Seurat object using the CreateSeuratObject function with a minimum of 200 genes and 3 cells per feature. Cells were annotated by sample origin and merged to generate combined datasets for NTS (control, NTS day 1, and NTS day 5) and doxorubicin (control and doxorubicin) analyses. Quality control included filtering cells with <200 or >5000 detected genes, <1000 or >10,000 UMIs, or >5% mitochondrial content. Following normalization (NormalizeData), variable features were identified, and datasets were scaled with the ScaleData function. Principal component analysis (PCA) was conducted on variable genes, followed by dimensionality reduction via UMAP (RunUMAP) and clustering (FindNeighbors, FindClusters). Clusters were manually annotated using canonical glomerular cell markers (e.g., *Nphs1* (podocytes), *Pecam1* (endothelial cells), *Pdgfrb* (mesangial cells), *Adgre1* (leukocytes), *Cldn1* (parietal epithelial cells)). Subclusters of podocytes (POD), mesangial cells (MC), and endothelial cells (EC) were extracted and reanalyzed using the same clustering approach. Differential gene expression between clusters was determined using the FindAllMarkers function with thresholds of log₂ fold change >0.25 and minimum expression in 25% of cells. Data visualization included UMAPs, violin plots, feature plots, and heatmaps which were all generated in Seurat. Marker genes and expression profiles were exported as CSV files for further downstream analysis described under “statistical analysis”.

### Statistical analysis

The GraphPad Prism 10 software was used for statistical analysis of experimental data and preparation of graphs. Scatter plots indicate individual units used for statistical testing (samples or replicates), as specified in respective figure legends. Data are presented as mean ± SD (or SEM, as indicated). Statistical analysis was performed using an unpaired two-tailed *t* test (*n* =  number of independent experiments). For multiple groups, statistical analyses were done by ANOVA with a Benjamini–Hochberg post-hoc test. Statistical significance was defined as *P* < 0.05 and significance levels are indicated as *P < 0.05, **P < 0.01, ***P < 0.001, ****P < 0.0001 and non-significant (ns) in respective figure panels. The number of independent experiments and analyzed units are stated in the figure legends.

### Graphics

Schematic illustrations in Figs. 1A, 3L and 5H, as well as the graphical abstract (synopsis image), were created using BioRender.com.

## Supplementary information

Appendix

Peer Review File

Dataset EV1

Dataset EV2

Source data Fig. 1

Source data Fig. 2

Source data Fig. 3

Source data Fig. 4

Source data Fig. 5

Source data Fig. 6

Source data Fig. 7

Expanded View Figures

## Data Availability

RNA-seq analysis of (differentially) expressed genes in whole zebrafish larvae and zebrafish glomeruli after NFP-treatment (FSGS induction) using DESeq2 are available in Dataset EV1. The comprehensive list of all identified AS (alternative spliced) genes is provided in Dataset EV2. The RNA-Seq data (the raw data and the preprocessed data) has been deposited at the GEO database (GSE301334). The source data of this paper are collected in the following database record: biostudies:S-SCDT-10_1038-S44321-026-00495-5.
