# Supplementary material for: Disease-driven post-transcriptional alterations and alternative splicing in podocytes in focal segmental glomerulosclerosis
Source: EMBO Mol Med. 2026 Aug 13;18(9):3824–49. doi: 10.1038/s44321-026-00495-5 (PMC13562739; doi:10.1038/s44321-026-00495-5)
Supplement: Supplementary file 1 [file 44321_2026_495_MOESM12_ESM.pdf]

Expanded View Figures

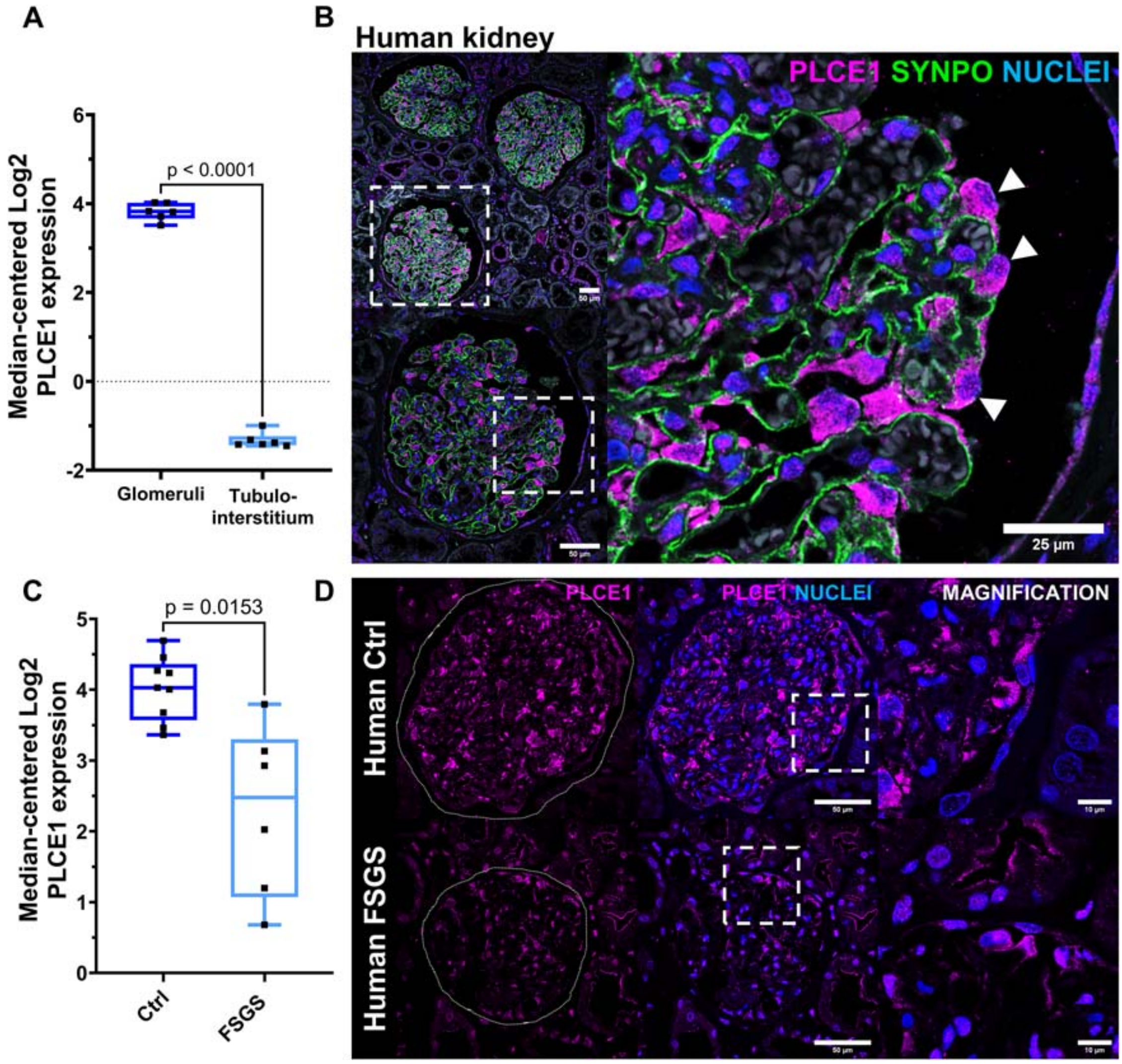

Figure EV1. ***PLCE1*** **expression in healthy human kidney and FSGS patients.**

This figure validates *PLCE1* as a podocyte-enriched gene that is downregulated in human FSGS, supporting the cross-species concordance shown in Fig. 3I of the main text. Human data were retrieved from the Nephroseq platform (Lindenmeyer Normal Tissue Panel for (**A**) (Data ref: Lindenmeyer et al, 2010) and Hodgin FSGS Glom Dataset for panel C (Data ref: Hodgin et al, 2010)). (**A**) Microarray data showing significantly higher *PLCE1* expression in glomeruli ($n = 7$) vs. tubulointerstitium ($n = 7$) in healthy human kidney tissue. (**C**) Glomerular *PLCE1* mRNA expression in healthy control (Ctrl) ($n = 9$) vs. FSGS ($n = 6$) patients. (**B**, **D**) Immunofluorescence for PLCE1 (magenta), synaptopodin (green), and DAPI (blue) in healthy human kidney (**B**) and FSGS biopsy (**D**). Podocytes are indicated by arrowheads. Scale bars: 50 µm, 25 µm, 10 µm. Statistical significance was assessed using Welch's *t* test (**A**, **C**); *P* values is indicated. Box plots show the median (center line), 25th and 75th percentiles (box boundaries), and minimum and maximum values (whiskers) (**A**, **C**).

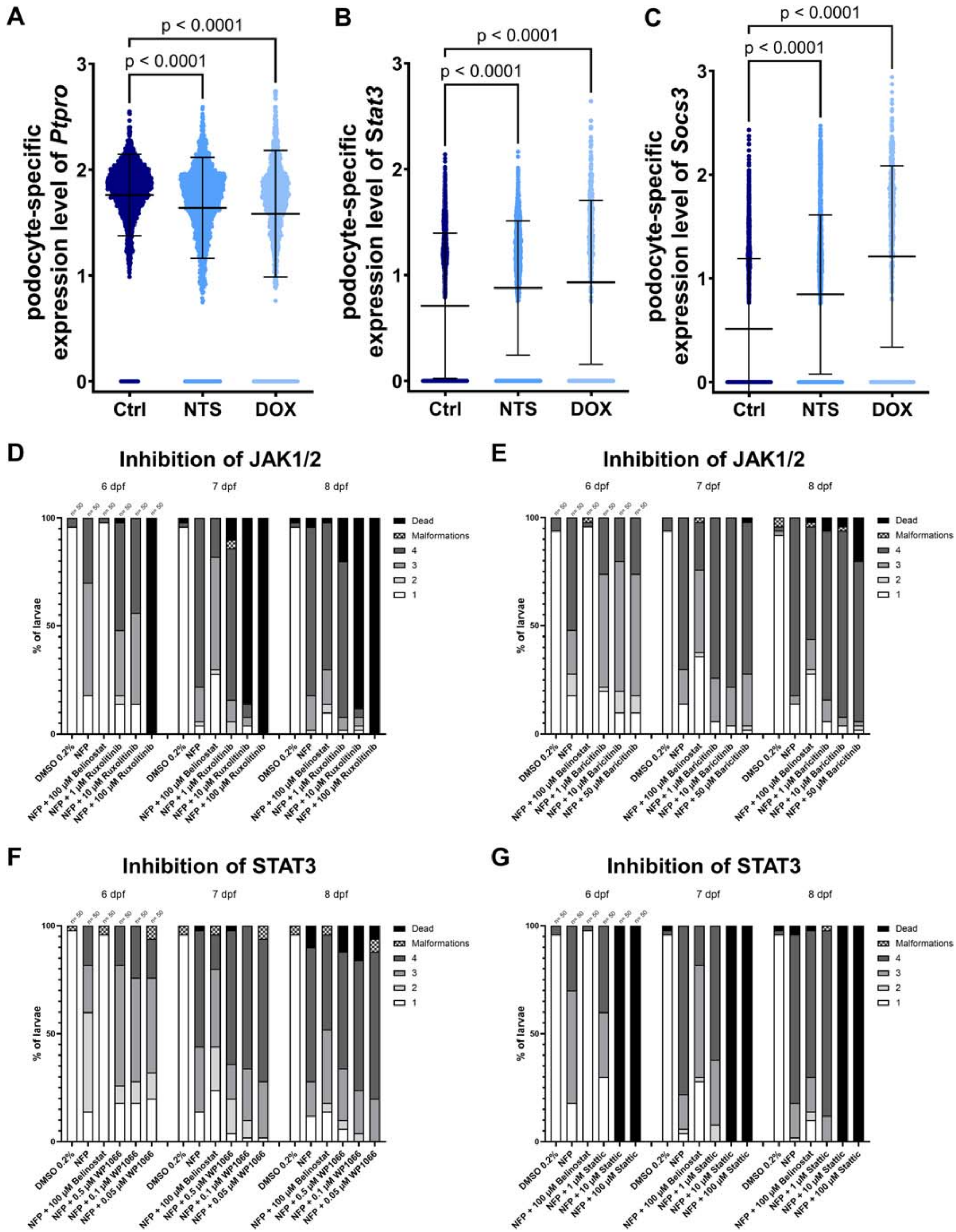
A
p < 0.0001
p < 0.0001
podocyte-specific expression level of Ptpro
Ctrl
NTS
DOX
B
podocyte-specific expression level of Stat3
C
podocyte-specific expression level of Socs3
D
Inhibition of JAK1/2
6 dpf
7 dpf
8 dpf
% of larvae
Dead
Malformations
DMSO 0.2%
NFP
NFP + 100 µM Belinostat
NFP + 1 µM Ruxolitinib
NFP + 10 µM Ruxolitinib
NFP + 100 µM Ruxolitinib
E
Inhibition of JAK1/2
NFP + 1 µM Baricitinib
NFP + 10 µM Baricitinib
NFP + 50 µM Baricitinib
F
Inhibition of STAT3
NFP + 0.5 µM WP1066
NFP + 0.1 µM WP1066
NFP + 0.05 µM WP1066
G
Inhibition of STAT3
NFP + 1 µM Stattic
NFP + 10 µM Stattic
NFP + 100 µM Stattic

 © The Author(s)

◀ **Figure EV2. Stat3 expression increases in different kidney injury models, but inhibition of the JAK1/2-STAT3 pathway does not confer protective effects after FSGS induction in zebrafish larvae.**

This figure provides cross-species validation of STAT3 pathway activation (**A**–**C**) and documents the results of pharmacological inhibition experiments (**D**–**G**) referenced in the main text. (**A**–**C**) Podocyte-specific expression of *Ptpro* (**A**), *Stat3* (**B**), and *Socs3* (**C**) from published mouse glomerular scRNA-seq data (Chung et al, 2020) in nephrotoxic serum nephritis (NTS) and doxorubicin (DOX) models vs. control (*Ptpro*: Ctrl: $n = 2018$; NTS: $n = 1639$; DOX: $n = 1434$; *Stat3*: Ctrl: $n = 2018$; NTS: $n = 1434$; DOX: $n = 504$; *Socs3*: Ctrl: $n = 2066$; NTS: $n = 1434$; DOX: $n = 504$). (**D**–**G**) Bar graphs showing edema severity scores in zebrafish larvae ($n = 50$ per group) at 6, 7, and 8 dpf after NFP treatment (50 nM) co-administered with JAK1/2 inhibitors (Ruxolitinib, **D**; Baricitinib, **E**) or STAT3 inhibitors (WP1066, **F**; Stattic, **G**). Edema scores: 1 = no edema, 2 = mild edema, 3 = medium edema, 4 = severe edema with bent body axis and malformations or dead. Belinostat (100 µM) served as positive control. All inhibitors were administered concomitantly with NFP from 4 dpf. None of the inhibitors showed protective effect. Instead, treatment accelerated disease progression in the FSGS injury model, as evidenced by enhanced edema formation and increased mortality rates. Statistical significance was assessed using one-way ANOVA followed by Benjamini–Hochberg correction (**A**– **C**); *P* values are indicated; mean ± SD.

© The Author(s)

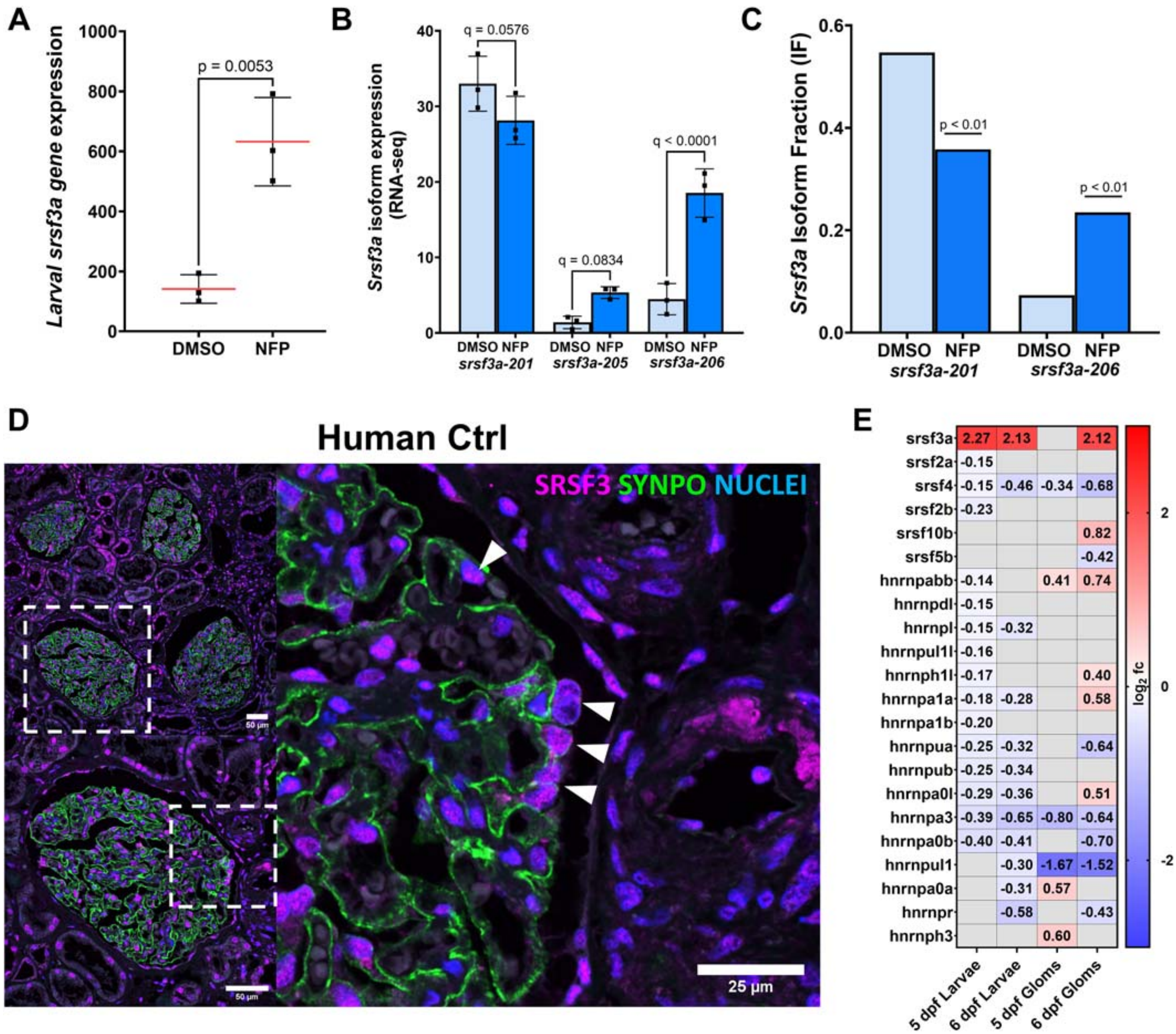

Figure EV3. SRSF3 isoform regulation in zebrafish larvae and nuclear localization in human kidney.

This figure supplements Fig. 6 of the main text by providing srsf3a isoform data from whole larvae (**A–C**) alongside the glomerular data shown in Fig. 6A–C, and additional human validation data. (**A–C**) Gene expression (**A**), isoform expression (**B**; DESeq2-normalized counts), and isoform fraction (**C**; proportion of each isoform relative to total *srsf3a* expression) of *srsf3a* in zebrafish whole larvae at 6 dpf (NFP vs. DMSO; $n = 3$ biological replicates per condition; mean ± SD; unpaired *t* test). All gene annotations: Ensembl release 113. (**D**) Confocal images of human control kidney sections showing nuclear SRSF3 immunoreactivity (magenta) within glomerular cells; synaptopodin (green) labels podocytes; nuclei stained with DAPI (blue). Arrowheads indicate podocytes. Scale bars: 50 µm and 25 µm. SRSF3 signal is detected in multiple glomerular cell types; podocyte co-localization is confirmed by synaptopodin overlap (arrowheads in magnification inset). (**E**) Heatmaps of gene expression levels of all SRSF-family and hnRNP-family members in zebrafish larvae and glomeruli (log2 fold change, NFP vs. DMSO; $P < 0.05$, DESeq2). Red: upregulated; blue: downregulated. Statistical significance was assessed using unpaired *t* test (**A**), and one-way ANOVA followed by Benjamini–Hochberg correction (**B**); *q* values and *P* values are indicated; mean ± SD.

 © The Author(s)

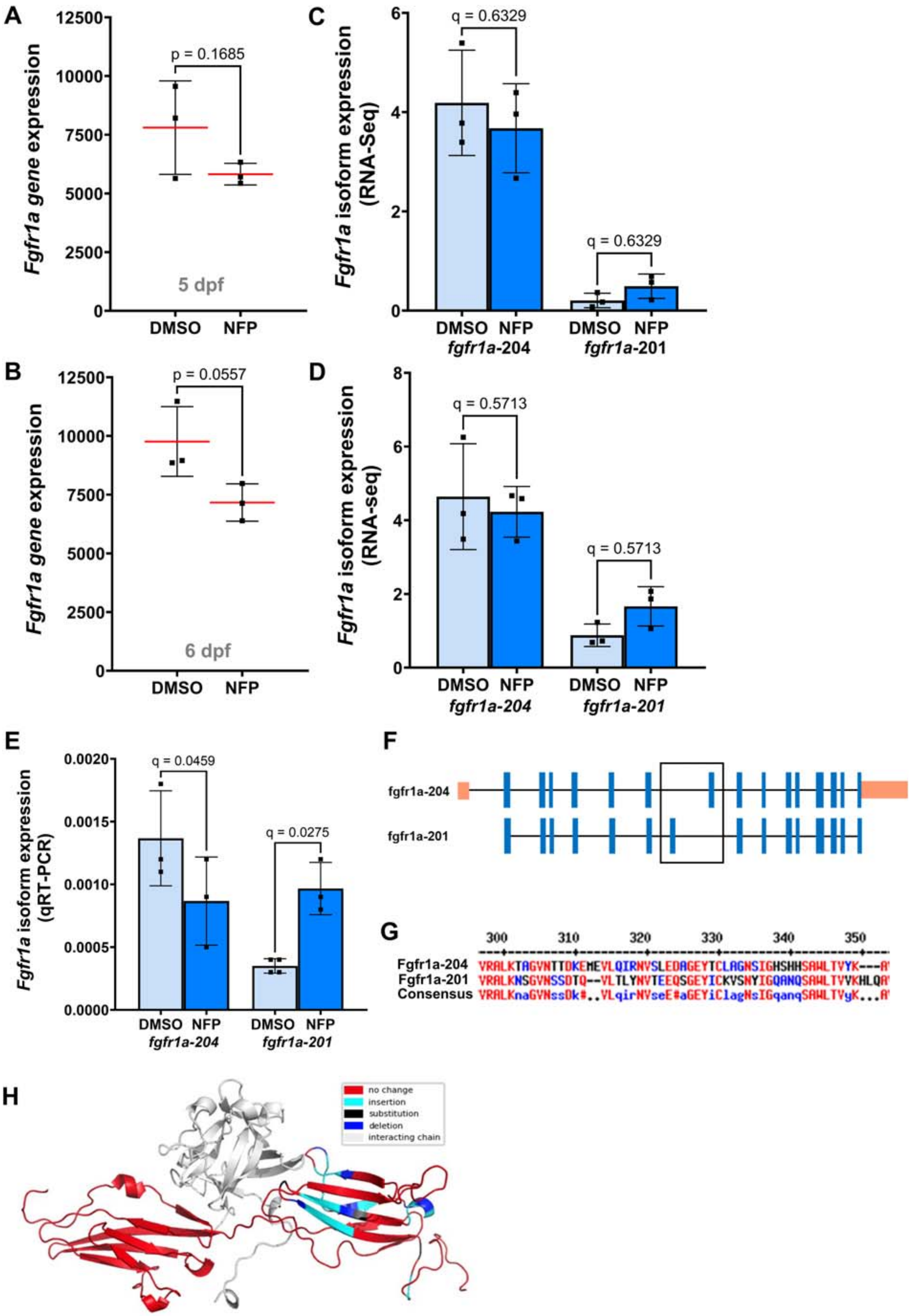
A
Fgfr1a gene expression
p = 0.1685
5 dpf
DMSO NFP
B
Fgfr1a gene expression
p = 0.0557
6 dpf
DMSO NFP
C
Fgfr1a isoform expression (RNA-Seq)
q = 0.6329
q = 0.6329
DMSO NFP fgfr1a-204
DMSO NFP fgfr1a-201
D
Fgfr1a isoform expression (RNA-seq)
q = 0.5713
q = 0.5713
DMSO NFP fgfr1a-204
DMSO NFP fgfr1a-201
E
Fgfr1a isoform expression (qRT-PCR)
q = 0.0459
q = 0.0275
DMSO NFP fgfr1a-204
DMSO NFP fgfr1a-201
F
fgfr1a-204
fgfr1a-201
G
Fgfr1a-204
Fgfr1a-201
Consensus
H
no change
insertion
substitution
deletion
interacting chain

Figure EV4. Regulation of *fgfr1a* isoforms in FSGS-injured zebrafish glomeruli.

This figure documents the isoform switch in *fgfr1a*, a receptor tyrosine kinase, in FSGS-injured glomeruli, demonstrating that alternative splicing can alter signaling capacity independently of overall gene expression changes. Dataset: isolated zebrafish glomeruli, NFP vs. DMSO. (**A**, **B**) *Fgfr1a* gene expression at 5 dpf (**A**) and 6 dpf (**B**) ($n = 3$ biological replicates). (**C**, **D**) Glomerular *fgfr1a* isoform expression at 5 dpf (**C**) and 6 dpf (**D**) ($n = 3$ biological replicates) (DESeq2-normalized counts). (**E**) Validation of *fgfr1a* isoform expression by qRT-PCR (normalized to DMSO glomeruli and Gapdh; $n = 3$ or 4 biological replicates). (**F**) Schematic of *fgfr1a* isoforms (blue: coding exons; orange: UTRs). (**G**) Protein sequence alignment of the two Fgfr1a isoforms highlighting isoform-specific amino acid differences. (**H**) Structural analysis comparing Fgfr1a isoforms 204 (ENSDART00000147742) and 201 (ENSDART00000024882): red = no change; cyan = insertion; black = substitution; blue = deletion; gray = FGF2 interaction chain. The isoform switch is predicted to reduce FGF2 binding affinity. All gene annotations: Ensembl release 113. Statistical significance was assessed using unpaired *t* test (**A**, **B**), one-way ANOVA followed by Benjamini–Hochberg correction (**C–E**); *q* values and *P* values are indicated; mean ± SD.

 © The Author(s)
