# Supplementary material for: Disease-driven post-transcriptional alterations and alternative splicing in podocytes in focal segmental glomerulosclerosis
Source: EMBO Mol Med. 2026 Aug 13;18(9):3824–49. doi: 10.1038/s44321-026-00495-5 (PMC13562739; doi:10.1038/s44321-026-00495-5)
Supplement: Supplementary file 2 [file 44321_2026_495_MOESM1_ESM.pdf]

Appendix

Disease-driven post-transcriptional alterations and alternative splicing of podocytes in focal segmental glomerulosclerosis

Francescapaola Mattias[1], Olga Tsoy[2], Anna Iervolino[3], Stefan Simm[4], Elke Hammer[5], Alexander Gress[6], Florian Siegerist[1,7,8], Maximillian Schindler[1], Tim Lange[1], Sabine Ameling[5], Tim Kacprowski[9,10], Markus List[11,12], Olga Kalinina[6], Sören Franzenburg[13], Giovambattista Capasso[3,14], Karlhans Endlich[1], Jan Baumbach[2], Uwe Völker[5], Nicole Endlich[1,15#], Felix Kliewe[1*#]

[1] Department of Anatomy and Cell Biology, University Medicine Greifswald, Greifswald, Germany; [2] Institute for Computational Systems Biology, University of Hamburg, Hamburg, Germany; [3] Institute of Molecular Biology and Genetics (Biogem) Ariano Irpino (Italy); [4] Coburg University of Applied Sciences, Institute of Bioanalysis, Coburg, Germany; [5] Interfaculty Institute for Genetics and Functional Genomics, University Medicine Greifswald, Greifswald, Germany; [6] Department of Drug Bioinformatics, Helmholtz Institute for Pharmaceutical Research Saarland (HIPS), Helmholtz Centre for Infection Research (HZI), Germany; [7] Neonatology and Pediatric Intensive Care Medicine, Department of Pediatrics, University Medicine Greifswald, Greifswald, Germany; [8] German Center for Child and Adolescent Health (DZKJ), Partner Site Greifswald, Rostock, Germany; [9] Institute of Data Science in Biomedicine, Technische Universität Braunschweig, Braunschweig, Germany; [10] Braunschweig Integrated Centre of Systems Biology (BRICS), TU Braunschweig, Braunschweig, Germany; [11] Data Science in Systems Biology, TUM School of Life Sciences, Technical University of Munich, Freising, Germany; [12] Munich Data Science Institute, Technical University of Munich, Garching, Germany; [13] Institute of Clinical Molecular Biology (IKMB), Kiel University, Kiel, Germany; [14] Department of Translational Medical Science, University of Campania Luigi Vanvitelli, Naples (Italy); [15] NIPOKA GmbH, Center of High-End Imaging, Greifswald, Germany

# These authors contributed equally
* Corresponding authors:

Dr. Felix Kliewe
Department of Anatomy and Cell Biology, University Medicine Greifswald
Friedrich-Loeffler-Str. 23c, 17489 Greifswald, Germany
Email: felix.kliewe@uni-greifswald.de
https://orcid.org/0000-0001-8717-5927

Prof. Dr. Nicole Endlich
Department of Anatomy and Cell Biology, University Medicine Greifswald
Friedrich-Loeffler-Str. 23c, 17489 Greifswald, Germany
Email: nicole.endlich@uni-greifswald.de

Table of Contents

Appendix Figure S1	GSEA (gene set enrichment analysis) of significantly regulated genes in FSGS zebrafish larvae - Molecular Function and Cellular Component ontologies.	Page 2
Appendix Figure S2	Detailed gene set enrichment analysis (GSEA) of significantly regulated genes in FSGS zebrafish glomeruli, including developmental gene clusters.	Page 3
Appendix Figure S3	Detailed gene set enrichment analysis (GSEA) of significantly regulated genes in FSGS zebrafish glomeruli - Molecular Function (MF) and Cellular Component (CC) ontologies.	Page 4
Appendix Figure S4	Detection of alternative splicing events across multiple analysis tools.	Page 5
Appendix Figure S5	Kidney cell type expression patterns of alternatively spliced candidate genes.	Page 6
Appendix Figure S6	Kidney cell type expression of alternatively spliced candidates at 6 dpf.	Page 7
Appendix Figure S7	Glomerular *epb41l5* isoform expression 24h after FSGS induction.	Page 8
Appendix Table S1	Used qRT-PCR Primer.	Page 9

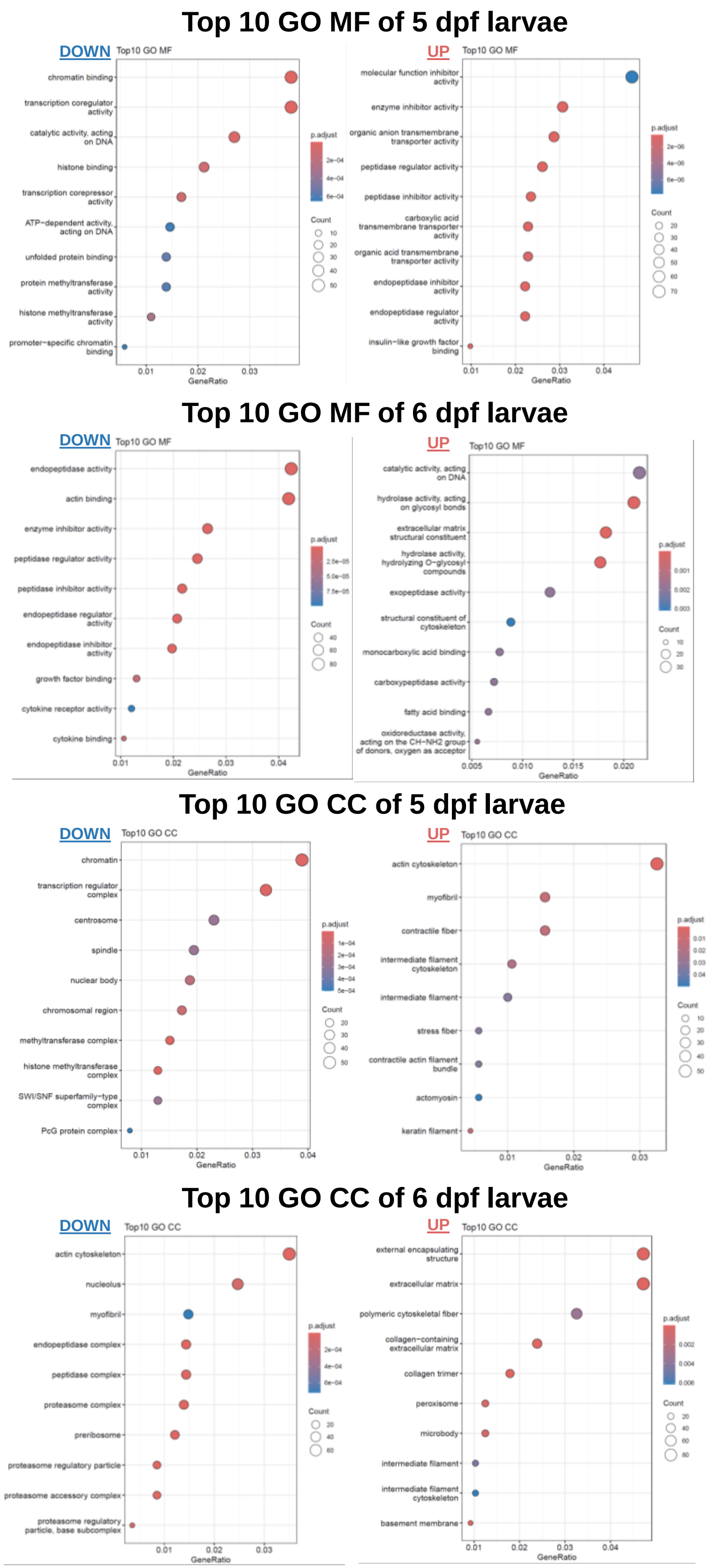

Appendix Fig. S1: GSEA (gene set enrichment analysis) of significantly regulated genes in FSGS zebrafish larvae - Molecular Function and Cellular Component ontologies.
This figure provides an extended Gene Ontology (GO) analysis of the RNA-seq data from FSGS-induced zebrafish larvae (NFP vs. DMSO control; n = 3 biological replicates *per* condition; 5 and 6 dpf). RNA-seq-based enriched Top 10 GO clusters of significantly regulated genes in FSGS zebrafish larvae are shown, categorized by molecular function (MF) and cellular component (CC) ontologies at 5 dpf (upper panels) and 6 dpf (lower panels), complementing the biological process (BP) data shown in Figure 2G–H of the main text. DOWN: down-regulated DEGs; UP: up-regulated DEGs. Dot size represents gene count; color indicates adjusted p-value.

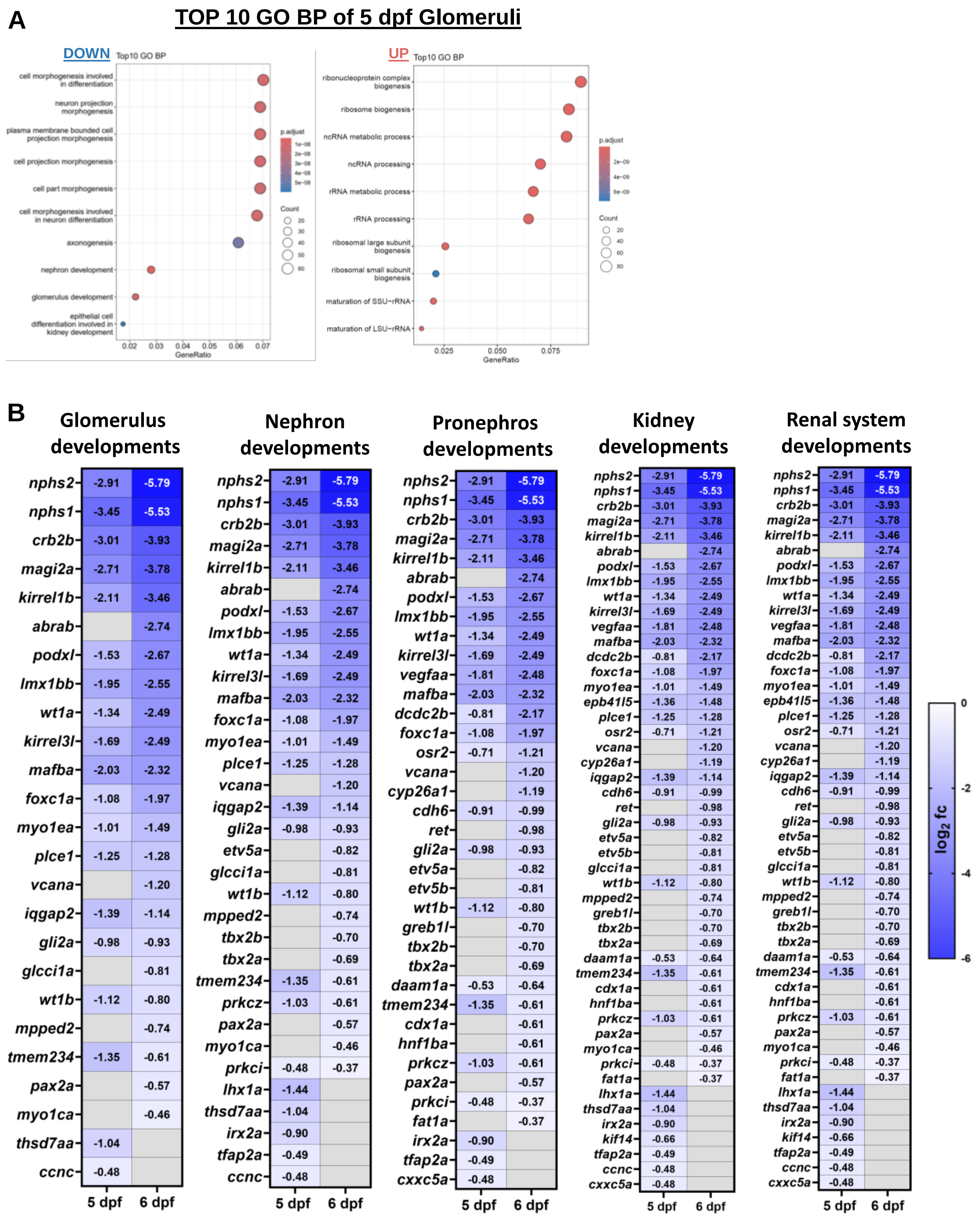

Appendix Fig. S2: Detailed gene set enrichment analysis (GSEA) of significantly regulated genes in FSGS zebrafish glomeruli, including developmental gene clusters.

This figure extends the GO analysis of Figure 3G to include additional ontology categories and a comprehensive developmental gene cluster heatmap. Dataset: isolated zebrafish glomeruli, NFP vs. DMSO, *n* = 3 biological replicates *per* condition, 5 and 6 dpf. (A) Top 10 enriched GO biological process (BP) clusters from 5 dpf zebrafish FSGS glomeruli. (B) Heatmap of DEGs in isolated zebrafish glomeruli at 5 and 6 dpf related to glomerulus, nephron, pronephros, kidney, and renal system development gene clusters (DESeq2; adj. p-value ≤ 0.05). Log2 fold change; blue: down-regulated vs. DMSO controls.

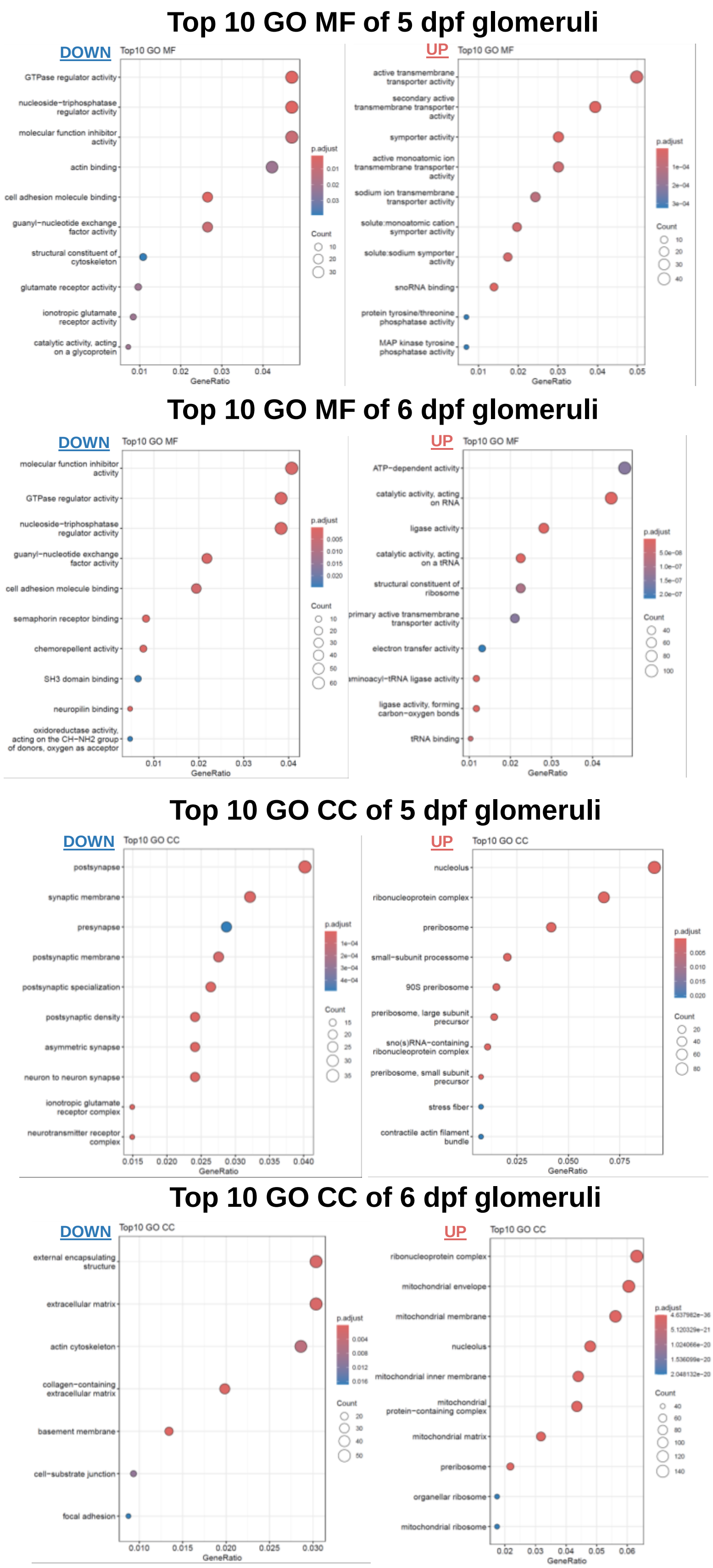

Appendix Fig. S3: Detailed gene set enrichment analysis (GSEA) of significantly regulated genes in FSGS zebrafish glomeruli - Molecular Function (MF) and Cellular Component (CC) ontologies.
This figure provides MF and CC GO analyses for isolated glomeruli, complementing the BP data in Figure 3G and the larval data in Figure S1. Dataset: isolated zebrafish glomeruli, NFP vs. DMSO, n = 3 biological replicates *per* condition. Top 10 GO MF and CC clusters from 5 dpf and 6 dpf zebrafish FSGS glomeruli are shown. DOWN: down-regulated DEGs; UP: up-regulated DEGs. Dot size represents gene count; color indicates adjusted p-value.

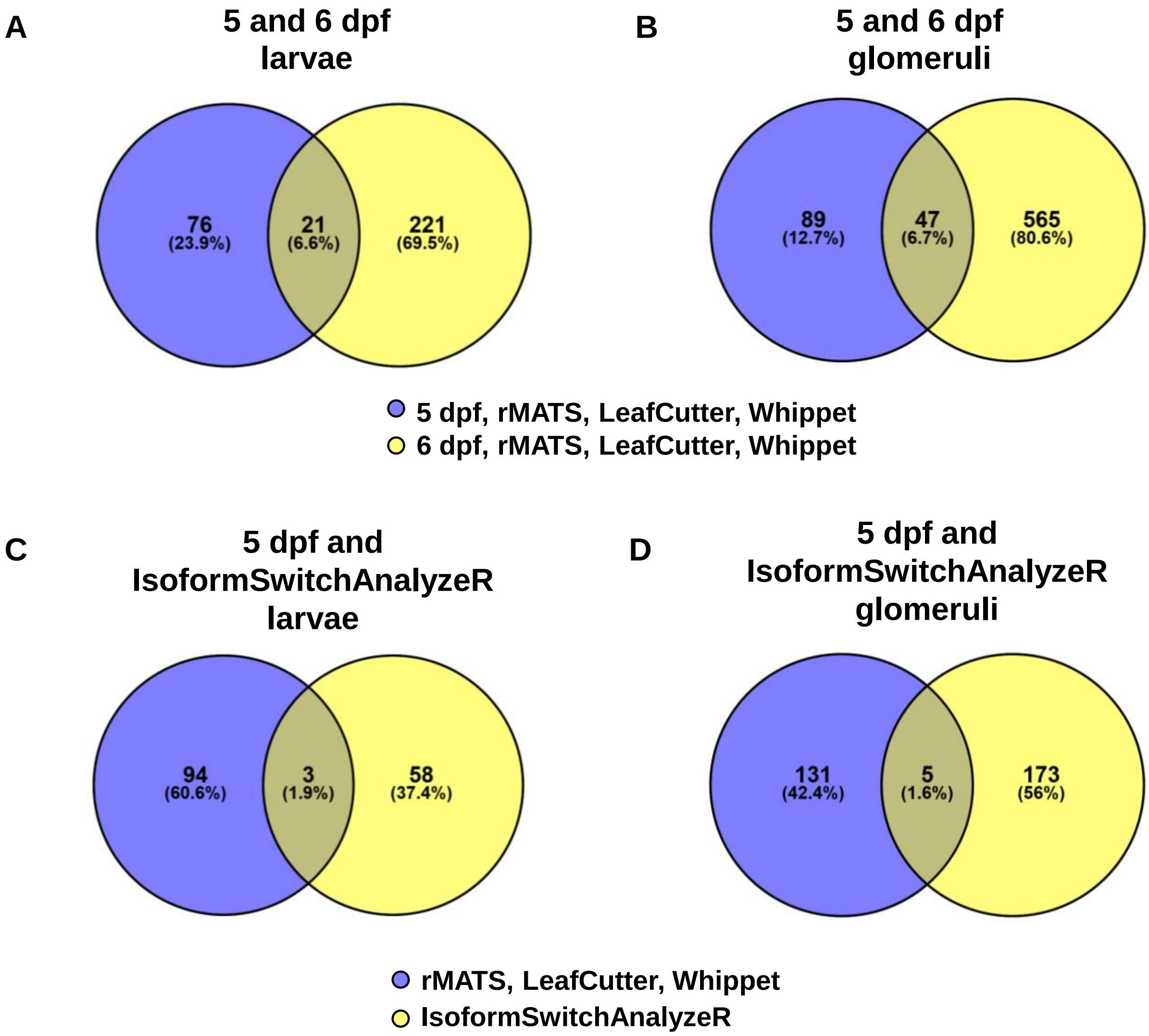

Appendix Fig. S4: Detection of alternative splicing events across multiple analysis tools.
This figure provides a comprehensive overview of the overlap between alternative splicing (AS) detection tools, complementing Figure 5D-G of the main text. (A, B) Venn diagrams showing overlap of alternatively spliced genes detected by rMATS, LeafCutter, and Whippet in whole larvae (A) and isolated glomeruli (B) at 5 and 6 dpf (NFP vs. DMSO; n = 3 biological replicates *per* condition; significance thresholds: $p < 0.05$ for rMATS and LeafCutter; confidence > 0.95 for Whippet). (C, D) Venn diagrams showing the overlap between AS events detected by the three tools combined and isoform switches identified by IsoformSwitchAnalyzeR in whole larvae (C) and isolated glomeruli (D) at 5 dpf. A comprehensive list of all alternatively spliced genes is provided in Dataset EV3.

Alternative spliced genes found at 5 and 6 dpf in zebrafish glomeruli by rMATS, LeafCutter, Whippet

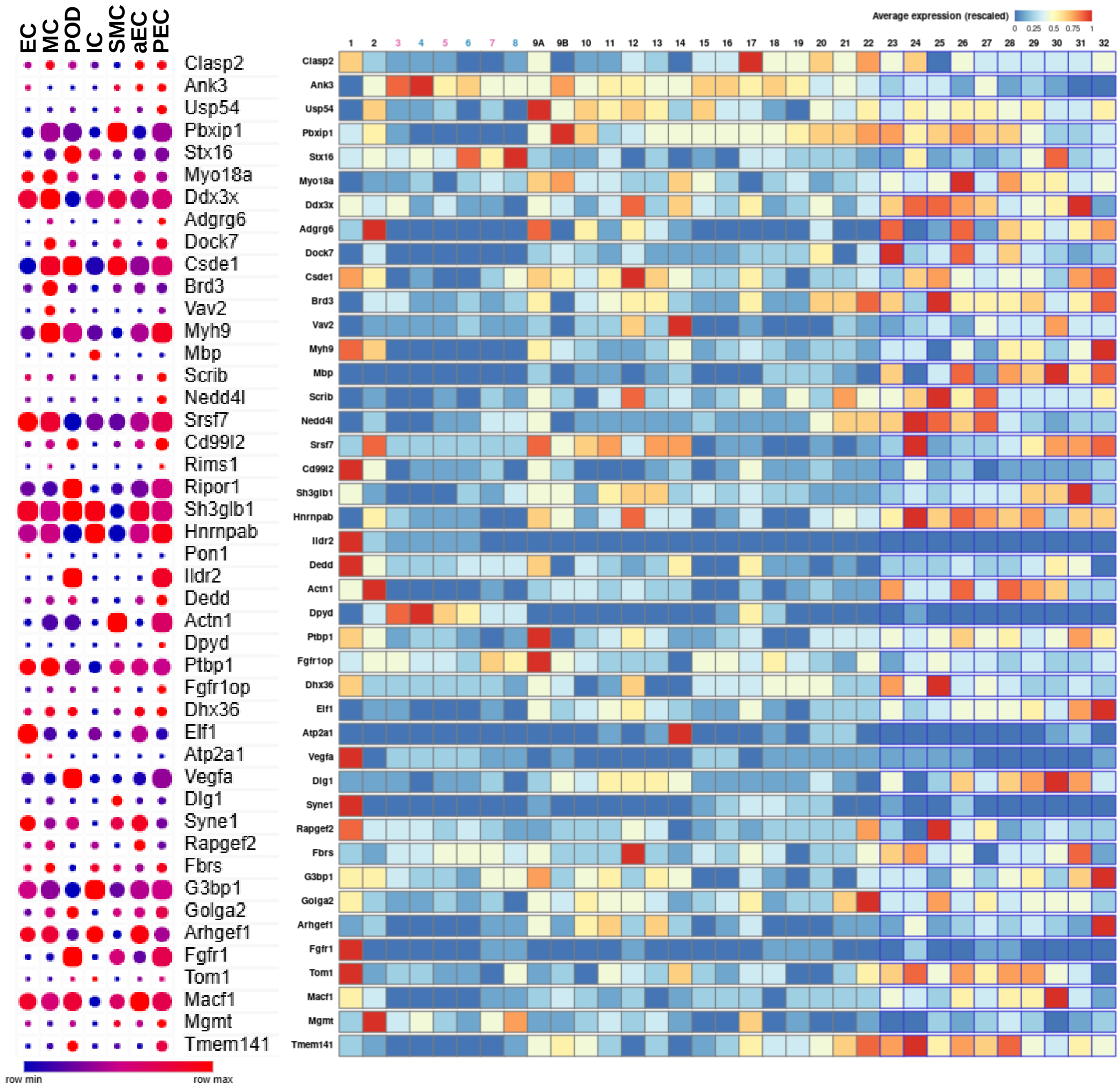

Alternative spliced genes found at 5 dpf glomeruli by rMATS, LeafCutter, Whippet and IsoformsSwitchAnalyzeR

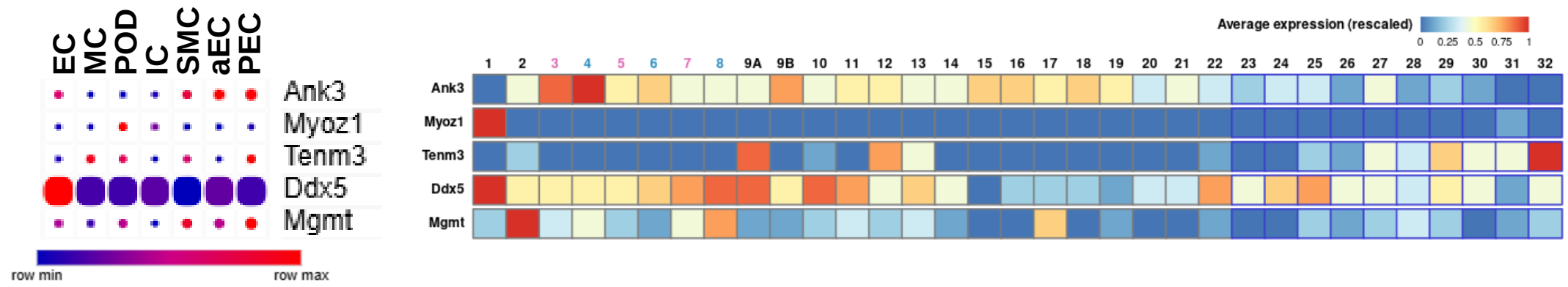

Appendix Fig. S5: Kidney cell type expression patterns of alternatively spliced candidate genes. This figure characterizes the nephron cell type expression specificity of alternatively spliced genes identified in Figure 5 and Figure S4, using two independent publicly available mouse single-cell RNA-seq datasets. Upper panel: Expression heatmap of alternatively spliced genes identified at both 5 and 6 dpf in zebrafish FSGS glomeruli by rMATS, LeafCutter, and Whippet (union set; NFP vs. DMSO; *n* = 3 biological replicates *per* condition). Dataset: mouse whole-kidney scRNA-seq (Ransick et al, 2019; PMID: 31689386) and mouse glomerular scRNA-seq (Chung et al, 2020; PMID: 32651223). Lower panel: Expression heatmap of alternatively spliced genes identified at 5 dpf zebrafish glomeruli by rMATS, LeafCutter, Whippet, and IsoformSwitchAnalyzeR combined (NFP vs. DMSO). Same datasets as upper panel. Color code: red = high average expression (row max); blue = low expression (row min); all values are row-normalized. Columns represent kidney cell types 1–32 as defined in Ransick et al. (2019): (1) podocytes, (2) parietal epithelium, (3–8) proximal tubule segments, (9A–14) loop of Henle segments, (15–17) thick ascending limb/macula densa, (18–20) distal tubule/connecting tubule, (21–32) collecting duct subtypes. Left-hand column annotations indicate expression across glomerular cell clusters from Chung et al. (2020): EC = glomerular endothelial cells; MC = mesangial cells; POD = podocytes; IC = immune cells; SMC = smooth muscle cells; aEC = arteriolar endothelial cells; PEC = parietal epithelial cells.

Alternative spliced genes found at 6 dpf glomeruli by rMATS, LeafCutter, Whippet and IsoformsSwitchAnalyzeR

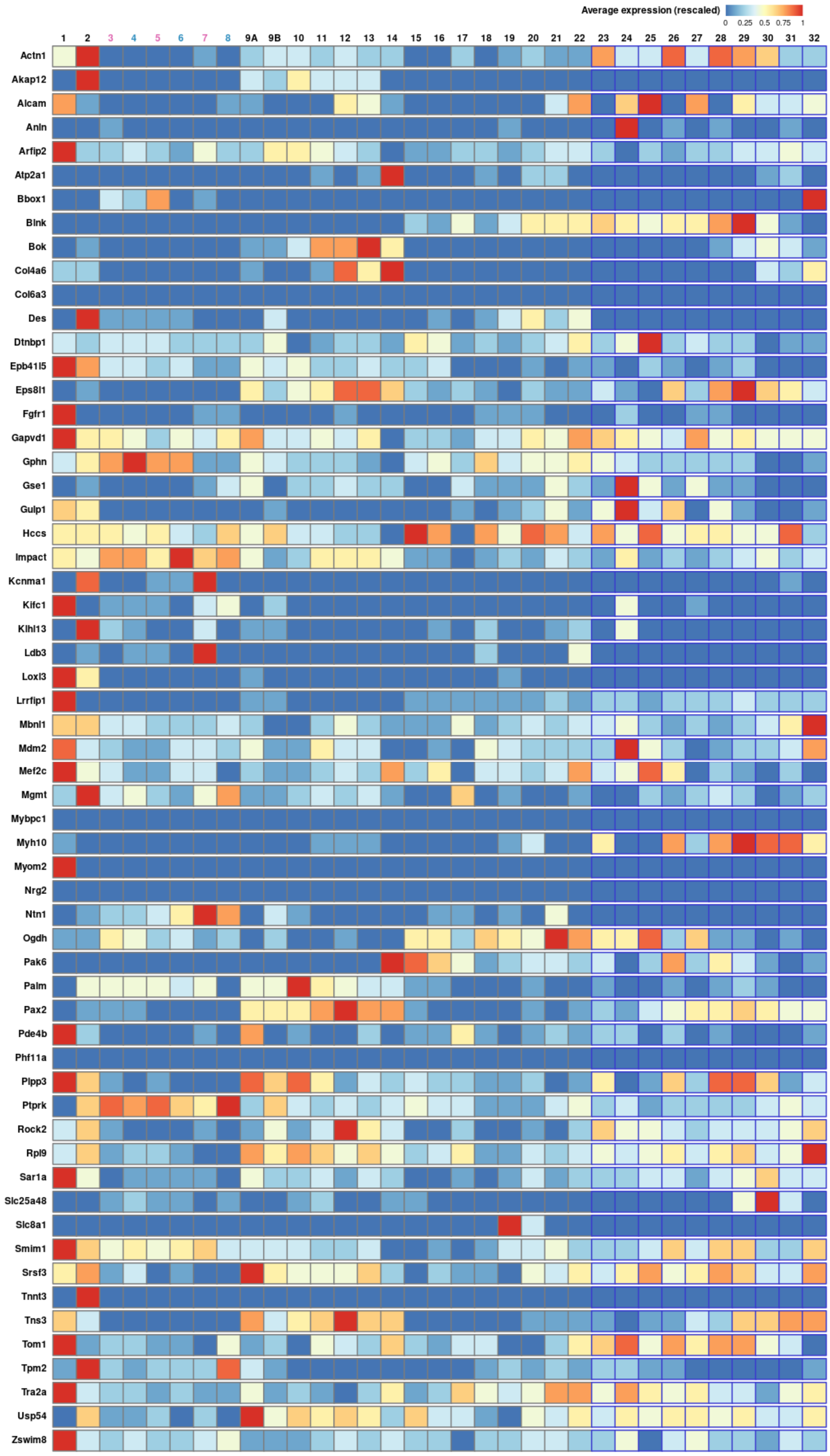

Appendix Fig. S6: Kidney cell type expression of alternatively spliced candidates at 6 dpf.
This figure extends Figure S5 to the 6 dpf time point and the combined tool/IsoformSwitchAnalyzeR gene set shown in Figure 5J of the main text. Dataset: mouse whole-kidney scRNA-seq (Ransick et al, 2019; PMID: 31689386; https://cello.shinyapps.io/kidneycellexplorer/). Condition: alternatively spliced genes identified at 6 dpf in zebrafish FSGS glomeruli by rMATS, LeafCutter, Whippet, and IsoformSwitchAnalyzeR (NFP vs. DMSO; *n* = 3 biological replicates *per* condition). Color code and column labels as in Appendix Figure S5.

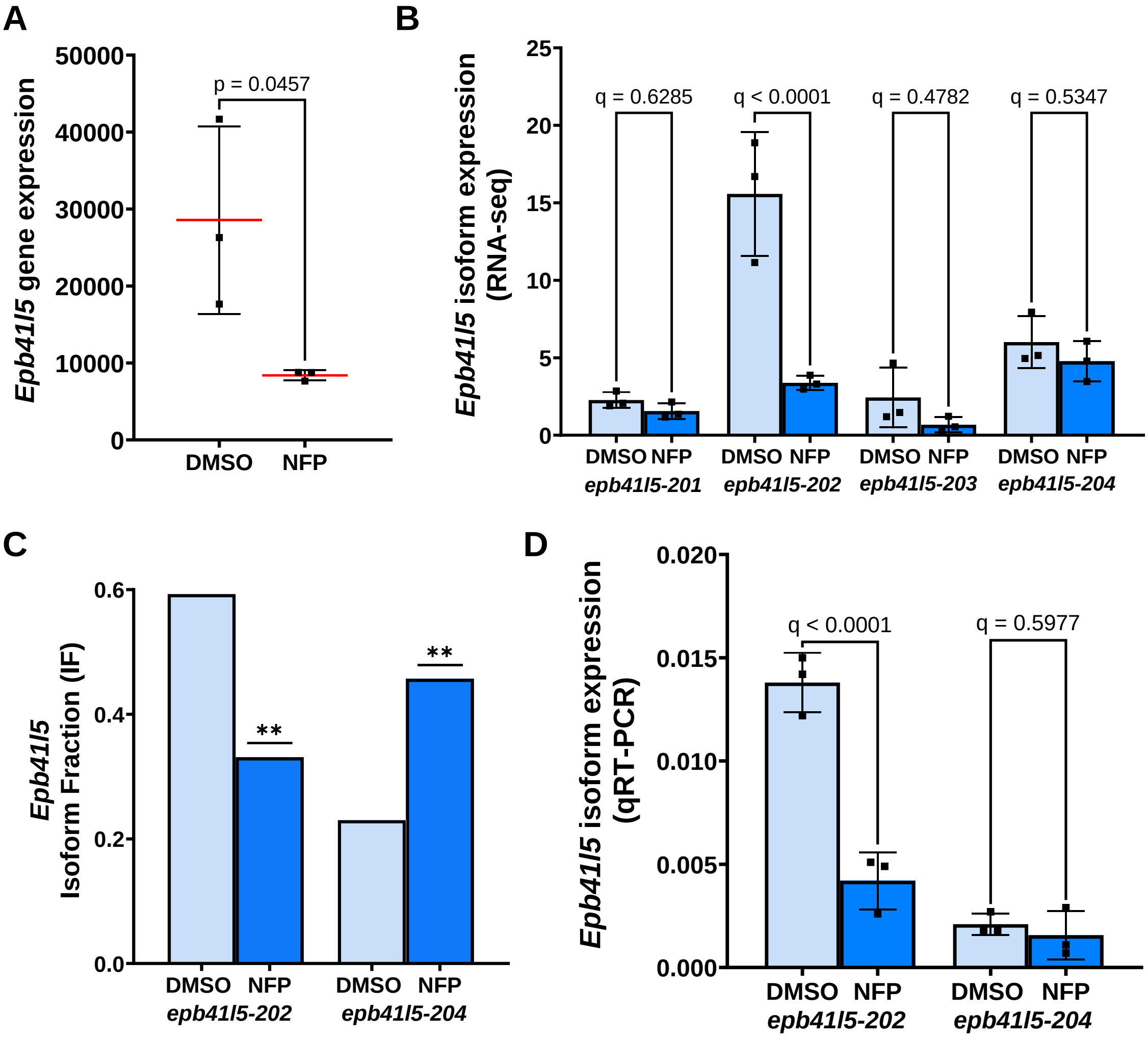

Appendix Fig. S7: Glomerular *epb41l5* isoform expression 24h after FSGS induction.
This figure provides the 5 dpf time point data for *epb41l5* isoform regulation, complementing the 6 dpf data shown in Figure 7 of the main text. Dataset: isolated zebrafish glomeruli, NFP vs. DMSO, 5 dpf (*n* = 3 biological replicates *per* condition). (A) Gene expression of *epb41l5* at 5 dpf. (B) Isoform expression of *epb41l5* variants (DESeq2-normalized counts). (C) Isoform fraction (IF) of *epb41l5*-202 and *epb41l5*-204 (IsoformSwitchAnalyzeR; ** p < 0.01; mean ± SD). (D) Validation of the *epb41l5*-204 isoform switch by qRT-PCR, normalized to DMSO-treated glomeruli and *Gapdh* (*n* = 3 biological replicates). Statistical significance was assessed using unpaired t-test (A), one-way ANOVA followed by Benjamini–Hochberg correction (B, D); q-values and p-values are indicated; mean ± SD.

Appendix Table S1:

Used qRT-PCR Primer

**Detected gene**	**Sequence / Identity**
ef1a1l1	F: AAGGAGGGTAATGCTAGCGG<br>R: GGGCGAAGGTCACAACCATA
epb41l5<br>epb41l5-202	F: TCGACTGTCCCACAAAGGTG<br>R: CTTGGTCGAGTGAGCCACAA
epb41l5<br>epb41l5-204	F: CAGGCCCCATGGAGATTGAA<br>R: GACGGTCACTCTGGTGTCAG
fgfr1a<br>*fgfr1a*-204	F: GCGATCCTCAGCCTCACATT<br>R: GTGATGGGAGTGGCCGATAG
fgfr1a<br>*fgfr1a*-201	F: TGACCGAAGAACAAAGCGGA<br>R: CACGCAGTAGATCAGCACCT
srsf3a<br>*srsf3a*-201	F: ACGAGGTGTTAGGTGCGTTT<br>R: GACCTTACAGTCTAGCGGGC
srsf3a<br>*srsf3a*-206	F: CGTCCGTGTGTTTATTCGCC<br>R: ACCGGAAGGTGTTCTGAGATG
