# Supplementary material for: Disease-driven post-transcriptional alterations and alternative splicing in podocytes in focal segmental glomerulosclerosis
Source: EMBO Mol Med. 2026 Aug 13;18(9):3824–49. doi: 10.1038/s44321-026-00495-5 (PMC13562739; doi:10.1038/s44321-026-00495-5)
Supplement: Supplementary file 3 [file 44321_2026_495_MOESM2_ESM.pdf]

EMBO Molecular Medicine

Disease-driven post-transcriptional alterations and alternative splicing of podocytes in FSGS

Francescapaola Mattias, Olga Tsoy, Anna Iervolino, Stefan Simm, Elke Hammer, Alexander Gress, Florian Siegerist, Maximilian Schindler, Tim Lange, Sabine Ameling, Tim Kacprowski, Markus List, Olga Kalinina, Soren Franzenburg, Giovambattista Capasso, Karlhans Endlich, Jan Baumbach, Uwe Völker, Nicole Endlich, and Felix Kliewe

Corresponding author(s): Felix Kliewe (felix.kliewe@uni-greifswald.de) , Nicole Endlich (nicole.endlich@uni-greifswald.de)

Review Timeline:

Submission Date:	19th Jan 26
Editorial Decision:	21st Jan 26
Appeal Correspondence with the Editor	22-28 Jan 26
Appeal Received:	31st Mar 26
Editorial Decision:	5th May 26
Revision Received:	5th Jun 26
Editorial Decision:	21st Jun 26
Revision Received:	15th Jul 26
Accepted:	15th Jul 26

Editor: Lise Roth

Transaction Report:

(Note: With the exception of the correction of typographical or spelling errors that could be a source of ambiguity, letters and reports are not edited. Depending on transfer agreements, referee reports obtained elsewhere may or may not be included in this compilation. Referee reports are anonymous unless the Referee chooses to sign their reports.)

20th Jan 2026

Decision on your manuscript EMM-2026-23327

Dear Dr. Kliewe,

Thank you for submitting your manuscript "Disease-driven post-transcriptional reprogramming of podocytes in focal segmental glomerulosclerosis" to EMBO Molecular Medicine. I have now had a chance to discuss your article with the other members of our editorial team. I am afraid that we concluded that your manuscript is not well suited for publication in EMBO Molecular Medicine and have therefore decided not to proceed with peer review. However, I have taken the liberty to also discuss your study with my colleague Sarita Hebbar - editor at our sister journal Life Science Alliance, which publishes work that is of high value to the respective communities across all areas in the life sciences - and she would be happy to offer peer review if you were to transfer your manuscript there.

From our side, we find that the article doesn't fit well within EMBO Molecular Medicine as we focus primarily on studies that provide functional novel insights of translational significance in an appropriate disease model, that are conceptually novel and of broad interest. As we do not feel that this is the case here, we therefore cannot offer further consideration to your manuscript.

That being said - and as mentioned above - my colleague Sarita would be pleased to send your manuscript out for formal peer-review at Life Science Alliance in its current form. You can take advantage of this offer by transferring your study to Life Science Alliance using the link below; no re-formatting is required.

I am very sorry to disappoint you on this occasion. Please rest assured that this is not a judgment of the quality or interest of your work, but a decision based on the scope requirement of our journal.

Yours sincerely,

Lise Roth

Lise Roth, PhD
Senior Editor
EMBO Molecular Medicine

==========================

As a service to authors, EMBO provides authors with the possibility to transfer a manuscript that one journal cannot offer to publish to another EMBO publication. The full manuscript and if applicable, reviewers reports are automatically sent to the receiving journal to allow for fast handling and a prompt decision on your manuscript. For more details of this service, and to transfer your manuscript to another EMBO title please click on Link Not Available

Please do not share this URL as it will give anyone who clicks it access to your account.

Dear Dr. Roth,

thank you very much for your thoughtful message and for taking the time to discuss our manuscript with the editorial team.

That said, we would like to respectfully express that we find the decision not to proceed with peer review at EMBO Molecular Medicine difficult to fully understand in light of the scope criteria outlined in your letter.

Our study provides, to our knowledge, one of the first systematic and disease-driven analyses of post-transcriptional regulation, specifically alternative splicing, in podocytes during kidney injury and focal segmental glomerulosclerosis. By integrating a podocyte-specific injury model with transcriptomic and splicing-focused analyses, we uncover injury-associated splicing programs that extend beyond descriptive transcriptional changes and point to novel regulatory layers relevant to glomerular disease biology.

We believe these findings address an important and underexplored mechanism in kidney disease and are of direct relevance to the nephrology and molecular medicine communities.

[text removed]

Against this background, we would be grateful for clarification as to why our manuscript was deemed unsuitable for EMBO Molecular Medicine without external peer review, despite addressing a comparable biological question within a different, but equally clinically significant, organ system. We strongly feel that expert assessment through peer review would be the most appropriate way to evaluate the novelty, significance, and translational relevance of our findings.

We fully appreciate the editorial constraints and understand that not all manuscripts can be accommodated. Nevertheless, we kindly ask whether the decision could be reconsidered, or whether you could provide more specific guidance on the aspects that, in

the editors' view, preclude peer review at EMBO Molecular Medicine.

Thank you again for your time and consideration. We look forward to your response.

Yours sincerely,

Felix Kliewe

Dr. rer. nat. Felix Kliewe
Universitätsmedizin Greifswald
Institut für Anatomie und Zellbiologie
Friedrich-Loeffler-Str. 23c
17489 Greifswald
Tel: 03834-86/5315
E-Mail: felix.kliewe@uni-greifswald.de
Internet: http://www.medizin.uni-greifswald.de/anatomie/

Dear Dr. Kliewe,

Thank you for your email requesting clarification of our decision regarding your manuscript. I have read your letter carefully and discussed it with my colleagues. While I appreciate your highlighting the conceptual advance of your work, your letter confirms that we understood the study's key findings.

We recognize that your study provides a systematic analysis of post-transcriptional regulation in podocytes during focal segmental glomerulosclerosis using a previously established disease model, as well as providing cross-species comparisons. We acknowledge the novelty and interest of your findings; however, the translational implications and applications of your work are not further developed here, which detracts from the clinical insight we expect. Furthermore, while we appreciate that the investigation is based on an adequate in vivo disease model, the absence of human sample analysis detracts from the potential of your manuscript to be considered a resource for our journal.

I hope this helps you understand the reasons for our decision better. Please rest assured that we acknowledge the quality of your work, and that our decision is solely related to the scope of our journal.

With kind regards,

Lise Roth

Lise Roth, PhD

Senior Editor

EMBO Molecular Medicine

Dear Dr. Roth,

Thank you very much for your prompt response to our inquiry. We truly appreciate the time and consideration you have given to our work.

I would like to give one comment to your response: Over the past years, we have consistently published that the zebrafish larval model provides a robust and highly translational readout that directly reflects key aspects of the disease phenotype observed in humans and in established mammalian systems, including the mouse. Importantly, we are currently use this model for drug development and compound identification, and the results have already contributed to an ongoing patent application—highlighting not only its scientific relevance but also its strong translational and innovative potential.

We believe that our work is especially timely. The field is rapidly shifting away from traditional mouse models toward more predictive and scalable systems such as organoids and efficient *in vivo* models like zebrafish larvae. In this context, zebrafish-based approaches are increasingly seen as forward-looking and strategically important (for example, in the United States, funding priorities are already moving away from conventional animal models). We therefore strongly believe that our study aligns very well with the future direction of biomedical research and could be of high interest to your readership.

With this in mind, and despite the current decision not to move our manuscript forward to peer review, **I would like to respectfully ask whether you would be willing to reconsider initiating the review process if we include additionally human and/or clinical data to further strengthen and validate our key findings?**

Thank you again for your consideration. I am looking forward to hearing from you.

With kind regards,

Nicole

Prof. Dr. rer. nat. Nicole Endlich

Geschäftsführerin
Institut für Anatomie und Zellbiologie
Universitätsmedizin Greifswald
F. Loefflerstr.23 c

D-17489 Greifswald
Homeoffice +4915141466588

www.medizin.uni-greifswald.de
www.savethekidney.de

Vorstand: Toralf Giebe (Kaufmännischer Vorstand) · Prof. Dr. Karlhans Endlich (Wissenschaftlicher Vorstand)

Prof. Dr. Klaus Hahnenkamp (Stellv. Ärztlicher Vorstand)
Aufsichtsratsvorsitzender: Dr. Tilmann Schweisfurth

SAVE THE KIDNEY
NORDVERBUND NIERE

Dear Dr. Endlich,

Thank you very much for your detailed email and for highlighting the translational value of the zebrafish model.

In answer to your query, we would indeed be willing to reconsider our decision if you were to include human and/or clinical data in your manuscript.

Please note however that, without seeing the data in question, I cannot guarantee that the outcome would be favourable at this stage.

With kind regards,

Lise Roth

Lise Roth, PhD

Senior Editor

EMBO Molecular Medicine

University Medicine Greifswald · Fleischmannstr. 8 · 17475 Greifswald

Department of Anatomy and Cell Biology
Director: Prof. Dr. med. Karlhans Endlich

Prof. Dr. rer. nat. Nicole Endlich
Managing Director
Friedrich-Loefflerstr. 23c
17487 Greifswald
Tel: 03834/865303
nicole.endlich@uni-greifswald.de

Greifswald, 25.03.26

Dear Editors of *EMBO Molecular Medicine*,
Dear Dr. Roth,

Thank you very much for the opportunity to resubmit our manuscript and for your willingness to reconsider our study upon inclusion of human and clinical data.
We greatly appreciate your constructive guidance.

Our manuscript presents a systematic analysis of **post-transcriptional regulation** in **podocytes** in the kidney disease *focal segmental glomerulosclerosis* (FSGS), identifying conserved **alternative splicing** of mRNAs and the downstream **functional consequences of this events**.
Using an established *in vivo* disease model combined with transcriptomic analyses, we uncover a regulatory axis involving SRSF3-dependent splicing and the polarity regulator EPB41L5 that is associated with podocyte injury.

In response to your comments, we have substantially revised the manuscript and incorporated **novel analyses** based on **human patient data** that directly validate the key findings of our study.

UNIVERSITÄTSMEDIZIN GREIFSWALD · KÖRPERSCHAFT DES ÖFFENTLICHEN RECHTS
Prof. Dr. Uwe Reuter (Vorstandsvorsitzender, Ärztlicher Vorstand), · Toralf Giebe (Kaufmännischer Vorstand), · Prof. Dr. Karlhans Endlich (Wissenschaftlicher Vorstand); AUFSICHTSRATSVORSITZENDER: Dr. Tilmann Schweisfurth · Fleischmannstraße 8 · 17475 Greifswald · www.medizin.uni-greifswald.de

First, we have added new analyses of **SRSF3** expression in human glomerular datasets, which are now presented in Figure 6. Here, we observed a pronounced upregulation of SRSF3 in glomeruli from patients with FSGS. This finding provides strong clinical support for the relevance of SRSF3-mediated post-transcriptional regulation in human disease and is consistent with the regulatory role suggested by our experimental analyses.

Second, we included new analyses of **EPB41L5** expression in glomeruli from patients with FSGS, shown in the new Figure 7. Our results reveal a significant downregulation of EPB41L5 in human FSGS glomeruli, providing direct clinical support for the molecular pathways identified in our study.

In addition, we incorporated complementary patient-derived data related to **PLCE1**, further strengthening the link between our mechanistic findings and human disease.

Taken together, these new datasets establish a **clear translational connection** between the mechanistic insights obtained in our model system and molecular signatures observed in patients. We believe that the revised manuscript now addresses the points raised in your previous assessment and substantially strengthens the **clinical relevance of the study**.

We would be grateful if you would consider sending the revised manuscript for external peer review.

Thank you very much for your time and consideration.

Kind regards,

Prof. Dr. Nicole Endlich and Dr. Felix Kliewe

UNIVERSITÄTSMEDIZIN GREIFSWALD · KÖRPERSCHAFT DES ÖFFENTLICHEN RECHTS
Prof. Dr. Uwe Reuter (Vorstandsvorsitzender, Ärztlicher Vorstand), · Toralf Giebe (Kaufmännischer Vorstand), · Prof. Dr. Karlhans Endlich (Wissenschaftlicher Vorstand); AUFSICHTSRATSVORSITZENDER: Dr. Tilmann Schweisfurth · Fleischmannstraße 8 · 17475 Greifswald · www.medizin.uni-greifswald.de

5th May 2026

Dear Dr. Kliewe,

Thank you for submitting your manuscript to our editorial office, and please accept my apologies for the delay in getting back to you as one referee needed more time to complete their report. We have now received feedback from the three referees who evaluated your study. As you will see in the reports below, while they acknowledge the potential interest of the study, they also raise several points that would help clarify the work and strengthen its overall impact.

If you feel you can satisfactorily address the points listed by the referees, you may wish to submit a revised version of your manuscript. Regarding referee #1's comment on the descriptive nature of the study, we believe the manuscript could be considered a Resource and therefore would not require additional functional validation. Please attach a covering letter giving details of the way in which you have handled each of the points raised by the referees. A revised manuscript will once again be subject to review, and we cannot guarantee at this stage that the eventual outcome will be favorable. We expect your revised manuscript within three months. If you anticipate any delay, please contact us.

If you would like to discuss further the reports from the referees, I am available to do so via email or video. Let me know if you are interested in this option.

When preparing your revised manuscript, please refer to our guidelines: https://link.springer.com/journal/44321/submission-guidelines#cms-Revised-submissions. We perform an initial quality control of all revised manuscripts before re-review; failure to include requested items will delay the evaluation of your revision.

We require:

1) A .docx formatted version of the manuscript text (including legends for main figures, EV figures and tables). Please make sure that the changes are highlighted to be clearly visible.

2) Individual production quality figure files as .eps, .tif, .jpg (one file per figure). For guidance, download the 'Figure Guide PDF': https://media.springernature.com/original/springer-cms/rest/v1/content/27825798/data/v1.

3) A .docx formatted letter INCLUDING the reviewers' reports and your detailed point-by-point responses to their comments. As part of the EMBO Press transparent editorial process, the point-by-point response is part of the Review Process File (RPF), which will be published alongside your paper.

4) A complete author checklist, which you can download from our author guidelines. Please insert information in the checklist that is also reflected in the manuscript. The completed author checklist will also be part of the RPF.

5) Please note that all corresponding authors are required to supply an ORCID ID for their name upon submission of a revised manuscript.

6) It is mandatory to include a 'Data Availability' section after the Materials and Methods. Before submitting your revision, primary datasets produced in this study need to be deposited in an appropriate public database, and the accession numbers and database listed under 'Data Availability'. Please remember to provide a reviewer password if the datasets are not yet public.

In case you have no data that requires deposition in a public database, please state so in this section. Note that the Data Availability Section is restricted to new primary data that are part of this study.

7) For data quantification: please specify the name of the statistical test used to generate error bars and P values, the number (n) of independent experiments (specify technical or biological replicates) underlying each data point and the test used to calculate p-values in each figure legend. The figure legends should contain a basic description of n, P and the test applied. Graphs must include a description of the bars and the error bars (s.d., s.e.m.).

8) At EMBO Press we ask authors to provide source data for the main manuscript figures. You will receive a separate email with instructions for providing source data with your revised manuscript, including how to upload and organize the files.

9) Our journal encourages inclusion of *data citations in the reference list* to directly cite datasets that were re-used and obtained from public databases. Data citations in the article text are distinct from normal bibliographical citations and should directly link to the database records from which the data can be accessed. In the main text, data citations are formatted as follows: "Data ref: Smith et al, 2001" or "Data ref: NCBI Sequence Read Archive PRJNA342805, 2017". In the Reference list, data citations must be labeled with "[DATASET]". A data reference must provide the database name, accession number/identifiers and a resolvable link to the landing page from which the data can be accessed at the end of the reference.

10) We replaced Supplementary Information with Expanded View (EV) Figures and Tables that are collapsible/expandable online. A maximum of 5 EV Figures can be typeset. EV Figures should be cited as 'Figure EV1, Figure EV2" etc... in the text and their respective legends should be included in the main text after the legends of regular figures.

- For the figures that you do NOT wish to display as Expanded View figures, they should be bundled together with their legends in a single PDF file called *Appendix*, which should start with a short Table of Content. Appendix figures should be referred to in the main text as: "Appendix Figure S1, Appendix Figure S2" etc.

- Additional Tables/Datasets should be labeled and referred to as Table EV1, Dataset EV1, etc. Legends have to be provided in a separate tab in case of .xls files. Alternatively, the legend can be supplied as a separate text file (README) and zipped together with the Table/Dataset file.

11) The paper explained: EMBO Molecular Medicine articles are accompanied by a summary of the articles to emphasize the major findings in the paper and their medical implications for the non-specialist reader. Please provide a draft summary of your article highlighting
- the medical issue you are addressing,
- the results obtained and
- their clinical impact.

This may be edited to ensure that readers understand the significance and context of the research. Please refer to any of our published articles for an example.

12) Author contributions: You will be asked to provide CRediT (Contributor Role Taxonomy) terms in the submission system. These replace a narrative author contribution section in the manuscript.

13) A "Disclosure and Competing Interests Statement" should be provided in the main text.

14) Every published paper includes a 'Synopsis' to further enhance discoverability. Synopses are displayed on the journal webpage and are freely accessible to all readers. They include a short stand first (maximum of 300 characters, including space) as well as 2-5 one-sentences bullet points that summarizes the paper. Please write the bullet points to summarize the key NEW findings. They should be designed to be complementary to the abstract - i.e. not repeat the same text. We encourage inclusion of key acronyms and quantitative information (maximum of 30 words / bullet point). Please use the passive voice. Please attach these in a separate file or send them by email, we will incorporate them accordingly.

Please also suggest a striking image or visual abstract to illustrate your article as a PNG file 550 px wide x 300 px high.

15) Include a Reagents and Tools Table as part of the Methods section, which can be downloaded from our author guidelines.

16) As part of the EMBO Publications transparent editorial process initiative (see our Editorial at http://embomolmed.embopress.org/content/2/9/329), EMBO Molecular Medicine will publish online a Review Process File (RPF) to accompany accepted manuscripts.
In the event of acceptance, this file will be published in conjunction with your paper and will include the anonymous referee reports, your point-by-point response and all pertinent correspondence relating to the manuscript. Let us know whether you agree with the publication of the RPF and as here, if you want to remove or not any figures from it prior to publication.
Please note that the Authors checklist will be published at the end of the RPF.

EMBO Molecular Medicine has a "scooping protection" policy, whereby similar findings that are published by others during review or revision are not a criterion for rejection. Should you decide to submit a revised version, I do ask that you get in touch after three months if you have not completed it, to update us on the status.

I look forward to receiving your revised manuscript.

Yours sincerely,

Lise Roth

Lise Roth, PhD
Senior Editor
EMBO Molecular Medicine

***** Reviewer's comments *****

Referee #1 (Comments on Novelty/Model System for Author):

This is a strong and timely manuscript that provides new insight into post-transcriptional regulatory mechanisms in FSGS, particularly alternative splicing in podocytes. The data are comprehensive, the analyses are appropriate, and the cross-species validation enhances the impact of the work. Nevertheless the article is mostly descriptive. Some functional experiments would increase the medical impact.

Referee #1 (Remarks for Author):

In this manuscript, the authors investigate disease-driven post-transcriptional alterations in podocytes during focal segmental glomerulosclerosis (FSGS), with a particular focus on microRNA regulation and alternative splicing. By combining a podocyte-specific injury model in zebrafish with bulk RNA sequencing, small RNA profiling, alternative splicing analyses, and cross-species validation in human FSGS samples, the study provides a comprehensive and technically solid dataset. Overall, the work is methodologically robust, the experimental design is appropriate, and the conclusions are generally well supported by the data. The integration of zebrafish, mouse, and human datasets strengthens the translational relevance of the findings. The identification of disease-associated isoform switches, particularly involving SRSF3 and EPB41L5, represents a novel and interesting contribution to the understanding of podocyte injury in FSGS.
Major points

- While the RNA-seq and miRNA analyses appear technically sound, it is not always immediately clear how many independent biological replicates were used for each condition, particularly for isolated glomeruli at each time point. Please explicitly state the number of independent biological replicates for each RNA-seq and miRNA-seq experiment in the Results section and/or figure legends. This is particularly important for Figures 2 to 5, where conclusions rely on differential expression and splicing analyses.
- The negative results obtained with JAK/STAT3 inhibitors are interesting and potentially important. However, the interpretation remains somewhat brief. Please clarify whether the lack of protection could be related to timing, dosage, or pathway redundancy, as suggested in previous studies on STAT3 signaling in glomerular disease. An extended discussion placing these findings in the context of existing literature (e.g. STAT3 activation reported in human FSGS biopsies) would be necessary, even if key articles are cited.
Minor points
- The title accurately reflects the topic but could be made slightly more specific by explicitly mentioning alternative splicing or isoform switching, which is a central and novel aspect of the study.
- The abstract should state that the study has been performed on a zebra fish model.
- The identification of miR-21 and miR-193 target genes is convincing at the transcriptomic level. Please clarify in the Methods or Results whether target identification is exclusively bioinformatic or whether any experimental validation is available or planned.
- Please double check for isoform nomenclature and consistency
throughout the Results and figure legends to improve readability for non-specialists.
- The NFP-based injury model and transgenic zebra fish should be briefly recalled/described in the Results section. This would not be necessary if the methods section were placed before the results.
- Please explain the meaning of "significantly stronger expression pattern" (isolated glomeruli vs whole larvae) or rephrase (Figure 3A).
- How are "genes associated with FSGS" defined? (figure 3H)
- On page 5, the sentence citing human microarray data appears unexpectedly. In general, the comparison with existing databases should be properly introduced in the Results, and also mentioned in the Methods.
- The figures are of high quality, but some panels (especially in the supplementary figures related to splicing analyses) are dense. Consider adding brief explanatory sentences in the figure legends to guide the reader.
- In Figures 2, 3 and 4, it is confusing the differences in number of genes between volcano plots and venn diagrams. This refers to the previous comment.
- Most of the figures need an increase in the font size
- Figure 6C: please explain better the difference between "isoform expression" and "isoform fraction".
- Some information is needed about the individuals at the origin of kidney biopsies. To be included in the Methods (at least how many individuals, age, gender, and diagnosis).

Referee #2 (Comments on Novelty/Model System for Author):

The authors should use results from other disease systems to validate if the events noted in this manuscript are specific to FSGS.

Referee #2 (Remarks for Author):

Summary:
This study by Mattias Et Al., investigates post-transcriptional alterations of podocytes in FSGS using zebrafish injury as a model system. While the investigator has done a phenomenal amount of work, which is commendable, the manuscript fails to highlight the key events that are critical for FSGS progression. There is a lack of clear hypothesis. Many of the pathways and remodeling events are in response to injury, but whether these are the primary or secondary to injury is not clear. While the comparative analysis of zebrafish events with humans is complementary, how and what knowledge gaps zebrafish model will address with respect to FSGS remains unclear.

Major comments
1) Although the investigator has previously published the zebrafish FSGS model of injury, they need to demonstrate that the injury to zebrafish podocytes is consistent with the timeline used in this study for 5 & 6 dpf.
2) Comparing zebrafish results with Human data is a big leap. There should be a comparison with equivalent mouse model/s to determine the utility of these zebrafish data.
3) It is not clear which event is primary or critical for FSGS progression, changes in STAT3 signaling, miRNA or alternate splicing. The investigator has not made his hypothesis clear.
4) The study looks like a comparison between zebrafish and human glomeruli and characterizing the differences and similarities in the miRNA and genetic profile. The discussion on how this model will promote novel discoveries and mechanistic insight into FSGS is lacking.
5) What is the novelty remain unclear. All genes and splicing events identified have been prevalent in human FSGS models. Emphasis should be given to how these events contribute to FSGS and whether these can be targeted to prevent FSGS.
6) The authors claim alternative splicing as the novel and key finding specific to FSGS. However, it may be an event that is also prevalent in other diseases or kidney injuries. The authors fail to show that it is specific to FSGS. This is counter to their claim that these events can lead to biomarker discoveries. The authors need to provide additional data to show these are specific events to FSGS and may lead to biomarker discovery.
Minor Comments:
1. It is not clear from Fig 6H and I which nuclei we are looking at. Please provide zoom in and zoom out images to demonstrate that these are podocyte nuclei.

Referee #3 (Comments on Novelty/Model System for Author):

The model is reasonable, especially based on the fact that the authors establish concordance in expression changes across zebrafish (the model), mouse, and human.

Referee #3 (Remarks for Author):

This study investigates transcriptomic changes associated with glomerular injury in zebrafish, using RNA-seq from isolated glomeruli and whole larvae at 5 and 6 days post fertilization. The analyses appear technically sound, and a major strength is the consistent integration of findings across zebrafish, mouse, and human expression datasets. In particular, the emphasis on splicing changes that occur in the absence of overall gene-level differential expression highlights an important and often underappreciated regulatory layer in injury responses.
I have a small number of questions and requests for clarification that would strengthen the manuscript and improve interpretability.

In Figures 1C, 2C, and 2H, the numbers of differentially expressed genes do not appear to match the DEG counts described in the text. Please clarify whether these discrepancies arise from differences in statistical thresholds, filtering criteria, or gene set definitions, and indicate this explicitly in the figure legends or methods.

It is unclear whether Figure 4B is based on the isolated glomeruli dataset or the whole-larvae dataset. This should be stated clearly in the panel label or legend, as the biological interpretation depends on the tissue context.

The datasets shown in Figure S7 require clearer labeling. As currently presented, the reader has to infer sample identity based on other figures. Each panel should explicitly state which dataset, condition, and developmental stage are being shown.

Please comment on whether larval sex is expected to influence the injury phenotype or transcriptomic response. If sex differences are relevant at this stage, sexing the already analyzed samples and reporting this information would provide useful biological context. If sex is not expected to matter, a brief rationale would be helpful.
Overall, the study is compelling, and addressing these points would improve clarity and rigor without requiring substantial additional analyses.

Point-by-Point Response to Reviewers

Manuscript EMM-2026-23327-V2-Q

"Disease-driven post-transcriptional alterations of podocytes in focal segmental glomerulosclerosis"

We thank the Editor and all three Reviewers for their careful reading of our manuscript and for their constructive and thoughtful comments. We are pleased that all reviewers acknowledged the comprehensiveness and translational relevance of our study. We have addressed all points raised by careful revision of the text, extended discussion, clarification of methods, and improved figure presentation. All changes are highlighted in the revised manuscript. Our detailed point-by-point responses are provided below.

Referee #1 (Comments on Novelty/Model System for Author):

This is a strong and timely manuscript that provides new insight into post-transcriptional regulatory mechanisms in FSGS, particularly alternative splicing in podocytes. The data are comprehensive, the analyses are appropriate, and the cross-species validation enhances the impact of the work. Nevertheless, the article is mostly descriptive. Some functional experiments would increase the medical impact.

We sincerely thank Referee #1 for the highly positive assessment and for recognizing the novelty of the isoform switch findings and the translational value of the cross-species validation. We appreciate the comment regarding the descriptive nature of the study and would like to respectfully highlight several aspects that we believe go beyond a purely descriptive analysis:

First, the identification of a disease-associated isoform switch in SRSF3, a master splicing regulator, provides a mechanistic link between podocyte injury and widespread splicing dysregulation, positioning SRSF3 as a potential regulatory hub.

Second, the coordinated dysregulation of the EPB41L5/CRB2/MPP5 polarity complex, including the isoform switch in EPB41L5, connects our molecular findings directly to established functional consequences in podocyte biology, namely foot process effacement and proteinuria.

Third, the cross-species validation of transcriptional changes identified in the zebrafish injury model against human FSGS glomerular datasets and mouse glomerular injury models demonstrates that our findings reflect conserved, disease-relevant molecular events.

Finally, we have revised and expanded the Discussion to more prominently highlight the mechanistic implications of our findings, including specific functional hypotheses and concrete directions for future experimental follow-up, so that this dataset can serve as a starting point for targeted mechanistic studies by us and the broader community.

Referee #1 (Remarks for Author):

In this manuscript, the authors investigate disease-driven post-transcriptional alterations in podocytes during focal segmental glomerulosclerosis (FSGS), with a particular focus on microRNA regulation and alternative splicing. By combining a podocyte-specific injury model in zebrafish with bulk RNA sequencing, small RNA profiling, alternative splicing analyses, and cross-species validation in human FSGS samples, the study provides a comprehensive and technically solid dataset. Overall, the work is methodologically robust, the experimental design is appropriate, and the conclusions are generally well supported by the data. The integration of zebrafish, mouse, and human datasets strengthens the translational relevance of the findings. The identification of disease-associated isoform switches, particularly involving SRSF3 and EPB41L5, represents a novel and interesting contribution to the understanding of podocyte injury in FSGS.

We thank the reviewer for his positive evaluation of our manuscript and for his constructive comments. We have revised the manuscript accordingly and addressed all points raised.

Major points

1.) While the RNA-seq and miRNA analyses appear technically sound, it is not always immediately clear how many independent biological replicates were used for each condition, particularly for isolated glomeruli at each time point. Please explicitly state the number of independent biological replicates for each RNA-seq and miRNA-seq experiment in the Results section and/or figure legends. This is particularly important for Figures 2 to 5, where conclusions rely on differential expression and splicing analyses.

The reviewer raised an important point, and we agree that clearly reporting the number of replicates is essential for transparency and reproducibility. Therefore, we have now included the number of independent biological replicates for all RNA-seq and miRNA-seq experiments in both the Results section and the corresponding figure legends of Figures 2-5. Specifically, all RNA-seq experiments were performed with n=3 independent biological replicates per condition at each time point (5 and 6 dpf), for both whole larvae and isolated glomeruli.

The miRNA sequencing was likewise performed with n=3 biological replicates per group. This information is now included in the revised figure legends for Figures 2, 3, 4, and 5, as well as in the Results section.

2.) The negative results obtained with JAK/STAT3 inhibitors are interesting and potentially important. However, the interpretation remains somewhat brief. Please clarify whether the lack of protection could be related to timing, dosage, or pathway redundancy, as suggested in previous studies on STAT3 signaling in glomerular disease. An extended discussion placing these findings in the

context of existing literature (e.g. STAT3 activation reported in human FSGS biopsies) would be necessary, even if key articles are cited.

We fully agree that this negative finding should be more discussed. We have expanded the Discussion to discuss potential reasons for the lack of protection. Specifically, we now included three non-mutually exclusive explanations:

(1) Timing and injury severity: Although the inhibitors were administered concomitantly with NFP, the severe podocyte depletion induced by 50 nM NFP cannot be rescued by the drug. Given the postmitotic nature of podocytes, irreversible cell loss may proceed faster than pathway blockade can mitigate.

(2) Drug delivery: While the doses used were carefully titrated to avoid direct toxicity (as evidenced by the DMSO vehicle controls), bioavailability within glomerular tissue in zebrafish larvae may differ from *in vitro* predictions. The accelerated disease progression observed at higher inhibitor concentrations suggests dose-dependent toxicity, which precluded increasing the dose to potentially protective levels.

(3) Pathway redundancy: STAT3 activation likely represents one branch of a broader inflammatory response, and compensatory activation of parallel pathways (e.g., NF-κB, mTOR, TGF-β) may sustain disease progression even under pharmacological JAK/STAT3 blockade, consistent with published observations in multi-factorial glomerular disease models (Tao et al., 2018; Henique et al., 2017). We now explicitly place our findings in the context of published reports documenting STAT3 activation in human FSGS biopsies (Tao et al., 2018) and mouse models (Henique et al., 2017).

Minor points

1) The title accurately reflects the topic but could be made slightly more specific by explicitly mentioning alternative splicing or isoform switching, which is a central and novel aspect of the study. The abstract should state that the study has been performed on a zebra fish model.

We thank the reviewer for this suggestion. We agree that the title should better reflect the novel contribution of our work. We have revised the title to: *"Disease-driven post-transcriptional alterations and alternative splicing of podocytes in focal segmental glomerulosclerosis."* In addition, we have revised the Abstract to explicitly state that the study was performed using a zebrafish model of FSGS. The revised abstract now reads: "Here, we combined a *zebrafish* podocyte-specific injury model with glomerulus-resolved transcriptomic profiling to dissect RNA regulatory alterations during FSGS progression."

2) The identification of miR-21 and miR-193 target genes is convincing at the transcriptomic level. Please clarify in the Methods or Results whether target identification is exclusively bioinformatic or whether any experimental validation is available or planned.

The reviewer is right. We have now added a clear statement in both the Methods and Results sections specifying that target gene identification for miR-21 and miR-193 is **exclusively bioinformatic** in the present study. Putative targets of miR-21 were obtained from miRDB (Chen & Wang, 2020) and targets of miR-193 were derived from the experimentally validated list published by Gebeshuber et al. (2013). The overlap of these predicted/validated targets with differentially expressed genes (DEGs) in our dataset was then used to define putative regulatory relationships. No direct experimental validation (e.g., luciferase reporter assays) was performed in the present study; this limitation is now explicitly stated in the Discussion, and experimental validation of specific miRNA-target interactions is noted as an important objective for future mechanistic studies.

3) Please double check for isoform nomenclature and consistency throughout the Results and figure legends to improve readability for non-specialists.

We have carefully reviewed all isoform designations throughout the manuscript, Results section, and figure legends and have standardized nomenclature throughout. All isoform names now consistently follow Ensembl nomenclature (based on release 113, October 2024) and are presented uniformly in italics for zebrafish gene/isoform names and in italicized uppercase for human orthologs. We have also ensured that each isoform is defined upon first use in each figure legend to aid readability for non-specialists.

4) The NFP-based injury model and transgenic zebra fish should be briefly recalled/described in the Results section. This would not be necessary if the methods section were placed before the results.

We thank the reviewer for this practical suggestion. We have added a brief introductory paragraph at the beginning of the Results section (before the first subsection) that concisely summarizes the Nifurpirinol (NFP)-based podocyte injury model and the transgenic zebrafish line used (*Tg(nphs2:GAL4-VP16); Tg(UAS:Eco.nfsB-mCherry)*). This paragraph directs the reader to the full methodological details provided in the Methods section, thereby ensuring that the Results are self-contained and accessible without requiring prior knowledge of the model system.

5) Please explain the meaning of "significantly stronger expression pattern" (isolated glomeruli vs whole larvae) or rephrase (Figure 3A).

We agree that this phrasing was imprecise and potentially confusing. We have revised the relevant sentence in the Results section to read: "*In contrast to whole larvae, isolated glomeruli showed a substantially* ***higher number of differentially expressed genes*** *(1,007 DEGs at 5 dpf and 2,299 DEGs at 6 dpf), likely reflecting the enrichment of glomerulus-specific transcriptomic changes following isolation from surrounding tissue.*" This revision clarifies that "stronger" refers to the greater statistical power and

specificity of the glomerular dataset due to tissue enrichment, not to higher individual gene expression levels.

6) How are "genes associated with FSGS" defined? (figure 3H)

We have clarified this point in the manuscript. The figure legend of Figure 3H and the corresponding text in the Results now define the term: "Genes associated with FSGS" in this context refers to "**differentially expressed genes (DEGs) identified in human FSGS glomeruli"** from the publicly available Nephroseq dataset (Hodgin FSGS Glom Dataset; Hodgin et al., 2010), applying a significance cutoff of adjusted p-value ≤ 0.05. The Venn diagram in Figure 3H thus depicts the overlap between these human FSGS-associated DEGs and the DEGs identified in our zebrafish FSGS model.

7) On page 5, the sentence citing human microarray data appears unexpectedly. In general, the comparison with existing databases should be properly introduced in the Results, and also mentioned in the Methods.

The reviewer is right. We have revised the Results section to properly introduce the use of publicly available human glomerular expression datasets (Nephroseq: Hodgin FSGS Glom Dataset, Lindenmeyer Normal Tissue Panel) prior to their first citation. A corresponding subsection in the Methods entitled "Analysis of publicly available human transcriptomic data" has been added, which describes the databases accessed, the datasets used (with accession/reference information), and the analytical approach applied. All subsequent references to human microarray or RNA-seq data in the Results are now accompanied by an explicit mention of the data source.

8) The figures are of high quality, but some panels (especially in the supplementary figures related to splicing analyses) are dense. Consider adding brief explanatory sentences in the figure legends to guide the reader.

We fully agree with this comment. We have revised the legends for Supplementary Figures S4, S5, and S6, which depict the alternative splicing Venn diagrams and single-cell expression heatmaps, to include brief explanatory sentences that guide the reader through the structure and interpretation of each panel. We have also added a short explanatory note at the beginning of each supplementary splicing figure clarifying the column annotations for the kidney cell type expression.

9) In Figures 2, 3 and 4, it is confusing the differences in number of genes between volcano plots and venn diagrams. This refers to the previous comment.

The reviewer is right. The discrepancy arises from different filtering criteria used for the volcano plots and Venn diagrams. Specifically, the volcano plots display genes passing both the adjusted p-value threshold (adjusted p-value ≤ 0.05) and the fold-change threshold (log2FC ≥ 1). In contrast, the Venn diagrams include all significantly regulated genes based solely on statistical significance (adjusted p-value ≤ 0.05),

without applying an additional log2 fold-change cutoff. As a result, the number of genes shown in the Venn diagrams is larger.

We have now added an explicit explanatory note to the legends of Figures 2, 3, and 4 to clarify these differences in filtering criteria.

10) Most of the figures need an increase in the font size

We have revised all main figures and relevant supplementary figures to increase the font size of axis labels, line marks, gene names, and panel labels to improve readability. All revised figures are provided as high-resolution quality files with the revised manuscript submission.

11) Figure 6C: please explain better the difference between "isoform expression" and "isoform fraction".

"Isoform expression" (shown in Figure 6B) refers to the *absolute normalized read counts* (DESeq2-normalized gene counts) for each individual transcript isoform, reflecting the total abundance of that isoform in the sample. In contrast, **"Isoform fraction"** (Figure 6C) refers to the *relative proportion* of each isoform's expression as a fraction of the total gene expression (i.e., isoform count / sum of all isoform counts for that gene). The isoform fraction thus captures shifts in isoform usage independently of overall changes in gene expression level, which is the key metric for identifying isoform switches. We have added this explanation as a methodological note in the figure legend for Figure 6 and in the IsoformSwitchAnalyzeR section of the Methods.

12) Some information is needed about the individuals at the origin of kidney biopsies. To be included in the Methods (at least how many individuals, age, gender, and diagnosis).

This is an important point. We have expanded the existing “Histology on human kidney biopsies” section in the Methods to clarify the composition of the biopsy cohorts used in this study. Specifically, we now state the number of individuals included per cohort and clarify that all diagnoses, including FSGS, MCD, and MGN, were established by experienced renal pathologists based on routine histopathological evaluation.

Due to the approved ethical disclaimer for the use of archived and fully anonymized remnant biopsy material, detailed patient-specific clinical information, including age and sex, was not available to the authors.

Referee #2 (Comments on Novelty/Model System for Author):

The authors should use results from other disease systems to validate if the events noted in this manuscript are specific to FSGS.

Referee #2 (Remarks for Author):

Summary:
This study by Mattias Et Al., investigates post-transcriptional alterations of podocytes in FSGS using zebrafish injury as a model system. While the investigator has done a phenomenal amount of work, which is commendable, the manuscript fails to highlight the key events that are critical for FSGS progression. There is a lack of clear hypotheses. Many of the pathways and remodeling events are in response to injury, but whether these are the primary or secondary to injury is not clear. While the comparative analysis of zebrafish events with humans is complementary, how and what knowledge gaps zebrafish model will address with respect to FSGS remains unclear.

We would like to thank Reviewer No. 2 for acknowledging the considerable amount of work involved. We have carefully addressed all concerns through text revisions, clarification, and extended discussion, referencing existing data within the manuscript wherever possible. We respond to each point in detail below.

Major comments

1) Although the investigator has previously published the zebrafish FSGS model of injury, they need to demonstrate that the injury to zebrafish podocytes is consistent with the timeline used in this study for 5 & 6 dpf.

We appreciate this comment and understand the need for clarity regarding the consistency of injury at the time points analyzed. We would like to point out that the Nifurpirinol (NFP)-based podocyte injury model used in this study has been extensively characterized and validated in our previous publications (Klawitter et al., 2024; Schindler et al., 2023), which are cited throughout the manuscript and describe the morphological, functional, and molecular features of injury at 5 and 6 dpf in detail. Nevertheless, to address this concern directly within the present manuscript, we have added a dedicated paragraph in the Results section describing the injury phenotype observed at both time points, explicitly referencing: (1) Figure 1B, which shows the development of periocular and abdominal edema at 5 and 6 dpf in NFP-treated but not DMSO-treated larvae; (2) the reduction in mCherry fluorescence in podocytes, indicative of podocyte loss; and (3) the clear principal component separation between NFP and DMSO groups (Figure 1C), demonstrating consistent and reproducible transcriptomic differences at both time points. These features collectively confirm that the injury phenotype is reproducible and progressive between 5 and 6 dpf, fully consistent with the timeline used for all downstream analyses.

2) Comparing zebrafish results with Human data is a big leap. There should be a comparison with equivalent mouse model/s to determine the utility of these zebrafish data.

We respectfully draw the reviewer's attention to the fact that we have already integrated mouse model data extensively throughout our manuscript, specifically using published glomerular single-cell RNA-seq data from the study by Chung et al. (2020, *J Am Soc Nephrol*) covering two independent mouse FSGS models: nephrotoxic serum nephritis (NTS) and doxorubicin nephropathy.

This comparison is presented in:

- Figure 4E: miR-193 target gene expression in mouse podocyte clusters
- Figures 7F–H: Podocyte-specific expression of EPB41L5, CRB2, and MPP5 in NTS and doxorubicin mouse models
- Figure EV3: STAT3, SOCS3, and PTPRO expression in mouse podocytes after injury
- Figures S5–S6: Single-cell expression heatmaps of alternatively spliced candidates in mouse kidney

To improve clarity, we have revised the Results and Discussion sections to more explicitly emphasize these mouse validations and their relevance for supporting the translational utility of the zebrafish model.

3) It is not clear which event is primary or critical for FSGS progression, changes in STAT3 signaling, miRNA or alternate splicing. The investigator has not made his hypothesis clear.

We appreciate this important conceptual comment. The central aim of this study was to comprehensively map the landscape of post-transcriptional regulatory events during FSGS progression, rather than to establish the primacy of any single pathway. We fully acknowledge the reviewer's point that clearer framing of the biological narrative is needed. We have revised the Introduction to more explicitly state our overarching hypothesis: that post-transcriptional mechanisms, including miRNA regulation and alternative splicing, constitute a major and underappreciated regulatory layer in FSGS pathogenesis, acting in concert with but downstream of transcriptional changes.

We have also clarified in the Discussion that the data do not allow us to definitively distinguish primary from secondary events, but that the time-dependent progression of both miRNA dysregulation (predominantly present at both time points) and alternative splicing (markedly amplified between 5 and 6 dpf) suggests that these may represent early and late regulatory responses, respectively.

The isoform switch in SRSF3, occurring simultaneously with the increase in alternative splicing events, is consistent with the concept of a *regulatory hub* that may amplify

splicing perturbations during disease progression. These interpretations are now explicitly stated in the Discussion.

4) The study looks like a comparison between zebrafish and human glomeruli and characterizing the differences and similarities in the miRNA and genetic profile. The discussion on how this model will promote novel discoveries and mechanistic insight into FSGS is lacking.

We appreciate this critical assessment. We agree that the Discussion could more forcefully articulate the mechanistic and translational implications of our findings. We have substantially revised the Discussion to address this, specifically by adding paragraphs that:

1. Discuss the mechanistic consequences of the identified isoform switches: e.g., how loss of the serine-rich C-terminal domain of SRSF3 (isoform 206) may alter phosphorylation-dependent nucleocytoplasmic shuttling and thereby globally disrupt splicing fidelity in podocytes. This positions SRSF3 isoform switching not merely as a descriptive observation but as a potential amplifier of widespread splicing dysregulation.

2. Discuss the functional implications of EPB41L5 isoform switching and the coordinated downregulation of the CRB2/MPP5/EPB41L5 polarity complex, connecting these observations to the known consequences of Crumbs complex disruption (proteinuria, foot process effacement) in FSGS.

3. Articulate how the zebrafish model system, by isolation of pure glomerular tissue for multi-omics profiling, provides unique access to glomerulus-specific post-transcriptional regulation that is difficult to resolve in bulk human biopsy material.

5) What is the novelty remain unclear. All genes and splicing events identified have been prevalent in human FSGS models. Emphasis should be given to how these events contribute to FSGS and whether these can be targeted to prevent FSGS.

We respectfully note that, while individual genes such as NPHS1, NPHS2, or WT1 are well-established FSGS markers, the **post-transcriptional regulatory landscape** (encompassing miRNA regulation and, most importantly, alternative splicing) **has <u>not</u> been systematically profiled in FSGS at the glomerular level in a time-resolved manner.**

The novelty of our study lies in:

1. The first systematic, multi-tool analysis of alternative splicing events in FSGS at the glomerular level, revealing that splicing changes affect a broad set of podocyte-specific structural genes that are not detectably regulated at the gene expression level alone.

2. The identification of a disease-associated isoform switch in SRSF3, a master splicing regulator, in podocytes, which has not been previously described in FSGS and provides a mechanistic link between podocyte injury and global splicing dysregulation.

3. The cross-species validation of FSGS-associated isoform switches (SRSF3, EPB41L5) in human FSGS glomeruli, demonstrating for the first time that isoform-level changes in these genes occur in human disease.

We have revised the final paragraph of the Discussion to more prominently articulate these novel contributions and to frame them explicitly as the basis for future therapeutic targeting strategies. Specifically, we now discuss the **potential of splicing-corrective approaches** (e.g., splice-switching antisense oligonucleotides) and isoform-specific biomarkers as emerging directions building directly from this Resource.

6) The authors claim alternative splicing as the novel and key finding specific to FSGS. However, it may be an event that is also prevalent in other diseases or kidney injuries. The authors fail to show that it is specific to FSGS. This is counter to their claim that these events can lead to biomarker discoveries. The authors need to provide additional data to show these are specific events to FSGS and may lead to biomarker discovery.

This is an important conceptual point and we appreciate the reviewer's comment. We fully agree that alternative splicing is a general cellular response that is not inherently disease-specific, and we have revised the manuscript to avoid any overstatement in this regard. Indeed, we do not intend to suggest that alternative splicing per se is specific to FSGS. Rather, we show that specific isoform switches in podocyte-enriched genes, such as *SRSF3* and *EPB41L5*, occur during FSGS development in our zebrafish FSGS model and are also observed in human FSGS glomeruli. We validated EPB41L5 protein expression by immunofluorescence in human biopsies and demonstrated that the reduction is specific to FSGS compared with both healthy controls and membranous glomerulopathy (MGN) (Figure 7L, M), which represents a degree of disease specificity. Moreover, comparing splicing events from our model with the mouse NTS and doxorubicin models (Chung et al., 2020) allows us to identify changes shared across multiple injury paradigms (reflecting general injury responses) versus those unique to the podocyte depletion context.

We have revised the Discussion to explicitly acknowledge the limitation of disease specificity for global splicing changes and to show that isoform switches in SRSF3 and EPB41L5 are the best candidates for a disease-specific biomarker development. We also acknowledge that prospective studies comparing FSGS with other glomerular diseases will be necessary to formally establish isoform-level biomarker specificity.

Minor Comments:

1) It is not clear from Fig 6H and I which nuclei we are looking at. Please provide zoom in and zoom out images to demonstrate that these are podocyte nuclei.

We thank the reviewer for this important comment on the immunofluorescence data. We have revised Figure 6H and Fig. EV3 to include both a low-magnification overview of the entire glomerulus (showing SRSF3 staining in the context of the overall glomerular architecture) and high-magnification insets that demonstrate co-localization of SRSF3 nuclear signal with synaptopodin-positive podocyte cell bodies. The use of synaptopodin (a well-established podocyte marker) in the adjacent channel allows unambiguous identification of the podocyte-associated nuclei displaying elevated SRSF3 signal. The quantification in Figure 6I, which is based on all DAPI-positive nuclei within the glomerular area, is also noted in the legend, and the podocyte-specific nuclear signal is specifically highlighted in the insets.

Referee #3 (Comments on Novelty/Model System for Author):

The model is reasonable, especially based on the fact that the authors establish concordance in expression changes across zebrafish (the model), mouse, and human.

We thank Referee #3 for the positive overall assessment and for recognizing the technical soundness of our analyses and the strength of the cross-species integration. We address each of the specific requests below.

Referee #3 (Remarks for Author):

This study investigates transcriptomic changes associated with glomerular injury in zebrafish, using RNA-seq from isolated glomeruli and whole larvae at 5 and 6 days post fertilization. The analyses appear technically sound, and a major strength is the consistent integration of findings across zebrafish, mouse, and human expression datasets. In particular, the emphasis on splicing changes that occur in the absence of overall gene-level differential expression highlights an important and often underappreciated regulatory layer in injury responses.
I have a small number of questions and requests for clarification that would strengthen the manuscript and improve interpretability.

We would like to thank the reviewer for the positive feedback.

1) In Figures 1C, 2C, and 2H, the numbers of differentially expressed genes do not appear to match the DEG counts described in the text. Please clarify whether these discrepancies arise from differences in statistical thresholds, filtering criteria, or gene set definitions, and indicate this explicitly in the figure legends or methods.

The discrepancy arises from different filtering criteria used for the volcano plots and Venn diagrams. Specifically, the volcano plots display genes passing both the adjusted p-value threshold (adjusted p-value ≤ 0.05) and the fold-change threshold ($log2FC \geq 1$). In contrast, the Venn diagrams include all significantly regulated genes based solely on statistical significance (adjusted p-value ≤ 0.05), without applying an additional log2 fold-change cutoff. As a result, the number of genes shown in the Venn diagrams is larger.
We have now added an explicit explanatory note to the legends of Figures 2, 3, and 4 to clarify these differences in filtering criteria.

2) It is unclear whether Figure 4B is based on the isolated glomeruli dataset or the whole-larvae dataset. This should be stated clearly in the panel label or legend, as the biological interpretation depends on the tissue context.

The reviewer is right that there is a lack of clarity. Figure 4B is based on the isolated glomeruli dataset (after podocyte-specific glomerular injury compared to control groups). This is now explicitly stated in the panel label and figure legend. We have also confirmed that all other panels in Figure 4 are consistently derived from the glomerular dataset and have standardized this information across all panel labels.

3) The datasets shown in Figure S7 require clearer labeling. As currently presented, the reader has to infer sample identity based on other figures. Each panel should explicitly state which dataset, condition, and developmental stage are being shown.

We agree entirely. We have revised Figure S7 to include explicit labels on each panel stating: (1) the dataset source (single-cell RNA-seq from Ransick et al., 2019, or Chung et al., 2020), (2) the developmental stage or condition compared, and (3) the specific gene list displayed (e.g., "Alternatively spliced genes at 5 dpf glomeruli by rMATS, LeafCutter, and Whippet"). Panel titles and sub-headers have been added to make each panel self-explanatory without cross-reference to other figures. The column label key (cell types 1–32) is now reproduced as a compact legend directly beneath the heatmap.

4) Please comment on whether larval sex is expected to influence the injury phenotype or transcriptomic response. If sex differences are relevant at this stage, sexing the already analyzed samples and reporting this information would provide useful biological context. If sex is not expected to matter, a brief rationale would be helpful. Overall, the study is compelling, and addressing these points would improve clarity and rigor without requiring substantial additional analyses.

This is an excellent point. At the developmental stages used in this study (4–6 dpf), **sex does not influence the injury phenotype or the transcriptomic response** in zebrafish larvae. In zebrafish, all larvae initially develop an ovary-like gonad. In individuals that will become male, testicular tissue begins to develop between 21 and 30 dpf, triggering apoptosis of the ovarian tissue. Sexual determination can first be reliably established between 21 and 23 dpf, and full sexual maturity is only reached at approximately 3 months of age [PMID: 16281973; 34936027; 11914381].

Since our experiments were conducted prior to this window of sexual differentiation (between 4 and 6 dpf), the gonads remain functionally undifferentiated across all larvae, making sex determination both impossible and biologically irrelevant at this stage. Accordingly, sex is not a confounding variable in our experimental design. We have added a brief statement to this effect in the Methods section (under "Zebrafish strains and husbandry").

Editorial requirements:

1) A .docx formatted version of the manuscript text (including legends for main figures, EV figures and tables). Please make sure that the changes are highlighted to be clearly visible.

2) Individual production quality figure files as .eps, .tif, .jpg (one file per figure).

3) A .docx formatted letter INCLUDING the reviewers' reports and your detailed point-by-point responses to their comments. As part of the EMBO Press transparent editorial process, the point-by-point response is part of the Review Process File (RPF), which will be published alongside your paper.

4) A complete author checklist, which you can download from our author guidelines. Please insert information in the checklist that is also reflected in the manuscript. The completed author checklist will also be part of the RPF.

5) Please note that all corresponding authors are required to supply an ORCID ID for their name upon submission of a revised manuscript.

6) It is mandatory to include a 'Data Availability' section after the Materials and Methods. Before submitting your revision, primary datasets produced in this study need to be deposited in an appropriate public database, and the accession numbers and database listed under 'Data Availability'. Please remember to provide a reviewer password if the datasets are not yet public.

In case you have no data that requires deposition in a public database, please state so in this section. Note that the Data Availability Section is restricted to new primary data that are part of this study.

7) For data quantification: please specify the name of the statistical test used to generate error bars and P values, the number (n) of independent experiments (specify technical or biological replicates) underlying each data point and the test used to calculate p-values in each figure legend. The figure legends should contain a basic description of n, P and the test applied. Graphs must include a description of the bars and the error bars (s.d., s.e.m.).

8) At EMBO Press we ask authors to provide source data for the main manuscript figures. You will receive a separate email with instructions for providing source data with your revised manuscript, including how to upload and organize the files.

9) Our journal encourages inclusion of *data citations in the reference list* to directly cite datasets that were re-used and obtained from public databases. Data citations in the article text are distinct from normal bibliographical citations and should directly link

to the database records from which the data can be accessed. In the main text, data citations are formatted as follows: "Data ref: Smith et al, 2001" or "Data ref: NCBI Sequence Read Archive PRJNA342805, 2017". In the Reference list, data citations must be labeled with "[DATASET]". A data reference must provide the database name, accession number/identifiers and a resolvable link to the landing page from which the data can be accessed at the end of the reference.

10) We replaced Supplementary Information with Expanded View (EV) Figures and Tables that are collapsible/expandable online. A maximum of 5 EV Figures can be typeset. EV Figures should be cited as 'Figure EV1, Figure EV2" etc... in the text and their respective legends should be included in the main text after the legends of regular figures.

- For the figures that you do NOT wish to display as Expanded View figures, they should be bundled together with their legends in a single PDF file called *Appendix*, which should start with a short Table of Content. Appendix figures should be referred to in the main text as: "Appendix Figure S1, Appendix Figure S2" etc.

- Additional Tables/Datasets should be labeled and referred to as Table EV1, Dataset EV1, etc. Legends have to be provided in a separate tab in case of .xls files. Alternatively, the legend can be supplied as a separate text file (README) and zipped together with the Table/Dataset file.

11) The paper explained: EMBO Molecular Medicine articles are accompanied by a summary of the articles to emphasize the major findings in the paper and their medical implications for the non-specialist reader. Please provide a draft summary of your article highlighting
- the medical issue you are addressing,
- the results obtained and
- their clinical impact.

This may be edited to ensure that readers understand the significance and context of the research. Please refer to any of our published articles for an example.

12) Author contributions: You will be asked to provide CRediT (Contributor Role Taxonomy) terms in the submission system. These replace a narrative author contribution section in the manuscript.

13) A "Disclosure and Competing Interests Statement" should be provided in the main text.

14) Every published paper includes a 'Synopsis' to further enhance discoverability. Synopses are displayed on the journal webpage and are freely accessible to all readers. They include a short stand first (maximum of 300 characters, including space) as well as 2-5 one-sentences bullet points that summarizes the paper. Please write

the bullet points to summarize the key NEW findings. They should be designed to be complementary to the abstract - i.e. not repeat the same text. We encourage inclusion of key acronyms and quantitative information (maximum of 30 words / bullet point). Please use the passive voice. Please attach these in a separate file or send them by email, we will incorporate them accordingly.

Please also suggest a striking image or visual abstract to illustrate your article as a PNG file 550 px wide x 300 px high.

15) Include a Reagents and Tools Table as part of the Methods section, which can be downloaded from our author guidelines.

16) As part of the EMBO Publications transparent editorial process initiative (see our Editorial at http://embomolmed.embopress.org/content/2/9/329), EMBO Molecular Medicine will publish online a Review Process File (RPF) to accompany accepted manuscripts.
In the event of acceptance, this file will be published in conjunction with your paper and will include the anonymous referee reports, your point-by-point response and all pertinent correspondence relating to the manuscript. Let us know whether you agree with the publication of the RPF and as here, if you want to remove or not any figures from it prior to publication.
Please note that the Authors checklist will be published at the end of the RPF.

We confirm that we have addressed all editorial requirements as listed in the Editor's letter. Specifically:

1. A .docx formatted manuscript with all changes highlighted is provided.

2. Individual production-quality figure files (.tif) are provided for all main and Expanded View figures.

3. This point-by-point response letter is provided in .docx format.

4. A completed author checklist is provided separately.

5. ORCID IDs for all corresponding authors are provided in the submission system.

6. RNA-seq data are deposited in GEO (accession: GSE301334). Reviewer access is available via the token provided in the Data Availability section.

7. Statistical methods, replicate numbers (n), error bar definitions (SD or SEM), and p-value thresholds are now explicitly stated in all figure legends.

8. Source data for all main figures will be provided upon revised submission via the journal's source data upload system.

9. Data citations for publicly accessed datasets (Nephroseq, GEO, BroadSingleCell Portal) are included in the reference list.

10. Supplementary figures have been reorganized: 4 figures are designated as Expanded View (EV) Figures, referenced as Figure EV1–EV4 in the text. Remaining supplementary material is compiled in the Appendix PDF, referenced as Appendix Figure S1 etc.

11. A "Paper Explained" summary for non-specialist readers is provided as a separate file.

12. CRediT author contribution terms are provided in the submission system.

13. A "Disclosure and Competing Interests Statement" is included in the revised manuscript text.

14. A Synopsis (stand-first ≤ 300 characters and 2–5 bullet points) and a suggested visual abstract (PNG, 550 × 300 px) are provided as separate files.

15. A Reagents and Tools Table has been added to the Methods section.

16. We agree to the publication of the Review Process File (RPF) and do not request removal of any figures prior to publication.

We hope that our revised manuscript and these responses adequately address all concerns raised. We remain available to provide any further clarification requested by the Editor or Reviewers.

Yours sincerely,

Prof. Dr. Nicole Endlich and Dr. Felix Kliewe
On behalf of all co-authors

21st Jun 2026

Dear Dr. Kliewe,

Thank you for submitting your revised manuscript. The initial referees have now evaluated the revised version of your study. As you will see below, the referees are satisfied with the revisions, and I will therefore be able to accept your manuscript once the following points have been addressed:

1/ Manuscript text:
- Please remove the highlighted text and only keep in track changes mode any new modification.
- An email bounced for Jan Baumbach (jan.baumbach@mpi-inf.mpg.de), please check.
- Please note that all corresponding authors are required to supply an ORCID ID for their name upon submission of a revised manuscript. An ORCID identifier is missing for N. Endlich.
- Please remove "not shown" (p.23).
- Please update the headings and the order of the sections in the manuscript text to: Abstract / The Paper Explained / Introduction / Results / Discussion / Methods / Data Availability / Acknowledgements / Disclosure and Competing Interests Statement / References / Figure Legends / Expanded View Figure Legends.
- In the methods:
o Zebrafish: please provide more details on zebrafish handling and maintenance. Please report origin, as well as permit number if applicable.
o Human kidney sections: please clarify whether the Ethics committee waived the need for informed consent. Please confirm that the experiments conformed to the principles set out in the WMA Declaration of Helsinki and the Department of Health and Human Services Belmont Report.
o Antibody: please provide dilutions/concentrations.
o Statistics: please provide a statement on exclusion/inclusion criteria, blinding and randomization.
- Data Availability: please only provide in this section the information relative to deposited datasets, and remove the remaining text. Please note that the datasets must be publicly accessible before acceptance of the manuscript.
- "Disclosure statements" should be renamed "Disclosure and Competing Interests Statement". Nicole Endlich works for NIPOKA GmbH, which should be mentioned in the Disclos Statement.
- Remove the authors contributions from the manuscript text. CRediT (Contributor Role Taxonomy) terms in the submission system are sufficient.
- Our journal encourages inclusion of *data citations in the reference list* to directly cite datasets that were re-used and obtained from public databases. Data citations in the article text are distinct from normal bibliographical citations and should directly link to the database records from which the data can be accessed. In the main text, data citations are formatted as follows: "Data ref: Smith et al, 2001" or "Data ref: NCBI Sequence Read Archive PRJNA342805, 2017". In the Reference list, data citations must be labeled with "[DATASET]". A data reference must provide the database name, accession number/identifiers and a resolvable link to the landing page from which the data can be accessed at the end of the reference.

2/ Figures:
- Please remove the figures from the manuscript text and leave in only the figure legends.
- Rename the excel files "Supplement Data 1 -3" into "Dataset EV1" - "Dataset EV3" and add a legend to each excel file in a separate tab/worksheet
- Rename the "Supplement File" into "Appendix", update the heading in the file and add a table of contents with page numbers. Please correct the nomenclature from Supplemental Table 1 to Appendix Table S1, and remove the appendix figures and legends from the manuscript text.
Please note that we can accommodate more than four Expanded View (EV) figures. You are therefore welcome to convert (some of) your current Appendix figures into EV figures to improve accessibility for readers.
- There are citations for Figures S1, S4 and S5 in the manuscript text; please correct.
- Please address the queries from our data editors in the figure legends:
1. Please define the annotated p values ** as well as provide the exact p-values for the same in the legend of figure 6C as appropriate.
2. Please note that the exact p values are not provided in the legends of 6I; 7D,F,G,H; EV-1A; EV-2A-C; EV-3A;
3. Please indicate the statistical test used for data analysis in the legends of figures 2A-C,G,H; 3A-C,G,H; 4A,F;
4. Please note that the box plots need to be defined in terms of minima, maxima, centre, bounds of box and whiskers, and percentile in the legends of figures 7I,J; EV-1A,C.
5. Please note that information related to n is missing in the legends EV-4C,D.

3/ Author checklist:
- Please check the section Experimental animals/animal observed in or captured from the field, as it doesn't appear to apply to your study.
- Please fill in Experimental study design and statistics/inclusion-exclusion criteria.
- Please fill in Ethics/authority granting ethics approval and informed consent/Helsinki declaration.

4/ Source Data:
Please provide the filled Source Data checklist. Please upload the Source Data as 1 zip file per figure, with the different panels in subfolders. Image source data should be provided.

5/ Synopsis:
The visual abstract should be simplified and resized to 550 pixels wide x 300 pixels high. Please make sure the text remains legible.

We note that you agree with the publication of the Review Process File.

I look forward to receiving your revised manuscript.

Yours sincerely,

Lise Roth

Lise Roth, PhD

Senior Editor

EMBO Molecular Medicine

***** Reviewer's comments *****

Referee #1 (Comments on Novelty/Model System for Author):

The comments and points raised have been correctly addressed by the authos. This does not change my initial appreciation of the manuscript.

Referee #1 (Remarks for Author):

I have no further comments.

Referee #2 (Comments on Novelty/Model System for Author):

It is a well performed study and the author has produced high quality images. The novelty and clinical impact of this study remains moderate as it focuses on events that are also prevalnet in disease conditions other than FSGS.

Referee #2 (Remarks for Author):

The author has addressed all concerns.

Referee #3 (Comments on Novelty/Model System for Author):

Per my original evaluation, I believe that study demonstrated a clear parallel between the chosen model organism and human condition, and therefore the study using the model provides useful insights with medical relevance. This evaluation remains the same for this revision.

Referee #3 (Remarks for Author):

The authors have addressed all of my concerns and questions and have also largely addressed comments from other reviewers that I feel are essential to address.

The authors have addressed all minor editorial requests.

15th Jul 2026

Dear Dr. Kliewe,

Thank you for submitting your revised files. I am pleased to inform you that your manuscript is accepted for publication and is now being sent to our publisher to be included in the next available issue of EMBO Molecular Medicine.

Your manuscript will be processed for publication by EMBO Press. It will be copy edited and you will receive page proofs prior to publication. Please note that you will be contacted by Springer Nature Author Services to complete licensing and payment information.

You may qualify for financial assistance for your publication charges - either via a Springer Nature fully open access agreement or an EMBO initiative. Check your eligibility: https://link.springer.com/journal/44321/how-to-publish-with-us

Should you be planning a Press Release on your article, please notify the production team via this form in order to coordinate publication and release dates: https://forms.cloud.microsoft/e/eH4A8JVP6g

If you have any questions, please do not hesitate to contact the Editorial Office.

Thank you for your contribution to EMBO Molecular Medicine.

Yours sincerely,

Lise Roth

Lise Roth, Ph.D
Senior Editor
EMBO Molecular Medicine

>>> Please note that it is EMBO Molecular Medicine policy for the transcript of the editorial process (containing referee reports and your response letter) to be published as an online supplement to each paper. If you do NOT want this, you will need to inform the Editorial Office via email immediately. More information is available here: https://link.springer.com/partners/embo-press/editorial-policies#Peer%20review

EMBO Press Author Checklist

Corresponding Author Name: Felix Kliewe	
Journal Submitted to: EMBO Molecular Medicine	
Manuscript Number: EMM-2026-23327-V2-Q	

USEFUL LINKS FOR COMPLETING THIS FORM
The EMBO Journal - Author Guidelines
EMBO Reports - Author Guidelines
Molecular Systems Biology - Author Guidelines
EMBO Molecular Medicine - Author Guidelines

Reporting Checklist for Life Science Articles (updated January
This checklist is adapted from Materials Design Analysis Reporting (MDAR) Checklist for Authors. MDAR establishes a minimum set of requirements in transparent reporting in the life sciences (see Statement of Task: 10.31222/osf.io/9sm4x). Please follow the journal's guidelines in preparing your
Please note that a copy of this checklist will be published alongside your article.

Abridged guidelines for figures
1. Data
The data shown in figures should satisfy the following conditions:
- the data were obtained and processed according to the field's best practice and are presented to reflect the results of the experiments in an accurate and unbiased manner.
- ideally, figure panels should include only measurements that are directly comparable to each other and obtained with the same assay.
- plots include clearly labeled error bars for independent experiments and sample sizes. Unless justified, error bars should not be shown for technical
- if n<5, the individual data points from each experiment should be plotted. Any statistical test employed should be justified.
- Source Data should be included to report the data underlying figures according to the guidelines set out in the authorship guidelines on Data

2. Captions
Each figure caption should contain the following information, for each panel where they are relevant:
- a specification of the experimental system investigated (eg cell line, species name).
- the assay(s) and method(s) used to carry out the reported observations and measurements.
- an explicit mention of the biological and chemical entity(ies) that are being measured.
- an explicit mention of the biological and chemical entity(ies) that are altered/varied/perturbed in a controlled manner.
- the exact sample size (n) for each experimental group/condition, given as a number, not a range;
- a description of the sample collection allowing the reader to understand whether the samples represent technical or biological replicates (including how many animals, litters, cultures, etc.).
- a statement of how many times the experiment shown was independently replicated in the laboratory.
- definitions of statistical methods and measures:
  - common tests, such as t-test (please specify whether paired vs. unpaired), simple χ2 tests, Wilcoxon and Mann-Whitney tests, can be unambiguously identified by name only, but more complex techniques should be described in the methods section;
  - are tests one-sided or two-sided?
  - are there adjustments for multiple comparisons?
  - exact statistical test results, e.g., P values = x but not P values < x;
  - definition of 'center values' as median or average;
  - definition of error bars as s.d. or s.e.m.

Please complete ALL of the questions below.
Select "Not Applicable" only when the requested information is not relevant for your study.

Materials

Newly Created Materials	Information included in the manuscript?	In which section is the information available? (Reagents and Tools Table, Materials and Methods, Figures, Data Availability Section)
New materials and reagents need to be available; do any restrictions apply?	Not Applicable	

Antibodies	Information included in the manuscript?	In which section is the information available? (Reagents and Tools Table, Materials and Methods, Figures, Data Availability Section)
For **antibodies** provide the following information:<br>- Commercial antibodies: RRID (if possible) or supplier name, catalogue number and or/clone number<br>- Non-commercial: RRID or citation	Yes	Reagents and Tools Table

DNA and RNA sequences	Information included in the manuscript?	In which section is the information available? (Reagents and Tools Table, Materials and Methods, Figures, Data Availability Section)
**Short novel DNA or RNA including primers, probes**: provide the sequences.	Yes	Used qRT-PCR Primer: Supplementary Table 1

Cell materials	Information included in the manuscript?	In which section is the information available? (Reagents and Tools Table, Materials and Methods, Figures, Data Availability Section)
**Cell lines:** Provide species information, strain. Provide accession number in repository **OR** supplier name, catalog number, clone number, and/**OR** RRID.	Not Applicable	
**Primary cultures:** Provide species, strain, sex of origin, genetic modification status.	Not Applicable	
Report if the cell lines were recently **authenticated** (e.g., by STR profiling) and tested for mycoplasma contamination.	Not Applicable	

Experimental animals	Information included in the manuscript?	In which section is the information available? (Reagents and Tools Table, Materials and Methods, Figures, Data Availability Section)
**Laboratory animals or Model organisms:** Provide species, strain, sex, age, genetic modification status. Provide accession number in repository **OR** supplier name, catalog number, clone number, **OR** RRID.	Yes	Materials and Methods
**Animal observed in or captured from the field:** Provide species, sex, and age where possible.	Not Applicable	
Please detail **housing and husbandry conditions**.	Yes	Materials and Methods

Plants and microbes	Information included in the manuscript?	In which section is the information available? (Reagents and Tools Table, Materials and Methods, Figures, Data Availability Section)
**Plants:** provide species and strain, ecotype and cultivar where relevant, unique accession number if available, and source (including location for collected wild specimens).	Not Applicable	
**Microbes:** provide species and strain, unique accession number if available, and source.	Not Applicable	

Human research participants	Information included in the manuscript?	In which section is the information available? (Reagents and Tools Table, Materials and Methods, Figures, Data Availability Section)
If collected and within the bounds of privacy constraints report on age, sex and gender or ethnicity for all study participants.	Not Applicable	

Core facilities	Information included in the manuscript?	In which section is the information available? (Reagents and Tools Table, Materials and Methods, Figures, Data Availability Section)
If your work benefited from core facilities, was their service mentioned in the acknowledgments section?	Not Applicable	

Design

Study protocol	Information included in the manuscript?	In which section is the information available? (Reagents and Tools Table, Materials and Methods, Figures, Data Availability Section)
If study protocol has been **pre-registered, provide DOI in the manuscript**. For clinical trials, provide the trial registration number **OR** cite DOI.	Not Applicable	
Report the **clinical trial registration number** (at ClinicalTrials.gov or equivalent), where applicable.	Not Applicable	

Laboratory protocol	Information included in the manuscript?	In which section is the information available? (Reagents and Tools Table, Materials and Methods, Figures, Data Availability Section)
Provide DOI OR other citation details if **external detailed step-by-step protocols** are available.	Not Applicable	

Experimental study design and statistics	Information included in the manuscript?	In which section is the information available? (Reagents and Tools Table, Materials and Methods, Figures, Data Availability Section)
Include a statement about **sample size** estimate even if no statistical methods were used.	Yes	Figure legends
Were any steps taken to minimize the effects of subjective bias when allocating animals/samples to treatment (e.g. **randomization procedure**)? If yes, have they been described?	Yes	Materials and Methods
Include a statement about **blinding** even if no blinding was done.	Yes	Materials and Methods
Describe **inclusion/exclusion criteria** if samples or animals were excluded from the analysis. Were the criteria pre-established?<br><br>If sample or data points were omitted from analysis, report if this was due to attrition or intentional exclusion and provide justification.	Yes	Materials and Methods
For every figure, are **statistical tests** justified as appropriate? Do the data meet the assumptions of the tests (e.g., normal distribution)? Describe any methods used to assess it. Is there an estimate of variation within each group of data? Is the variance similar between the groups that are being statistically compared?	Yes	Figure legends, Materials and methods

Sample definition and in-laboratory replication	Information included in the manuscript?	In which section is the information available? (Reagents and Tools Table, Materials and Methods, Figures, Data Availability Section)
In the figure legends: state number of times the experiment was **replicated** in laboratory.	Yes	Figure legends
In the figure legends: define whether data describe **technical or biological replicates**.	Yes	Figure legends

Ethics

Ethics	Information included in the manuscript?	In which section is the information available? (Reagents and Tools Table, Materials and Methods, Figures, Data Availability Section)
Studies involving **human participants**: State details of **authority granting ethics approval** (IRB or equivalent committee(s), provide reference number for approval.	Not Applicable	
Studies involving **human participants**: Include a statement confirming that **informed consent** was obtained from all subjects and that the experiments conformed to the principles set out in the WMA Declaration of Helsinki and the Department of Health and Human Services Belmont Report.	Yes	Materials and Methods
Studies involving **human participants:** For publication of **patient photos**, include a statement confirming that consent to publish was obtained.	Not Applicable	
Studies involving experimental **animals**: State details of **authority granting ethics approval** (IRB or equivalent committee(s), provide reference number for approval. Include a statement of compliance with ethical regulations.	Yes	Materials and Methods
Studies involving **specimen and field samples:** State if relevant **permits** obtained, provide details of authority approving study; if none were required, explain why.	Not Applicable	

Dual Use Research of Concern (DURC)	Information included in the manuscript?	In which section is the information available? (Reagents and Tools Table, Materials and Methods, Figures, Data Availability Section)
Could your study fall under dual use research restrictions? Please check biosecurity documents and list of **select agents and toxins** (CDC): https://www.selectagents.gov/sat/list.htm	Not Applicable	
If you used a select agent, is the security level of the lab appropriate and reported in the manuscript?	Not Applicable	
If a study is subject to dual use research of concern regulations, is the name of the **authority granting approval and reference number** for the regulatory approval provided in the manuscript?	Not Applicable	

Reporting

The MDAR framework recommends adoption of discipline-specific guidelines, established and endorsed through community initiatives. Journals have their own policy about requiring specific guidelines and recommendations to complement MDAR.

Adherence to community standards	Information included in the manuscript?	In which section is the information available? (Reagents and Tools Table, Materials and Methods, Figures, Data Availability Section)
State if relevant guidelines or checklists (e.g., **ICMJE, MIBBI, ARRIVE, PRISMA**) have been followed or provided.	Not Applicable	
For **tumor marker prognostic studies,** we recommend that you follow the **REMARK** reporting guidelines (see link list at top right). See author guidelines, under 'Reporting Guidelines'. Please confirm you have followed these guidelines.	Not Applicable	
For **phase II and III randomized controlled trials**, please refer to the **CONSORT** flow diagram (see link list at top right) and submit the CONSORT checklist (see link list at top right) with your submission. See author guidelines, under 'Reporting Guidelines'. Please confirm you have submitted this list.	Not Applicable	

Data Availability

Data availability	Information included in the manuscript?	In which section is the information available? (Reagents and Tools Table, Materials and Methods, Figures, Data Availability Section)
Have **primary datasets** been deposited according to the journal's guidelines (see 'Data Deposition' section) and the respective accession numbers provided in the Data Availability Section?	Yes	Data Availability Section; Source Data File
Were **human clinical and genomic datasets** deposited in a public access-controlled repository in accordance to ethical obligations to the patients and to the applicable consent agreement?	Not Applicable	
Are **computational models** that are central and integral to a study available without restrictions in a machine-readable form? Were the relevant accession numbers or links provided?	Not Applicable	
If publicly available data were reused, provide the respective **data citations in the reference list**.	Yes	Materials and Methods, Results, Figure Legend and Reference List
